# Synthesis of triazole-linked morpholino oligonucleotides *via* Cu^I^ catalysed cycloaddition†
†Electronic supplementary information (ESI) available: Full experimental details and copies of ^1^H, ^13^C, ^31^P NMR spectra for all compounds. See DOI: 10.1039/c6ob00007j



**DOI:** 10.1039/c6ob00007j

**Published:** 2016-02-16

**Authors:** Matthew J. Palframan, Rima D. Alharthy, Paulina K. Powalowska, Christopher J. Hayes

**Affiliations:** a School of Chemistry , University of Nottingham , University Park , Nottingham , NG7 2RD , UK . Email: chris.hayes@nottingham.ac.uk ; Tel: +44 (0)115 951 3045

## Abstract

Triazole-linked morpholino (^TL^MO) oligonucleic acids were synthesised using the Cu^I^ catalysed (3 + 2) azide–alkyne cycloaddition (CuAAC) reaction. The modified DNA analogues were incorporated into 13-mer sequences *via* solid phase synthesis. UV melting experiments showed that the ^TL^MO modification gives higher *T*_m_ values than the corresponding ^TL^DNA modification.

## Abstract 2

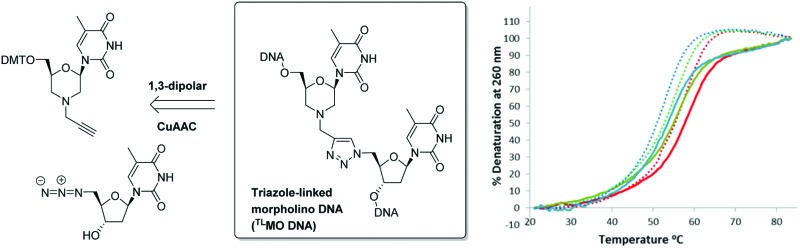
Triazole-linked morpholino (^TL^MO) oligonucleic acids were synthesised using the Cu^I^ catalysed (3 + 2) azide–alkyne cycloaddition (CuAAC) reaction.

## Introduction

Click chemistry has recently emerged as a powerful tool in the field of nucleic acid research.^1^ In particular, the Cu^I^ catalysed (3 + 2) azide–alkyne cycloaddition (CuAAC)^2^ has been used to construct modified internucleotide linkages,^3^ to prepare nucleic acid conjugates,^4^ and as a strand ligation tool.^5^ Zerrouki *et al.*, designed a novel triazole-linked DNA analogue (^TL^DNA) **1** using the CuAAC for oligomer elongation,^6^ and this preliminary work has been significantly extended by Brown *et al*. The artificial ^TL^DNA retains good aqueous solubility, is stable towards enzymatic degradation,^3*a*^ and can be read by polymerases,^7^ thus making it capable of *in vitro* transcription^8^ and rolling circle amplification.^7^ Furthermore, and perhaps most impressively, Brown has demonstrated that genes containing ^TL^DNA **1** are functional *in vivo* in *Escherichia coli* and in human cells.^9^ Given the biocompatibility of the ^TL^DNA **1** with DNA processing enzymes, it is curious that the thermal stability of complementary duplexes is reduced. A recent study on the structural basis of this phenomenon reported that the ^TL^DNA modification leads to less optimal stacking interactions and distortion in the backbone at, and adjacent to, the site of the triazole.^10^ Whilst high melting temperatures are not required for all uses of modified nucleic acids, the formation of stable duplexes is a requirement for therapeutic applications of oligonucleotides, and as such ^TL^DNAs **1** do not represent good drug candidates.

**Fig. 1:**
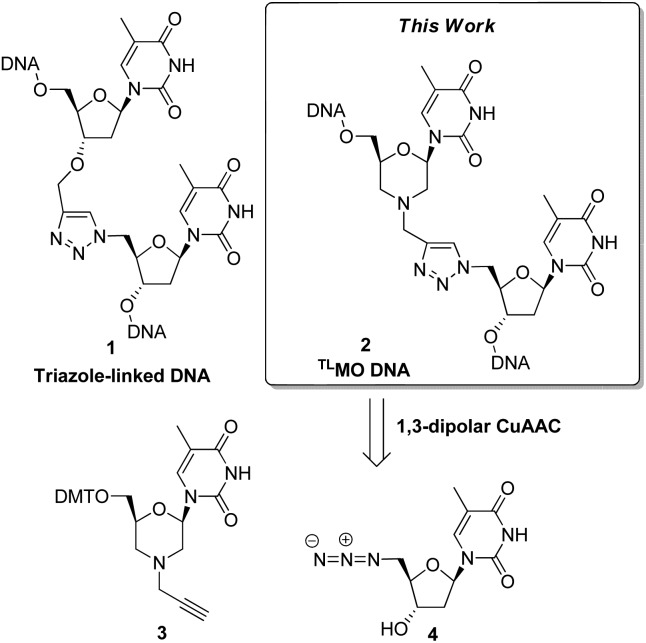
Approach to triazole-linked morpholino (^TL^MO) hybrid structures **2**.

As part of our own research aimed at developing therapeutic nucleic acids, we wondered if the thermal stability of triazole-containing duplexes could be improved by the addition of further modifications to the backbone. Thus we decided to examine triazole-linked morpholino (^TL^MO) hybrid structures **2** (Fig. 1) as they could combine the ease of synthesis of the ^TL^DNAs **1** with the increased melting temperatures associated with morpholino drug candidates.^11,12^ The ^TL^MO hybrid **2** can be disconnected to reveal the azide **4** and the alkyne-substituted morpholine **3** as potential precursors for the proposed CuAAC reaction (Fig. 1).

**Scheme 1:**
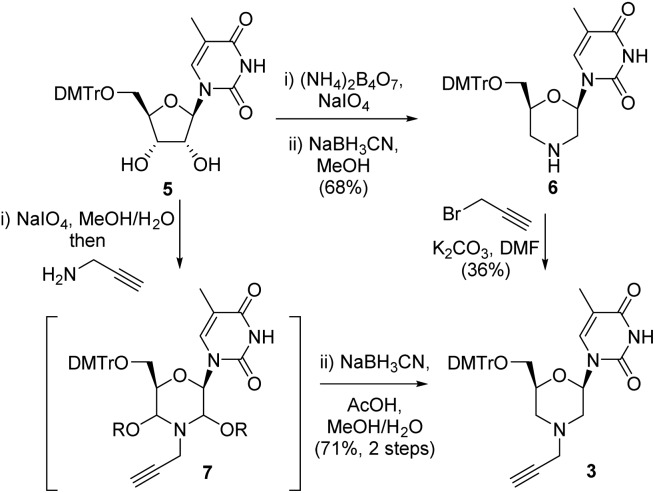
Synthesis of the alkyne morpholine nucleoside **3**.

Our initial route to **3** proceeded *via* the morpholine **6**, which was readily prepared from 5-methyl uridine **5** in good yield by oxidative cleavage and subsequent reductive amination^13^ (Scheme 1). Although the *N*-alkylation of **6** did produce the desired *N*-propargyl morpholine **3**, only a low yield (36%) of the desired alkyne was obtained. The main side reaction was over alkylation of the thymine base in addition to *N*-alkylation of the morpholine, and an alternative route was explored. Thus, oxidative cleavage of **5** in the presence of propargylamine first gave **7**, which upon treatment with sodium cyanoborohydride/AcOH gave the desired product **3** in good overall yield (71%) (Scheme 1).

**Scheme 2:**
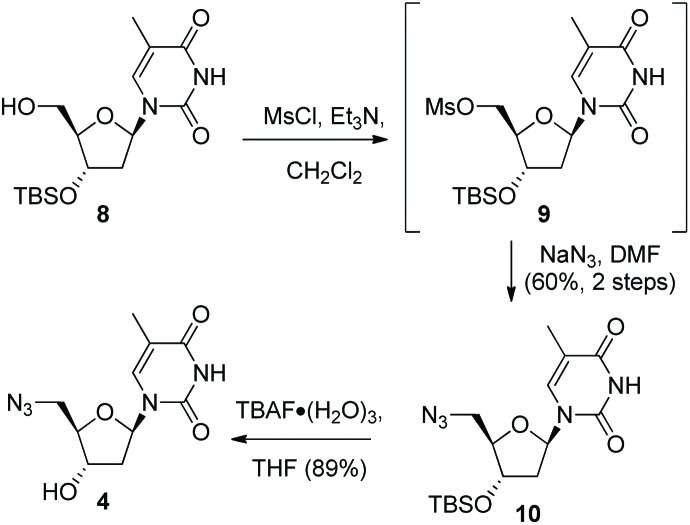
Synthesis of 5′-deoxy-5′-azido thymidine **4**.

Following formation of the alkyne-containing morpholine nucleoside **3**, our attention then turned to the synthesis of the azide cycloaddition partner. Pleasingly, the 3′-TBSO-protected-5′-deoxy-5′-azido thymidine **10** was readily prepared from the alcohol **8** *via* a two-step sequence involving mesylate formation and displacement with sodium azide. The 3′-OH-5′-deoxy-5′-azido thymidine **4** was then synthesised from **10** *via* TBAF deprotection (Scheme 2). Alternatively, 4 could be accessed directly from thymidine *via* selective 5′-tosylation and subsequent azide displacement using the known procedure (38%, 2 steps).^14^

**Scheme 3:**
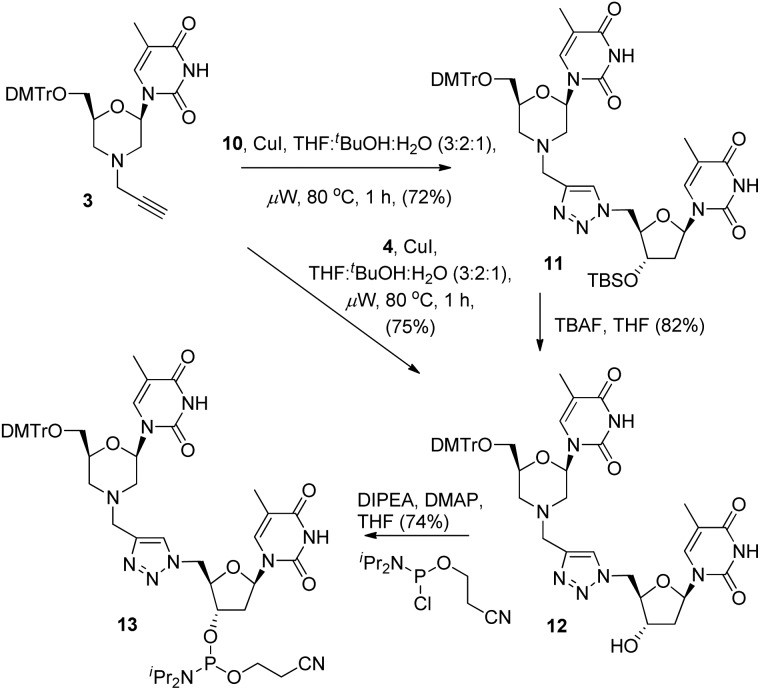
Synthesis of 3′-cyanoethyl phosphoramidite **13**.

With the required coupling partners in hand, we next examined the key Cu-catalysed cycloaddition step. As we needed access the 3′-alcohol **12** to prepare phosphoramidite **13**, we explored two routes for its synthesis. The first was cycloaddition of **3** and the protected azide **10** (*i.e.* **3** + **10** → **11**), followed by TBS deprotection (**11** → **12**), and the second was initial deprotection (**10** → **4**, Scheme 2) followed by cycloaddition with **3**. A range of catalysts and solvents were initially screened, and it was quickly found that the use of copper(i) iodide in THF : ^*t*^BuOH : H_2_O (3 : 2 : 1) with microwave heating (80 °C) was optimal (Scheme 3).^3*a*^ Under these conditions, cycloaddition of the acetylene **3** with the TBS-protected azide **10** gave the triazole-linked morpholino (^TL^MO) dimer **11** in good yield, and TBAF deprotection of **11** gave the desired alcohol **12** in good yield (Scheme 3). We were pleased to find that the alternative cycloaddition of **4** with **3** also proceeded in good yield to give the alcohol **12** directly, and this was adopted as our favoured route due to an improved overall yield and easier of purification of **12** by column chromatography. Finally, the ^TL^MO **12** was converted to the 3′-cyanoethyl phosphoramidite **13** (74%) under standard conditions (Scheme 3).

**Scheme 4:**
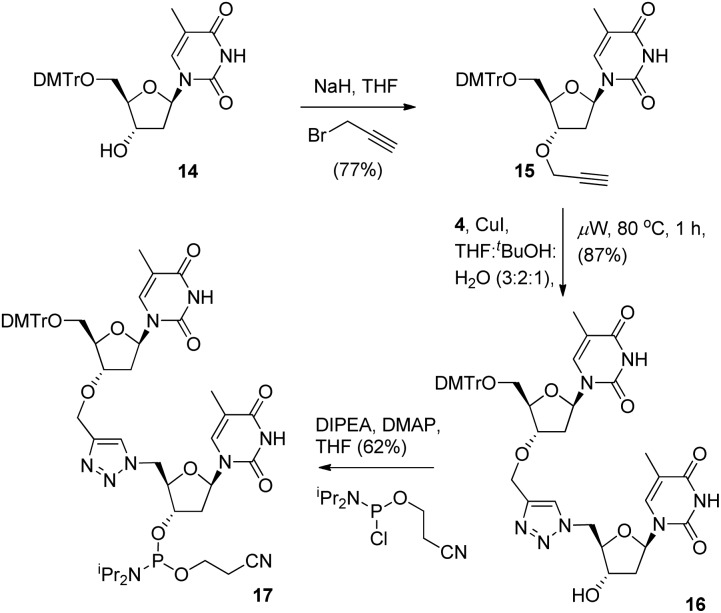
Synthesis of 3′-cyanoethyl phosphoramidite **17**.

To provide a direct comparison of the new ^TL^MO hybrid **2** to the triazole-linked DNA analogue (^TL^DNA) **1**, we next prepared the phosphoramidite **17**. This reagent facilitates incorporation of the triazole modification **1** into oligonucleotide sequences *via* solid-phase synthesis as opposed to the fragment ligation method used previously by Brown *et al.* ^10^ The phosphoramidite **17** was readily prepared from **14** *via* 3′-*O*-alkylation to give the alkyne **15**, Cu-catalysed cycloaddition with **4** to provide the triazole-containing dimer **16** and then conversion to **17** in the usual manner (Scheme 4).

Pleasingly, the modified phosphoramidites **13** and **17** were fully compatible with solid-phase oligonucleotide synthesis and we prepared the ^TL^MO-containing oligomer **21** and the known ^TL^DNA oligomer **22** ^10^ in good yield (Table 1). Stock aqueous solutions (pH 7) of the oligomers **21** and **22** were readily prepared, and no adverse solubility issues were observed. As Brown *et al.* have already reported UV-melting data of **22** duplexed with its complimentary DNA strand **18**,^10^ we also prepared **18** so that we could directly compare the *T*_m_ values of **18** + **21**, **18** + **22** and the unmodified duplex (**18** + **20**) under the same conditions. In order to assess the potential use of the ^TL^MO-modification in therapeutically useful oligomers, we also synthesised the complimentary RNA oligonucleotide **19**, as this simulates an intracellular mRNA target. The integrity of the oligomers **18–22** was confirmed by ESI mass spectrometry (Table 1) and HPLC (see ESI†).

**Table 1:** Sequences of oligonucleotides synthesised

Identifier	Sequence 5′ → 3′	*m*/*z*	
		Calculated	Found
18 (DNA)	d(GCTGCAAACGTCG)	3953.55	3953.73
19 (RNA)	GCUGCAAACGUCG	4133.49	4133.63
**20**	d(CGACGTTTGCAGC)	3944.53	3944.72
**21** ^*a*^	d(CGACG 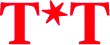 TGCAGC)	3944.64	3944.80
**22** ^*b*^	d(CGACG 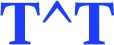 TGCAGC)	3945.63	3945.78

^*a*^ 
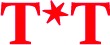
 indicates the position of the morpholine-triazole modification **13**.

^*b*^ 
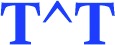
 indicates the position of the triazole modification **17**.

**Fig. 2:**
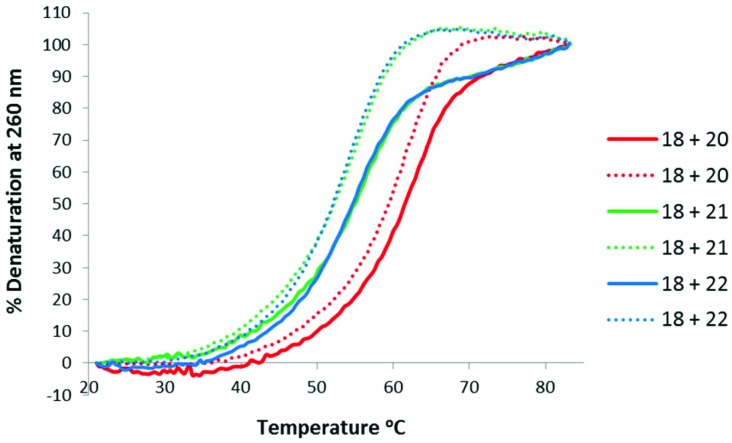
UV-melting curves for oligomers (3 μM) **20**, **21** and **22** duplexed with DNA **18**. Dotted lines represent cooling curves.

**Fig. 3:**
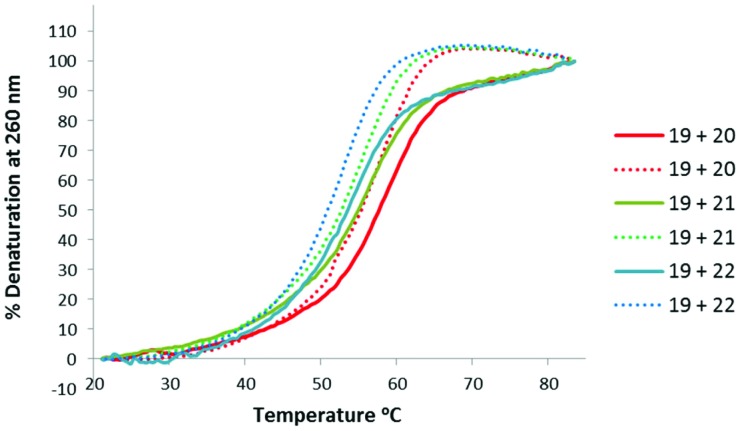
UV-melting curves for oligomers (3 μM) **20**, **21** and **22** duplexed with RNA **19**. Dotted lines represent cooling curves.

Thermal stabilities of the ^TL^MO **21**, ^TL^DNA **22**, and unmodified DNA **20** duplexed with complimentary DNA **18** (Fig. 2) and RNA **19** (Fig. 3) were then determined by UV melting experiments^15^ (Table 2). Pleasingly, the *T*_m_ values of the control DNA **18** : DNA **20** (62.4 °C) (entry 1, Table 2), and the DNA **18** : ^TL^DNA **22** (55.1 °C) (entry 3, Table 2) duplexes were in close agreement with those reported previously by Brown (62.89 °C and 55.30 °C respectively).^10^ The ^TL^MO-containing oligomer **21** duplexed to DNA **18** gave a *T*_m_ value of 56.1 °C (entry 2, Table 2), which represents a small increase (Δ*T*_m_ 1.0 °C) over that determined for **22**, but still represents a significant decrease from the unmodified DNA (Δ*T*_m_ –6.3 °C). As mentioned above, duplexes with RNA provide a more meaningful comparison for future therapeutic applications and the *T*_m_ value of RNA **19** duplexed with unmodified DNA **20** was determined (58.5 °C) as a control (entry 4, Table 2). In contrast to the duplexes with DNA, the *T*_m_ of ^TL^MO **21** (56.6 °C) was much closer to that of the unmodified DNA : RNA than was ^TL^DNA **22** (54.1 °C) with RNA **19** (Δ*T*_m_ –1.9 °C for **21** *vs.* –4.4 °C for **22**) (entries 5 and 6, Table 2), thus demonstrating that the addition of the morpholine modification can regain half of the *T*_m_ lost by incorporating the triazole internucleotide linkage.

**Table 2:** Thermal melting (*T*_m_) data for oligonucleotide duplexes

Entry	Oligomers	*T*_m_ ^*a*^ °C	*T*_m_ ^*b*^
1	**18** + **20**	62.4 (61.9)	N/A
2	**18** + **21**	56.1 (54.7)	–6.3 (–7.2)
3	**18** + **22**	55.1 (54.1)	–7.3 (–7.8)
4	**19** + **20**	58.5 (58.2)	N/A
5	**19** + **21**	56.6 (55.3)	–1.9 (–2.9)
6	**19** + **22**	54.1 (52.8)	–4.4 (–5.4)

^*a*^ *T*_m_ values for 3 μM oligo samples. Values in parentheses refer to cooling curves.

^*b*^Δ*T*_m_ per modification relative to the control DNA **20**.

Further structural studies are underway in order to fully assess the duplexes formed by ^TL^MO-modified oligomers, before selecting the best candidates for biological evaluation.

## Experimental¶
¶For general experimental details please see the ESI.†



### 5′-*O*-DMTr-morpholino thymidine (**6**)

5′-*O*-DMTr-5-methyluridine **5** (700 mg, 1.25 mmol) was dissolved in dry MeOH (10 mL) under an argon atmosphere. Ammonium biborate (328 mg, 2.50 mmol), sodium periodate (535 mg, 2.50 mmol) were added to the reaction mixture. After stirring for 3 h at room temperature, the mixture was filtered and sodium cyanoborohydride (155 mg, 2.50 mmol) was added to the filtrate in one portion with stirring. Stirring continued for 6 h followed by evaporation to afford a residue, which was dissolved in EtOAc (10 mL), washed with brine (3 × 10 mL). The organic phase was dried over MgSO_4_, filtered and evaporated and purified by column chromatography eluting with (CHCl_3_ : MeOH, 25 : 1) to afford **6** as a colourless foam (460 mg, 68% over three steps); *R*_f_ 0.28 (CHCl_3_ : MeOH, 25 : 1); [*α*]25D +47 (*c* 0.61, CHCl_3_); *ν*_max_/cm^–1^ (CHCl_3_) 3389, 2933, 2838, 2103, 1684, 1609, 1487 and 1455; ^1^H NMR (400 MHz, CDCl_3_) 7.47–7.42 (2H, m, Ar), 7.36–7.27 (6H, m, Ar), 7.32 (1H, s, C6*H*), 7.24–7.19 (1H, m, Ar), 6.84 (4H, d, *J* 8.9, Ar), 5.77 (1H, dd, *J* 10.0, 2.7, C1′*H*), 4.01(1H, dtd, *J* 10.7, 4.9, 2.2, C4′*H*), 3.79 (6H, s, OCH_3_), 3.27 (1H, dd, *J* 9.7, 5.1, C5′HH), 3.15 (1H, dd, *J*, 12.5, 2.7, C2′HH), 3.11–3.02 (2H, m, C5′HH and C3′HH), 2.68–2.58 (2H, m, C3′HH, C2′HH), 1.95 (3H, s, CH_3_); ^13^C NMR (100 MHz, CDCl_3_) 164.1 (C), 158.5 (C), 150.4 (C), 144.8 (C), 135.9 (C), 135.8 (C), 135.4 (CH), 130.1 (CH), 130.0 (CH), 128.1 (CH), 127.8 (CH), 126.9 (CH), 113.1 (CH), 110.9 (C), 86.1 (C), 80.5 (CH), 78.0 (CH), 64.5 (CH_2_), 55.2 (CH_3_), 49.0 (CH_2_), 46.9 (CH_2_), 12.9 (CH_3_); HRMS *m*/*z* (ES+) Found 566.2245 (M + Na, C_31_H_33_N_3_NaO_6_ requires 566.2245).

### *N*′-propargyl-5′-*O*-DMTr-morpholino thymidine (**3**)

To a stirred solution of 5′-*O*-DMTr 5-methyluridine (**5**) (725 mg, 1.29 mmol) in MeOH (12 mL) under an argon atmosphere, was added a solution of sodium periodate (304 mg, 1.42 mmol) in water (2 mL) dropwise over 5 min, followed by propargyl amine (103 μL, 1.62 mmol) in one portion. The resulting solution was stirred at room temperature for 3 hours, during which time a white precipitate formed, the mixture was filtered. To the stirred solution of the filtrate was added sodium cyanoborohydride (162 mg, 2.58 mmol) followed by the dropwise addition of acetic acid (110 μL, 1.93 mmol). The reaction was stirred for 12 h at room temperature. The volatile organic were removed by evaporation. The residue was partitioned between sat. NaHCO_3_ (50 mL) and EtOAc (50 mL), the aqueous layer was extracted with EtOAc (50 mL). The combined organic layers were washed with brine (3 × 50 mL), dried over MgSO_4_, and evaporated. The residue was purified by silica gel chromatography, eluting with DCM : MeOH (40 : 1) to afford the title compound (529 mg, 71%) as a colourless foam; *R*_f_ 0.17 (DCM : MeOH 25 : 1); [*α*]25D +30 (*c* 0.93, CHCl_3_); *ν*_max_/cm^–1^ (CHCl_3_) 3390, 3196, 2955, 1933, 1838, 1694, 1633, 1609, 1583, 1491, 1456; ^1^H NMR (400 MHz, chloroform-d) *δ* 9.95 (1H, br s, N*H*), 7.48–7.44 (2H, m, Ar), 7.37–7.27 (6H, m, Ar), 7.32 (1H, s, C6*H*), 7.24–7.20 (1H, m, Ar), 6.84 (4H, d, *J* 8.9, Ar), 5.93 (1H, dd, *J* 9.8, 2.7, C1′H), 4.12 (1H, m, C4′H), 3.79 (6H, s, OCH_3_), 3.45–3.44 (2H, m, NC*H*_2_C≡CH), 3.34 (1H, dd, *J* 9.6, 5.3, C5′HH), 3.11 (1H, dd, *J* 9.6, 5.4, C5′H*H*), 2.97 (1H, br d, *J* 10.5, C3′*H*_*A*_H_B_), 2.84 (1H, br d, *J* 11.4, C2′HH), 2.32 (1H, t, *J* 2.3, C≡CH), 2.34–2.23 (2H, m, C3′HH, C2′HH), 1.96 (3H, s, C*H*_3_); ^13^C NMR (101 MHz, chloroform-d) 164.1 (C), 158.6 (C), 150.3 (C), 144.8 (C), 136.0 (C), 135.8 (C), 135.6 (CH), 130.13 (CH), 130.09 (CH), 128.2 (CH), 127.9 (CH), 126.9 (CH), 113.2 (CH), 110.9 (C), 86.2 (C), 79.6 (CH), 77.4 (C), 75.6 (CH), 74.5 (CH), 64.6 (CH_2_), 55.3 (CH_3_), 54.6 (CH_2_), 52.8 (CH_2_), 46.4 (CH_2_), 12.7 (CH_3_); ^1^H NMR (400 MHz, Benzene-d_6_) *δ* 9.88 (1H, s, NH), 7.68 – 7.59 (2H, m, Ph), 7.52–7.40 (4H, m, Ar), 7.23–7.15 (2H, m, Ph), 7.11 – 7.01 (1H, m, Ph), 6.94 (1H, q, *J* 1.2, C6H), 6.81–6.71 (4H, m, Ar), 5.95 (1H, dd, *J* 9.7, 2.7, C1′H), 3.96–3.90 (1H, m, C4′H), 3.38 (1H, dd, *J* 9.6, 5.4, C5′HH), 3.30 (3H, s, OCH_3_), 3.29 (3H, s, OCH_3_) 3.17 (1H, dd, *J* 9.6, 5.1, C5′HH), 3.01 (1H, dd, *J* 17.5, 2.4, NC*H*HC≡CH), 2.92 (1H, dd, *J* 17.5, 2.4, NCH*H*C≡CH), 2.63 (1H, d, *J* 10.4, C2′HH), 2.47 (1H, d, *J* 10.9, C3′HH), 2.16 (1H, t, *J* 11.0, C3′HH), 2.16 (1H, t, *J* 10.4, C3′HH), 1.90 (1H, t, *J* 2.4, C≡C*H*), 1.66 (3H, d, *J* 1.2, C*H*_3_); ^13^C NMR (101 MHz, Benzene-d_6_) *δ* 163. 9 (C4), 159.3 (2 × ^Ar^C), 150.3 (C2), 145.7 (^Ph^C), 136.4 (^Ar^C), 136.3 (^Ar^C), 135.1 (C6H), 130.6 (4 × ^Ar^CH), 128.7 (2 × PhCH), 127.9 (2 × ^Ph^CH), 127.2 (^Ph^CH), 113.6 (4 × ArCH), 110.8 (C5H), 86.7 (Ar_3_CO), 80.2 (C1′H), 78.0 (C), 75.9 (C4′H), 74.3 (CH), 65.1 (C5′H2), 54.8 (2 × OCH_3_ and C2′H_2_), 52.6 (C3′H_2_), 46.3 (NCH_2_C), 12.6 (CH_3_). HRMS (ESI) C_34_H_36_N_3_O_6_ (M + H^+^) requires 582.2599, found 582.2569.

### 5′-*O*-Mesyl-3′-*O-tert*-butyldimethylsilyl deoxythymidine (**9**)

To a stirred solution of 5′-OH-3′-*O-tert*-butyldimethylsilyl deoxythymidine (1.06 g, 3.0 mmol) in dichloromethane (15 mL) at 0 °C was added triethylamine (0.84 mL, 6.0 mmol) followed by the dropwise addition of mesyl chloride (277 μL, 3.6 mmol). The resulting solution was stirred at 0 °C for 1 h, then warmed to room temperature, and stirred for a further 3 hours. The reaction was quenched by the addition of water (50 mL), the layers were separated, and the aqueous layer was extracted with DCM (2 × 50 mL). The combined organic layers were washed with sat NH_4_Cl (50 mL), sat. NaHCO_3_ (50 mL), brine (50 mL), dried over MgSO_4_, and evaporated to afford the title compound (1.29 g, Quant.) as a yellow foam, which was used without further purification; *ν*_max_/cm^–1^ (CHCl_3_) 3393, 3006, 2955, 2930, 2885, 1690, 1471, 1362, 1320, 1257, 1176, 1132, 1085 and 1062; ^1^H NMR (400 MHz, chloroform-d) *δ* 9.20 (1H, s, NH), 7.31 (1H, q, *J* 1.2, C6H), 6.28 (1H, t, *J* 6.7, C1′H), 4.450 (1H, dd, *J* 11.2, 3.0, C5′HH) 4.45–4.38 (1H, m, C4′H), 4.36 (1H, dd, *J* 11.2, 3.6, C5′HH), 4.05 (1H, app. q, *J* 3.6, C3′H), 3.06 (3H, s, SO_2_CH_3_), 2.28 (1H, ddd, *J* 13.6, 6.4, 3.9, C2′HH), 2.17 (1H, dt, *J* 13.6, 6.8, C2′HH), 1.93 (3H, d, *J* 1.3, CH_3_), 0.88 (9H, s, SiC(CH_3_)_3_), 0.09 (6H, s, Si(CH_3_)_2_); ^13^C NMR (101 MHz, chloroform-d) *δ* 163.9 (C4), 150.4 (C2), 135.6 (C6H), 111.7 (C5), 85.4 (C4′H, 84.3 (C1′H), 71.5 (C3′H), 68.5 (C6′H_2_), 40.6 (C2′H_2_), 37.8 (SO_2_CH_3_), 25.7 (SiC(CH_3_)_3_) 17.9, (SiC(CH_3_)_3_) 12.6 (CH_3_), –4.6 (SiCH_3_), –4.8 (SiCH_3_); HRMS (ESI) C_17_H_31_N_2_O_7_SSi (M + H) requires 435.1616; found 436.1624 and C_17_H_30_N_2_NaO_7_SSi (M + Na) requires 457.1435; found 457.1444.

### 5′-Azide-3′-*O-tert*-butyldimethylsilyl deoxythymidine (**10**)

A solution of 5′-*O*-mesyl-3′-*O-tert*-butyldimethylsilyl deoxythymidine (**9**) (1.29 g, 3.0 mmol) and sodium azide (580 mg, 9.0 mmol) in dry DMF (12 mL) under argon was heated to 100 °C for 14 h. The reaction was cooled to room temperature, diluted with water (100 mL) and extracted with diethyl ether (3 × 100 mL). The combined organic layers were washed with brine (3 × 75 mL), over MgSO_4_, and evaporated. The residue was purified by silica gel chromatography, eluting with petrol : diethyl ether (1 : 1 to 0 : 1) to afford the title compound (686 mg, 61%) as a white foam; *R*_f_ 0.18 (petrol : diethyl ether 1 : 1); [*α*]22D +80 (*c* 0.78, CHCl_3_); *ν*_max_/cm^–1^ (CHCl_3_) 3087, 3062, 3010, 2928, 2855, 2104, 1959, 1701, 1670, 1624, 1554, 1509, 1480, 1448, 1381, 1349, 1309, 1249, 1178, 1153; ^1^H NMR (500 MHz, chloroform-d) *δ* 8.95 (1H, s, NH), 7.31 (1H, q, *J* 1.2, C6H), 6.24 (1H, t, *J* 6.6, C1′H), 4.34 (1H, dt, *J* 7.0, 4.3, C3′H), 3.93 (1H, dt, *J* 4.4, 3.5, C4′H), 3.70 (1H, dd, *J* 13.3, 3.4, C5′HH), 3.49 (1H, dd, *J* 13.3, 3.6, C5′HH), 2.28 (1H, ddd, *J* 13.6, 6.6, 4.3, C2′HH), 2.16 (1H, dt, *J* 13.7, 6.9, C2′HH), 1.94 (3H, d, *J* 1.3, CH_3_), 0.88 (9H, s, SiC(CH_3_)_3_), 0.08 (6H, s, Si(CH_3_)_2_); ^13^C NMR (126 MHz, chloroform-d) *δ* 163.8 (C4), 150.4 (C2), 135.5 (C6H), 111.5 (C5), 84.9 (C4′H, and C1′H), 71.8 (C3′H), 51.9 (C6′H_2_), 40.8 (C2′H_2_), 25.8 (SiC(CH_3_)_3_), 18.04 (SiC(CH_3_)_3_), 12.8 (CH_3_), –4.54 (SiCH_3_), –4.77 (SiCH_3_); HRMS (ESI) C_16_H_28_N_5_O_4_Si (M + H) requires 382.1905; found 382.1910 and C_16_H_27_N_5_NaO_4_Si (M + Na) requires 404.1725; found 404.1737.

### 5′-Azide-3′-*O*H deoxythymidine (**4**)

To a stirred solution of 5′-azido-3′-*O-tert*-butyldimethylsilyl deoxythymidine (**10**) (151 mg, 400 μmol) in THF (2.0 mL) under an argon atmosphere, was added tetrabutylammonium fluoride trihydrate (189 mg, 600 μmol). The resulting solution was stirred at room temperature for 12 hours, and then the volatile organics were evaporated. The residue was purified by silica gel chromatography, eluting with EtOAc : MeOH (19 : 1) to afford the title compound (95 mg, 89%) as a white foam; *R*_f_ 0.20 (EtOAc); [*α*]22D +114 (*c* 1.0, CHCl_3_); *ν*_max_/cm^–1^ (CHCl_3_); 3390, 3009, 2956, 2105, 1690, 1471, 1438, 1262; ^1^H NMR (500 MHz, Methanol-d_4_) *δ* 7.54 (1H, q, *J* 1.2, C6H), 6.26 (1H, t, *J* 6.8, C1′H)), 4.34 (1H, dt, *J* 6.5, 4.1, C3′H), 3.96 1H, (dt, *J* 5.0, 3.8, C5′H), 3.63 (1H, dd, *J* 13.2, 3.7, C5′HH), 3.57 (1H, dd, *J* 13.2, 5.1, C5′HH), 2.31 (1H, dd, *J* 13.7, 6.6, C2′HH), 2.25 (1H, ddd, *J* 13.7, 6.6, 3.9, C2′HH), 1.89 (3H, d, *J* 1.3, CH_3_).^13^C NMR (126 MHz, Methanol-d_4_) *δ* 166.3 (C4), 152.3 (C2), 137.7 (C6H), 111.9 (C5), 86.4 (C4′H or C1′H) 86.3 (C4′H or C1′H), 72.5 (C3′H), 53.4 (C6′H_2_), 40.2 (C2′H_2_), 12.5 (CH_3_); HRMS (ESI) C_10_H_14_N_5_O_4_ (M + H) requires 268.1040; found 268.1044 and C_10_H_14_N_5_NaO_4_ (M + Na) requires 290.0859; found 290.0858.

### ^TL^Morpholino-3′-*O-tert*-butyl silyl dimer T-T (**11**)

To a microwave vial containing the morpholino thymidine (**3**) (290 mg, 500 μmol) and the azide thymidine (**10**) (190 mg, 500 μmol) in THF : ^*t*^BuOH : H_2_O (3 : 2 : 1 ratio, total volume 2 mL) was added copper iodide (47.0 mg, 250 μmol). The vial was sealed, stirred and irradiated in a Biotage microwave at 80 °C (approximately power of irritation 16 W) for 3½ h. After cooling to room temperature the vial was removed, and the solvents were removed *in vacuo* to afford a residue, which was purified by silica gel chromatography, eluting with DCM : MeOH (40 : 1 to 30 : 1) to afford the title compound (339 mg, 72%) as a colourless foam; *R*_f_ 0.21 (DCM : MeOH 25 : 1); [*α*]24D +66 (*c* 0.74, CHCl_3_); *ν*_max_/cm^–1^ (CHCl_3_) 3603, 3390, 3305, 3200, 2934, 2838, 2552, 1905, 1713, 1681, 1633, 1611, 1584, 1490, 1456; ^1^H NMR (400 MHz, chloroform-d) *δ* 9.49 (1H, s, NH), 9.38 (1H, s, NH), 7.61 (1H, s, C=CHN), 7.46–7.37 (2H, m, Ph), 7.35–7.15 (8H, m, 4 × Ar, C6H, 3 × Ph), 6.86–6.76 (4H, m, Ar), 6.68 (1H, d, *J* 1.4, C6H), 6.07 (1H, t, *J* 6.6, C1′H), 5.79 (1H, dd, *J* 9.7, 2.6, C1′H),4.72–4.56 (2H, m, NCH_2_C), 4.45 (1H, dt, *J* 7.0, 5.2, C3′H), 4.15–4.00 (2H, m, C4′H and C4′H), 3.80 (1H, d, *J* 13.9, OC5′HH) 3.78 (6H. s, OCH_3_), 3.68 (1H, d, *J* 13.9, OC5′HH), 3.26 (1H, dd, *J* 9.7, 5.3, NC5′HH), 3.09–2.97 (2H, m, NC5′HH and NCHH), 2.91 (1H, d, *J* 10.9, NCHH), 2.36–2.18 (2H, m, C2′H_2_), 2.10 (1H, t, 10.7, NCHH), 2.04 (1H, t, 10.7, NCHH), 1.92 (3H, d, *J* 1.2, CH_3_), 1.92 (3H, d, *J* 1.2, CH_3_), 0.89 (9H, s, SiC(CH_3_)_3_), 0.11 (3H, s, SiCH_3_), 0.08 (3H, s, SiCH_3_); ^13^C NMR (101 MHz, chloroform-d) *δ* 163.8 (2 × C4), 158.6 (2 × ^Ar^C), 150.3 (C2), 150.2 (C2), 144.8 (^Ar^C), 143.86 (C=) 136.4 (C6H), 135.9 (^Ar^C), 135.8 (^Ar^C), 135.62 (C6H), 130.2 (2 × ^Ar^CH), 130.14 (2 × ^Ar^CH), 128.23 (2 × ^Ph^CH), 127.9 (2 × ^Ph^CH), 126.9 (^Ph^CH), 124.7 (=CHN), 113.2 (4 × ^Ar^CH), 111.6 (C5), 110.9 (C5), 86.6 (Ar_3_CO), 86.2 (C1′H), 84.2 (C4′H), 79.8 (C1′H), 75.8 (C4′H), 72.0 (C3′H), 64.6 (NC5′H_2_), 55.9 (NCH_2_), 55.3 (2 × OCH_3_), 54.4 (NCH_2_), 52.8 (OC5′H_2_), 50.9 (NCH_2_C), 39.4 (C2′H_2_), 25.8 (SiC(CH_3_)_3_), 17.9 (SiC(CH_3_)_3_), 12.70 (2 × CH_3_), –4.5 (SiCH_3_), –4.6 (SiCH_3_); HRMS (ESI +ve) C_50_H_63_N_8_O_10_Si (M + H^+^) requires 963.4431, found 963.4436, and C_50_H_62_N_8_NaO_10_Si (M + Na^+^) requires 985.4250, found 985.4231.

### ^TL^Morpholino-3′-*O*H dimer T-T (**12**)

#### Method 1 – direct Cu^I^ catalysed cycloaddation with an unprotected azide

To a microwave vial containing the morpholino thymidine (**3**) (203 mg, 350 μmol) and the azide thymidine (**4**) (95 mg, 350 μmol) in THF : ^*t*^BuOH : H_2_O (3 : 2 : 1 ratio, total volume 1.75 mL) was added copper iodide (33.0 mg, 175 μmol). The vial was sealed, stirred and irradiated in a Biotage microwave at 80 °C (approximately power of irritation 16 W) for 3½ h. After cooling to room temperature the vial was removed, and the solvents were removed *in vacuo* to afford a residue, which was purified by silica gel chromatography, eluting with DCM : MeOH (12 : 1 to 10 : 1) to afford the title compound (223 mg, 75%) as a white foam; *R*_f_ 0.12 (DCM : MeOH 10 : 1); [*α*]24D –7.13 (*c* 1.0, CHCl_3_); *ν*_max_/cm^–1^ (ATR) 3390, 3008, 2961, 1690, 1608, 1509, 1490, 1456; ^1^H NMR (500 MHz, chloroform-d) *δ* 9.93 (1H, s, NH), 9.86 (1H, s, NH), 7.72 (1H, s, C=CHN), 7.48–7.41 (2H, m, Ph), 7.37–7.25 (7H, m, 4 × Ar, C6H, 2 × Ph), 7.26–7.18 (1H, m, Ph), 6.95 (1H, s, C6H), 6.89–6.80 (4H, m, Ar), 6.07 (1H, t, *J* 6.6, C1′H), 5.75 (1H, dd, *J* 8.8, C1′H), 4.74–4.68 (2H, m, NCH^2^C), 4.51 (1H, bs, C3′H), 4.39 (1H, bs, OH), 4.24 (1H, m, C4′H), 4.14–4.05 (1H, m, C4′H), 3.89 (1H, d, *J* 13.6, OC5′HH), 3.80 (6H, s, 2 × OCH_3_), 3.63 (1H, d, *J* 13.6, OC5′HH), 3.30 (1H, dd, *J* 9.7, 5.1, NC5′HH), 3.10 (1H, dd, *J* 9.7, 5.0, NC5′HH), 3.07–2.97 (2H, m, NCHH and NCHH), 2.33–2.26 (2H, m, C2′H_2_), 2.18 (1H, t, *J* 11.0, NCHH), 1.97 (1H, t, *J* 10.4, NCHH), 1.92 (3H, s CH_3_), 1.89 (3H, s, CH_3_); ^13^C NMR (126 MHz, chloroform-d) *δ* 164.0 (C4), 163.9 (C4), 158.5 (2 × ^Ar^C), 150.5 (C2), 150.4 (C2), 144.6 (C=), 143.8 (^Ar^C), 136.6 (C6H), 135.8 (^Ar^C), 135.7 (^Ar^C), 135.5 (C6H), 130.1 (2 × ^Ar^CH), 130.0 (2 × ^Ar^CH), 128.1 (2 × ^Ar^CH), 127.8 (2 × ^Ar^CH), 126.9 (^Ph^CH), 124.6 (=CHN), 113.1 (4 × ^Ar^CH), 111.3 (C5), 111.0 (C5), 86.52 (C1′H), 86.1 (Ar_3_CO), 83.9 (C4′H), 79.9 (C1′H), 75.7 (C4′H), 71.6 (C3′H), 64.3 (NC5′H_2_), 55.4 (NCH_2_), 55.2 (2 × OCH_3_), 54.9 (NCH_2_), 52.7 (OC5′H_2_), 51.4 (NCH_2_C), 38.9 (C2′H_2_), 12.6 (CH_3_), 12.5 (CH_3_); HRMS (ESI +ve) C_44_H_49_N_8_O_10_ (M + H^+^) requires 849.3566, found 849.3644.

#### Method 2; TBAF deprotection of TBS protected triazole

To a stirred solution of the ^TL^morpholino-3′-*O-tert*-butyl silyl dimer T-T (**11**) (339 mg, 358 μmol) in THF (2.0 mL) under an argon atmosphere, was added tetrabutylammonium fluoride trihydrate (141 mg, 447 μmol). The resulting solution was stirred at room temperature for 12 hours, then ammonium chloride (28 mg, 540 μmol) was added and stirred for 5 minutes. The resulting reaction mixture was dry loaded on to silica and purified by silica gel chromatography, eluting with DCM : MeOH (12 : 1 to 10 : 1) to afford the title compound (251 mg, 82%) as a white foam; *R*_f_ 0.12 (DCM : MeOH 10 : 1).

### ^TL^Morpholino phosphoramidite T-T (**13**)

To a stirred solution of the ^TL^Morpholino-3-*O*H dimer T-T (**12**) in DCM under an argon atmosphere at room temperature was added *N*,*N*-diisopropylethylamine (98 μL, 561 μmol) followed by 2-cyanoethyl *N*,*N*-diisopropylchlorophosphoramidite (59.0 mg, 266 μmol) dropwise over 1 minute, then stirred at room temperature for 24 h. The solvent was blown off with a stream of nitrogen gas, and the residue was purified by silica column chromatography, eluting with DCM : MeOH (20 : 1 to 15 : 1) to afford an analytical pure sample of the title compound (38 mg, 14%) as a white foam along with the bulk material (167 mg *ca.* 60%) containing small amounts of 2-cyanoethyl *N*,*N*-dipropylphosphonamidate as an off white foam; *R*_f_ 0.15 (DCM : MeOH 15 : 1); *ν*_max_/cm^–1^ (CHCl_3_) 3698, 3665, 3391, 3212, 2857, 2552, 2300, 2105, 2047, 1908, 1731, 1681, 1633, 1592, 1490 and 1455; ^1^H NMR (500 MHz, chloroform-d) *δ* 8.66 (1H, s, NH), 8.66 (1H, s, NH), 7.63 and 7.59 (1H, 2 × s, C=CHN), 7.48–7.41 (2H, m, Ph), 7.35–7.23 (7H, m, 4 × Ar, C6H, 2 × Ph), 7.23–7.17 (1H, m, Ph), 6.87–6.77 (5H, m, Ar and C6H), 6.12 (1H, m, C1′H), 5.77 (1H, m, C1′H), 4.82–4.54 (3H, m, NCH_2_C and C3′H), 4.34–4.22 (1H, m, C4′H), 4.07–4.02 (1H, m, C4′H), 3.90–3.85 (1H, m, OCHH), 3.84–3.79 (1H, m, OC5′HH), 3.79 (6H, s, 2 × OCH_3_), 3.84–3.68 (1H, m, OCHH), 3.69–3.54 (3H, m, OC5′HH and 2 × NCH), 3.26 (1H, dd, *J* 9.7, 5.1, NC5′HH), 3.08–2.96 ((2H, m, NC5′HH and NCHH), 2.92 (1H, m, NCHH), 2.75–2.68 (1H, m, CHHCN), 2.71–2.59 (1H, m, CHHCN), 2.51–2.22 (2H, m, C2′H_2_), 2.09 (1H, t, *J* 11.0, NCHH), 2.07–1.97 (1H, m, NCHH), 1.92 (3H, s CH_3_), 1.90 (3H, s, CH_3_), 1.20 (12H, m, NCH(CH_3_)_2_); ^13^C NMR (500 MHz, chloroform-d) due to the presence of a diastereomeric mixture at the phosphorus(iii) centre and coupling from phosphorus the ^13^C NMR could not be unambiguously assigned but the spectra are included in this ESI;† ^31^P NMR (162 MHz, CDCl_3_) *δ* 149.2; HRMS (ESI +ve) C_53_H_66_N_10_O_11_P (M + H^+^) requires 1049.4645, found 1049.4653 and C_53_H_65_N_10_NaO_11_P (M + Na^+^) requires 1071.4464, found 1071.4489.

### 3′-*O*-Propargyl thymidine (**15**)

To a stirred solution of 5′-*O*-DMT thymidine (1.40 g, 2.55 mmol) in THF (25 mL) at 0 °C was added sodium hydride (257 mg, 6.4 mmol) in small portions over 5 min. The resulting solution was stirred at 0 °C for 30 min, then at room temp for 1 h. The solution was cooled to 0 °C and propargyl bromide (285 μL, 3.18 mmol) was added. The solution was stirred at 0 °C for 30 min, then at room temp for 5 h. The reaction was quenched by the addition of water (1 mL), and the volatile organic were removed by evaporation. The residue was partitioned between water (25 mL) and DCM (25 mL), the aquous layer was extracted with DCM (3 × 25 mL). The combined organic layers were washed with brine (2 × 25 mL), dried over MgSO_4_, and evaporated *in vacuo* to afford a residue, which was purified by silica gel chromatography, eluting with DCM : MeOH (38 : 1 to 19 : 1) to afford the title compound (1.15 g, 77%) as a white foam; *R*_f_ 0.4 (DCM : MeOH 19 : 1); [*α*]24D 33.0 (*c* 1.0, CHCl_3_); *ν*_max_/cm^–1^ (ATR); 3385, 3190, 2950, 1930, 1695, 1631, 1611, 1491; ^1^H NMR (400 MHz, chloroform-d) *δ* 8.75 (1H, br s, N*H*), 7.63 (1H, s, C6*H*), 7.46–7.41 (2H, m, Ar), 7.36–7.25 (7H, m, Ar), 6.89–6.85 (4H, m, Ar), 6.36 (1H, d, *J* 8.0 and 5.7, C1′H), 4.53 (1H, dt, *J* 5.3 and 2.2, C4′H), 4.21 (1H, dd, *J* 15.9 and 2.3, OCHHCCH), 4.21–4.16 (1H, m, C5′H), 4.16 (1H, dd, *J* 15.9 and 2.3, OCHHCCH), 3.82 (6H, s, 2 × OCH_3_), 3.50 (1H, dd, *J* 10.6 and 3.0, C6′HH), 3.38 (1H, dd, *J* 10.6 and 2.7, C6′HH), 2.54 (1H, ddd, *J* 13.9, 5.7 and 2.2, C2′HH), 2.44 (1H, t, *J* 2.3, CCH), 2.26 (1H, ddd, *J* 13.9, 8.0 and 6.3, C2′HH), 1.53 (3H, s, CH_3_); ^13^C NMR (101 MHz, chloroform-*d*) *δ* 163.8 (C4), 158.8 (2 × C), 150.4 (C2), 144.5 (C), 135.6 (C6H), 135.5 (2 × C), 130.2 (4 × CH), 128.2 (2 × CH), 128.1 (2 × CH), 127.3 (CH), 113.4 (4 × ArCH), 111.3 (C5H), 87.1 (C), 84.91 (C1′H), 84.0 (C5′H), 79.2 (C), 78.6 (C4′H), 75.2 (CH), 63.6 (C5′H_2_), 56.7(CH_2_), 55.4 (2 × OCH_3_), 37.9 (C2′H_2_), 12.0 (CH_3_); HRMS (ESI +ve) C_34_H_34_N_2_NaO_7_ (M + Na^+^) requires 605.2258, found 605.2247.

### Triazole-T-T dimer (**16**)

To a microwave vial containing the 3′-*O*-propargyl thymidine (**15**) (1.164 g, 2.0 *m*mol) and the azide thymidine (**4**) (534 mg, 2.0 *m*mol) in THF : ^*t*^BuOH : H_2_O (3 : 2 : 1 ratio, total volume 12 mL) was added copper iodide (188 mg, 1.0 mol). The vial was sealed, stirred and irradiated in a Biotage microwave at 80 °C (approximately power of irritation 16 W) for 3½ h. After cooling to room temperature the vial was removed, and the solvents were removed *in vacuo* to afford a residue, which was purified by silica gel chromatography, eluting with DCM : MeOH (20 : 1 to 10 : 1) to afford the title compound (1.47 g, 87%) as a white foam; *R*_f_ 0.35 (DCM : MeOH 10 : 1); [*α*]24D 5.2 (*c* 1.0, CHCl_3_); *ν*_max_/cm^–1^ (ATR) 3392, 3010, 2963, 1691, 1604, 1493, 1460; ^1^H NMR (400 MHz, DMSO-*d*_6_) *δ* 11.35 (1H, br s, N*H*), 11.31 (1H, br s, N*H*), 8.08 (1H, s, triazole-CH), 7.51 (1H, s, C6H), 7.40–7.29 (4H, m, Ar), 7.33 (1H, s, C6H), 7.28–7.22 (5H, m, Ar), 6.90 (4H, d, *J* 8.7 Hz, Ar), 6.16 (1H, app t, *J* 6.4 Hz C1H), 6.14 (1H, app t, *J* 6.4 Hz C1H), 5.50 (1H, d, *J* 4.4, OH), 4.70 (1H, dd, *J* 14.3 and 4.4, NC5′HH), 4.59 (1H, dd, *J* 14.3 and 7.7, NC5′HH), 4.60–4.52 (2H, m, OCH_2_C), 4.45–4.37 (1H, m, C3′H), 4.31–4.24 (1H, m, C3′H), 4.10–4.02 (2H, m, C4′H and C4′H), 3.74 (6H, s, 2 × OCH_3_), 3.26 (1H, dd, *J* 10.5, 3.8 Hz, OC5′HH), 3.17 (1H, dd, *J* 10.5, 3.2 Hz, OC5′HH), 2.43–2.25 (2H, m, C2′HH), 2.25–2.04 (2H, m, C2′HH), 1.78 (3H, s, CH_3_), 1.43 (3H, s, CH_3_); ^13^C NMR (101 MHz, DMSO) *δ* 163.6 (2 × C4), 158.17 (2 × C), 150.37 (2 × C2), 144.6 (C), 143.7 (C), 136.0 (C6), 135.5 (C6), 135.4 (C), 135.1 (C), 129.7 (4 × CH), 127.9 (2 × CH), 127.6 (2 × CH), 126.8 (CH), 124.7 (triazole-CH), 113.3 (4 × ArCH), 109.8 (C5H), 109.7 (C5H), 86.04 (C), 84.02 (CH), 83.93 (CH), 83.78 (CH), 82.87 (CH), 78.75 (CH), 70.72 (CH), 63.77 (CH2), 61.77 (CH2), 55.05 (2 × OCH3), 51.17 (CH2), 37.9 (C2′H_2_), 36.5 (C2′H_2_), 12.1 (CH_3_), 11.9 (CH_3_); HRMS (ESI +ve) C_44_H_47_N_7_NaO_11_ (M + Na^+^) requires 872.3226, found 872.3215.

### Triazole-T-T dimer phosphoramidite (**17**)

To a stirred solution of the triazole-T-T dimer (**16**) (493 mg, 0.58 mmol) in DCM (1.2 mL) under an argon atmosphere at room temperature was added *N*,*N*-diisopropylethylamine (182 μL, 1.05 mmol) followed by 2-cyanoethyl *N*,*N*-diisopropylchlorophosphoramidite (125 mg, 0.58 mmol) dropwise over 1 minute, then stirred at room temperature for 24 h. The solvent was blown off with a stream of nitrogen gas, and the residue was purified by silica column chromatography, eluting with DCM : MeOH (20 : 1 to 15 : 1) to afford the title compound (391 mg, *ca.* 64%) containing 2-cyanoethyl *N*,*N*-diisopropylphosphonamidate as an off white foam; *R*_f_ 0.21 (DCM : MeOH 15 : 1); *ν*_max_/cm^–1^ (ATR) 3700, 3669, 3384, 3219, 2860, 2550, 2310, 2109, 1911, 1733, 1686, 1495 and 1459; ^1^H NMR (500 MHz, chloroform-d) *δ* 9.20 (2H, br s, N*H*), 7.69 and 7.65 (1H, 2 × s, triazole-CH), 7.60 (1H, s, C6H), 7.42–7.37 (2H, m, Ar), 7.37–7.21 (7H, m, Ar), 6.90–6.83 (4H, m, Ar), 6.78–6.73 (1H, 2 × s, C6H), 6.38–6.32 (1H, m, C1H), 6.21–6.13 (1H, m, C1H), 4.78–4.57 (5H, m, 2 × CH_2_ and OCH), 4.40–4.11 (3H, m, 3 × CH), 3.99–3.70 (2H, m, CH_2_), 3.81 (6H, s, 2 × OCH_3_), 3.68–3.45 (3H, m, 2 × CH and C*H*H), 3.38–3.32 (1H, m, CH*H*), 2.80–2.75 (1H, m, CHHCN) 2.70–2.65 (1H, m, CHHCN), 2.57–2.20 (4H, m, C2′HH and C2′HH), 1.90–1.87 (3H, m, CH_3_), 1.47 (3H, s, CH_3_); 1.26–1.18 (12H, m, NCH(CH_3_)_2_); *δ* ^13^C NMR (500 MHz, chloroform-d) due to the presence of a diastereomeric mixture at the phosphorus(iii) centre and coupling from phosphorus the ^13^C NMR could not be unambiguously assigned but the spectra are included in this ESI;† ^31^P NMR (162 MHz, CDCl_3_) *δ* 149.1; HRMS (ESI +ve) C_53_H_65_N_9_O_12_P (M + H^+^) requires 1050.4485, found 1050.4464.

## Conclusions

We have shown that the CuAAC reaction can be used to synthesise a new DNA mimic containing a triazole-linked morpholino (^TL^MO) internucleotide modification. Phosphoramidite reagents **13** and **17** were synthesised and their compatibility with automated solid phase synthesis was demonstrated. UV melting studies showed that incorporation of the ^TL^MO modification provided an improved Tm value for binding to RNA when compared to the previously reported triazole-containing oligomers. Structural characterisation, and biological evaluation of the ^TL^MO-modified oligomers is underway and the results of this work will be reported in due course.
