# Supplementary material for: Synthesis of triazole-linked morpholino oligonucleotides via CuI catalysed cycloaddition
Source: Org Biomol Chem. 2016 Feb 16;14(11):3112–9. doi: 10.1039/c6ob00007j (PMC5047124; doi:10.1039/c6ob00007j)

Electronic Supplementary Material (ESI) for Organic & Biomolecular Chemistry.
This journal is © The Royal Society of Chemistry 2016

ELECTRONIC SUPPORTING INFORMATION

Synthesis of Triazole-Linked Morpholino Oligonucleotides *via* Cu^{I} Catalysed Cycloaddition

Matthew J. Palframan,[‡] Rima D. Alharthy, [†‡] Paulina Powalowska and Christopher J. Hayes*

School of Chemistry, University of Nottingham, University Park, Nottingham, NG7 2RD, UK. Fax: +44(0)115 951 3564; Tel: +44(0)115 951 3045; E-mail: chris.hayes@nottingham.ac.uk

[†] Present address: Department of Chemistry, Faculty of Science, King Abdulaziz University, Jeddah, Saudi Arabia. Tel: +966(12)2577246; E-mail: iaaalharte@kau.edu.sa

[‡] These authors contributed equally to this work.

1. Chemistry General details:

NMR spectra were recorded using either a Bruker DPX-400 (400 MHz), Bruker AV-400 (400 MHz), Bruker AV3-400 (400 MHz) or Bruker DRX 500 (500 MHz) spectrometer at ambient temperature, with samples being prepared as dilute solutions in the stated solvent. Chemical shifts are quoted in parts per million (ppm) downfield of tetramethylsilane, with the spectra being referenced to residual solvent. The multiplicity of each signal is designated by the following abbreviations: s (singlet), d (doublet), t (triplet), q (quartet), b (broad), m (multiplet) and app (apparent), and all coupling constants (*J*) are quoted in Hertz. Assignments were made on the basis of chemical shift, COSY, HMBC, HSQC and/or DEPT experiments. Mass spectra were recorded on a Bruker MicroTOF system, using electrospray (ESI) techniques.

Flash chromatography was carried on Merck silica gel 60 (230–400 mesh ASTM) , and all of the solvents used were of analytical grade; petroleum ether refers to light petroleum, bp 40–60 °C. Reactions were monitored by thin layer chromatography (TLC) using aluminium plates precoated with Merck silica gel 60F254; they were visualised using ultraviolet light (λ_{max} = 254 nm), and then stained using acid cerium molybdate solution or basic potassium permanganate solution.

Unless stated otherwise, reactions requiring anhydrous conditions were conducted in an inert atmosphere of argon in flame-dried or oven-dried apparatus. Dry organic solvents were routinely stored under a nitrogen or argon atmosphere. Dry and degassed Toluene, THF and MeCN were obtained by passing through anhydrous alumina columns using an Innovative Technology Inc. PS-400-7 solvent purification system. Dry DCM was distilled from CaH_2. All solvents were removed by concentration *in vacuo* at approx. 20 mm Hg using a Büchi rotary evaporator. Traces of solvent were then removed at approx. 0.2 mm Hg on a vacuum line

2. General procedures for oligonucleotides synthesis and analysis

Columns (SynBaseTM CPG), standard DNA and RNA-phosphoramidites and reagents for the synthesizer were purchased from Link Technologies Ltd (Scotland, UK). MeNH2 solution (33 wt.% in ethanol) was obtained from Fluka, NEt_3.3HF, N-methylpyrrolidinone (NMP) and 3-hydroxypicolinic acid (HPA) were purchased from Aldrich, illustra NapTM5 columns were obtained from GE Healthcare Europe GmbH and Oligonucleotide Purification Cartridges (OPCTM) were purchased from Applied Biosystems UK. Water used for oligonucleotides analysis and purification was either purified by Milli-Q ultra pure water system (Millipore) or commercial nuclease-free water. MeCN HPLC grade was used for oligos synthesis and purification.

2.1. Synthesis of oligonucleotides

Oligonucleotides were synthesised on ABI 3400 DNA synthesiser (0.2 μmol scale) following standard solid-phase chemistry. The following standard solutions were used;

- Deblock Mix (3% trichloroacetic acid in dichloromethane)
- BTT Activator (0.3M 5-benzylthio-1-H-tetrazole in acetonitrile)
- Oxidiser (0.02M iodine in THF/pyridine/water = 7:2:1)
- Cap Mix A (THF/pyridine/acetic anhydride = 8:1:1)

- Cap Mix B (10% methylimidazole in THF)

2.1.1 Synthesis and post-synthesis treatment of DNA oligonucleotides:

Unmodified parts of DNA oligonucleotides were synthesized using the standard (0.2 μmol scale protocol provided by the manufacturer. Modified dinucleotides were coupled with a increased coupling time of 600 s and using 4 coupling cycles.

Once synthesised, the DNA oligonucleotides were transferred to DNase free falcons and cleaved manually from the resin and by adding 33% ammonia solution (2 mL). The falcons were placed in Aluminium heating block and kept at 60 °C for 18 h for the purpose of the deprotection of cyanoethyl and removal of base-protecting groups.

The oligonucleotides were purified using the standard protocol supplied with OPC® (Applied Biosystems, Foster City, CA, USA). Stock solutions used in purification protocol were: MeCN HPLC Grade, TEAA (2 M), NH_4OH (1.5 M), 3% TFA, Milli-Q water and 20% MeCN. Oligos samples were diluted with Milli-Q water (2 mL) prior to OPC® and loaded twice on the cartridge. Detritylation occurred using 3% TFA, DMTr-off samples were eluted upon addition of 20% MeCN and freeze-dried prior to further purification / analysis.

2.1.2 Synthesis and post-synthesis treatment of RNA oligonucleotides:

RNA oligonucleotides were synthesized using 0.2 µM scale protocol with a 600 s coupling step for each nucleotide. The post-synthesis treatment can be categorized into the following steps:

- Cleavage and deprotection of cyanoethyl and base protecting groups:

  The polymer-bound oligoribonucleotide was transferred from the synthesis column to a DNase free falcons and suspended in MeNH2 solution (1 mL). The mixture was heated to 65C for 10 min, cooled to room temperature (water/ice bath) and centrifuged for 1 min (10 000 g). The

supernatant was separated from the CPG beads, the beads were washed with RNase free water (2 x 0.25 mL), all supernatants were combined and then freeze dried.

- Deprotection of 2′-O-TBDMS groups and Detritylation:

The oligoribonucleotide were resuspended in anhydrous NEt_3.3HF/NEt_3/NMP solution (250 µL of a solution of 1.5 mL NMP, 750 µL NEt_3 and 1.0 mL NEt_3.3HF), heated to 65C for 1.5 h, cooled to room temperature and quenched with 3M NaOAc solution (25 µL).

- Butanol precipitation:

n-BuOH (1 mL) was added to the mixture, which was then thoroughly mixed, cooled to -78 °C for 2 h to encourage further precipitation and centrifuged for 30 min (4 °C, 13 000 g). The supernatant was removed, the pellet washed with 70% EtOH (2 × 500 µL) and then freeze dried.

2.1.3 Oligonucleotides desalting and determination of the concentrations

The dry RNA and DNA oligonucleotide precipitate was dissolved in RNase free water (1 mL) and desalted using a Nap™-5 column following the standard protocol supplied with the Nap™-5 column. The resulting solution were stored in the freezer and used as stock solutions for mass spec and Tm analysis. Determination of the concentrations of oligos was carried out using a Thermo Scientific NanoDrop™ 1000 Spectrophotometer.

Identifier	Sequence 5′-3′	Stock concentrations
**DNA Target 18**	**GCTGCAAACGTCG**	110 µM
**RNA Target 19**	**GCUGCAAACGUCG**	65 µM
**Unmodified DNA 20**	**CGACGTTTGCAGC**	135 µM
**^{TL}MO 21**	**CGACGT*TTGCAGC**	63 µM
**^{TL}DNA 22**	**CGACGT^TTGCAGC**	105 µM

2.4. HPLC analysis of oligos.

DMTr-off oligos purity was assessed by analytical HPLC using an Agilent 1100 series HPLC and a Phenomenex Clarity 3 µ Oligo-RP 50 × 4.6 mm coloumn (Macclesfield, UK) with a flow rate of 1 mL/min following the elution gradient below

Time (min)	Buffer A %	Buffer B %
0	95	5
5	95	5
20	80	20
30	80	20
32	95	5
35	95	5

Table S1. Elution method of HPLC for the purification of oligos

Buffer A: TEAA (100 mM in Water)

Buffer B: MeCN HPLC grade.

Data was collected and analysed using Agilent Technologies, Chemstation for LC software.

Identifier	Sequence 5′-3′	Retention time (min)
**DNA Target 18**	**GCTGCAAACGTCG**	7.76
**RNA Target 19**	**GCUGCAAACGUCG**	17.08
**Unmodified DNA 20**	**CGACGTTTGCAGC**	7.86
**^{TL}MO 21**	**CGACGT*TTGCAGC**	8.10
**^{TL}DNA 22**	**CGACGT^TTGCAGC**	8.32

Table S2 HPLC Retention times

2.5. Mass Spec analysis of oligonucleotides.

Masses of oligomers were obtained on a Thermo Scientific: LTQ FT Ultra Mass Spectrometer in negative mode. Mass ions were found for the M-6H adducts.

Identifier	Sequence 5′-3′	MW	
		Calculated	Found
**DNA Target 18**	**GCTGCAAACGTCG**	3953.55	3953.73
**RNA Target 19**	**GCUGCAAACGUCG**	4133.49	4133.63
**Unmodified DNA 20**	**CGACGTTTGCAGC**	3944.53	3944.72
**^{TL}MO 21**	**CGACGT*TTGCAGC**	3944.64	3944.80
**^{TL}DNA 22**	**CGACGT^TTGCAGC**	3945.63	3945.78

Table S3. Mass spec data

2.6. UV-Melting experiments

The hybrids were formed at final concentration of 3 μM by dissolving the lyophilised oligonucleotides in 10 mM phosphate buffer with 200 mM NaCl at the reported pH (7.0). The samples were annealed by placing the tubes in an aluminium heating block at 90 °C for 10 min, the samples were remove from the heating block and allow to cool to room temperature (approx. 45 min). The samples were stored at 4 °C prior to Tm analysis. The melting curve of the duplex was obtained by following the temperature dependencies of the absorption at 260 nm in a micro Tm cell with a 1 cm path length. The temperature was varied between 20 and 85 °C at 1 °C/min, both heating and cooling data were recorded.

The data was normalised to % denaturation following equation 4 and plotted against temperature to obtain the melt curves.[15a,b]

$$\% \text{ Denaturation} = (A^0 - A^i / A^{max} - A^i) \text{x} 100 \qquad \textbf{equation (4)}$$

Where Abs^o: observed absorbance, Abs^i: initial absorbance at 25 °C and Abs^{max}: maximum absorbance at 85 °C. The values were combined to obtain a melting curve and the Tm was determined as the midpoint of the plot between the two states of the system.

3. HPLC chromatograms of oligonucleotides

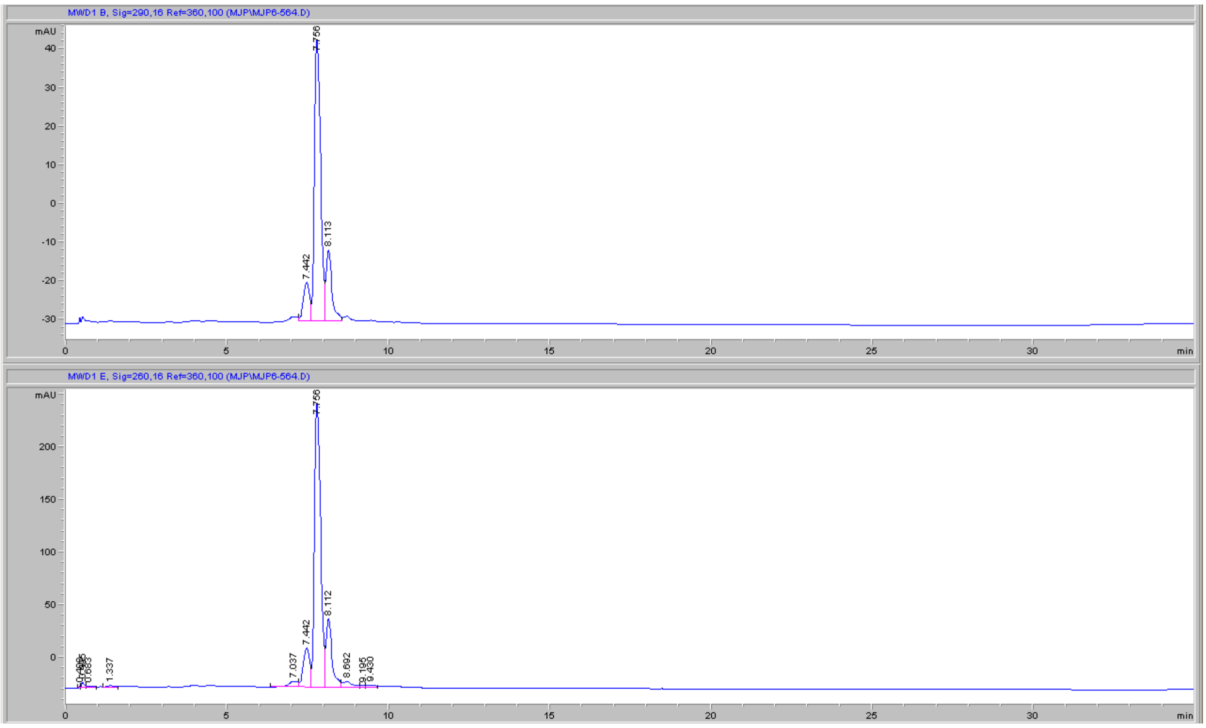

HPLC chromatogram of **DNA Target 18 (GCTGCAAACGTCG)**

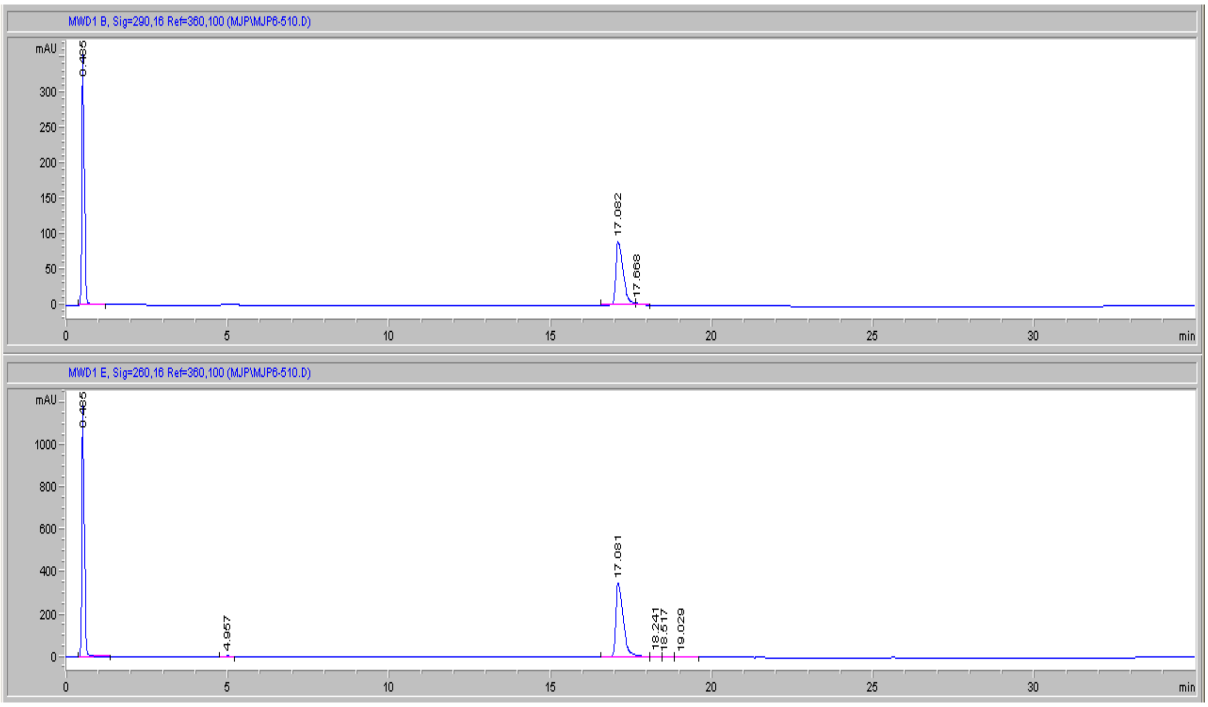

HPLC chromatogram of **RNA Target 19 (GCUGCAAACGUCG)**

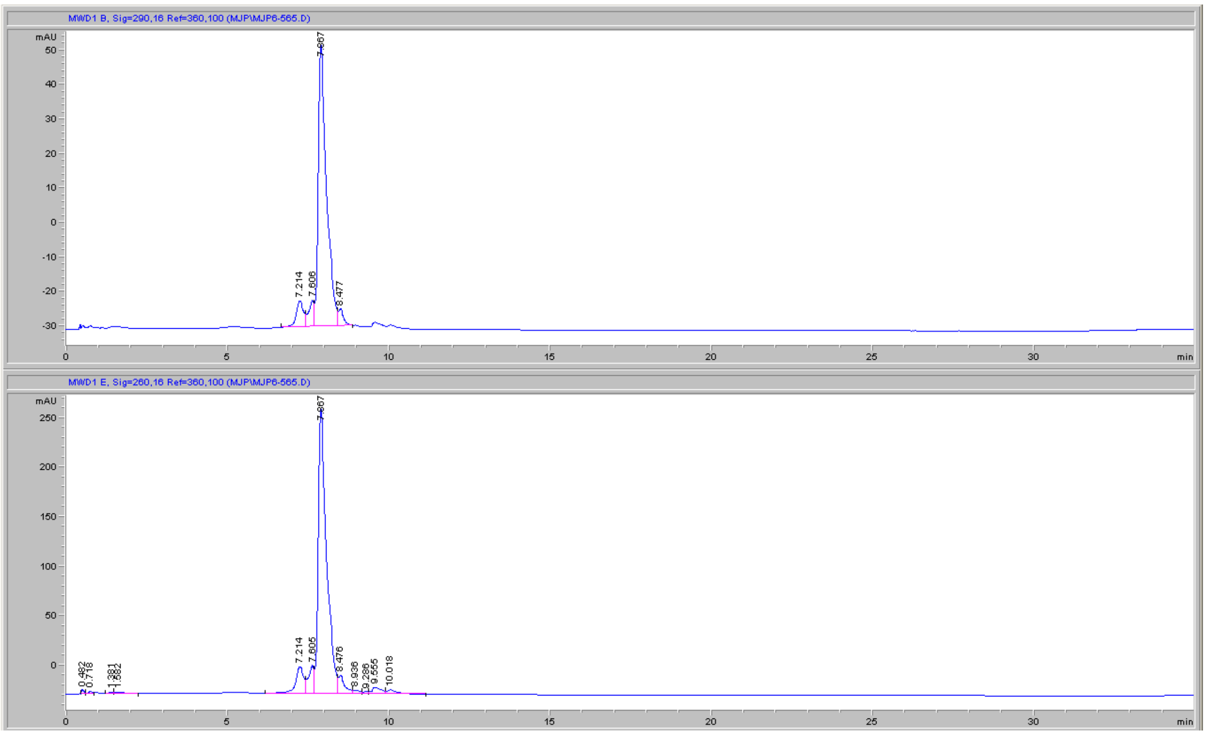

HPLC chromatogram of **Unmodified DNA 20** (**CGACGTTTGCAGC**)

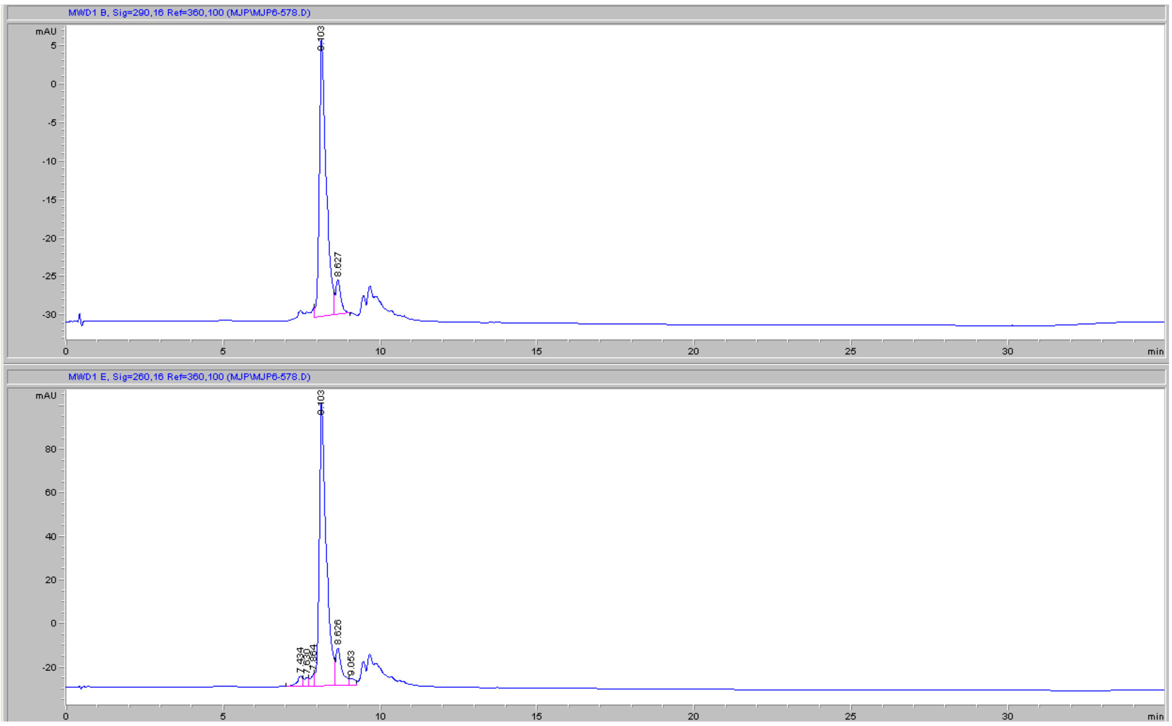

HPLC chromatogram of ^{TL}**MO 21 (CGACGT*TTGCAGC)**

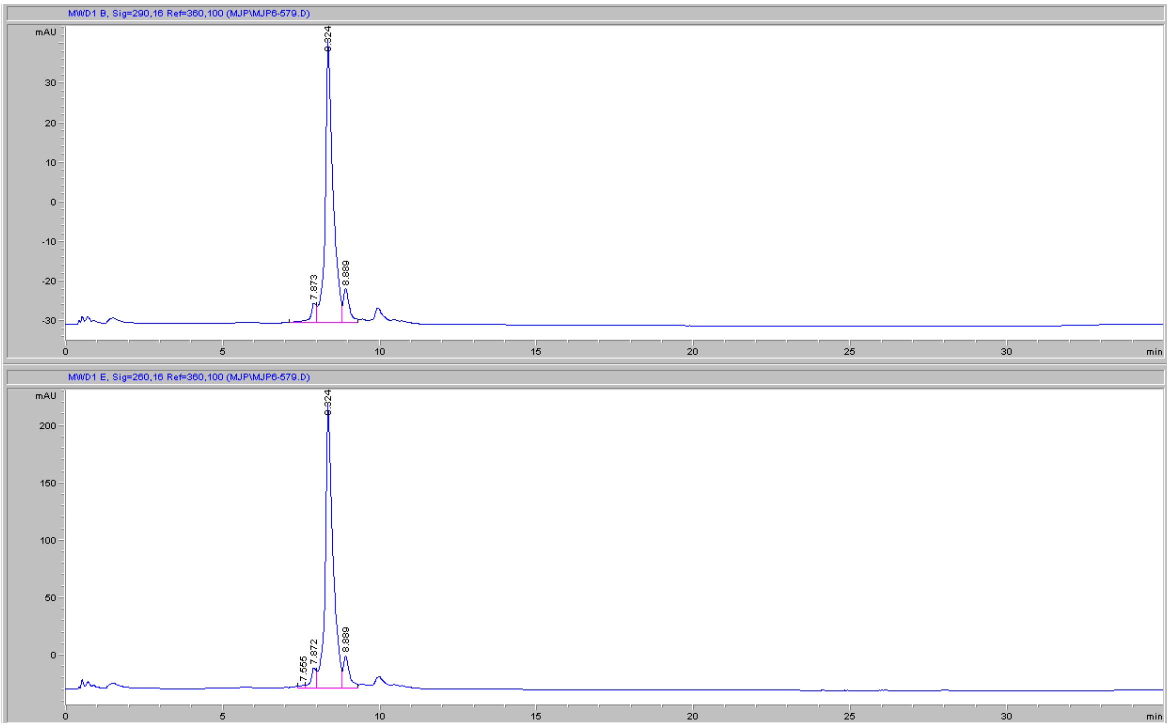

HPLC chromatogram of ^{TL}**DNA 22** (**CGACGT^TTGCAGC**)

4. Mass Spectra of oligonucleotides

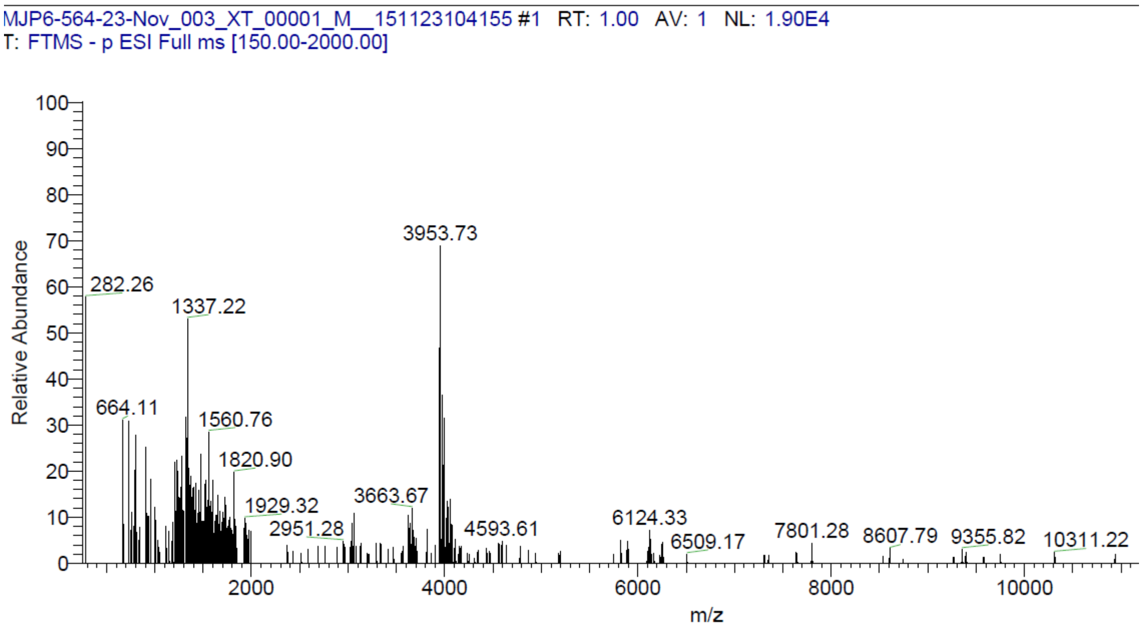

DNA Target 18 (GCTGCAAACGTCG) – found 3953.73; (M – $6H^{+}$) requires 3953.55

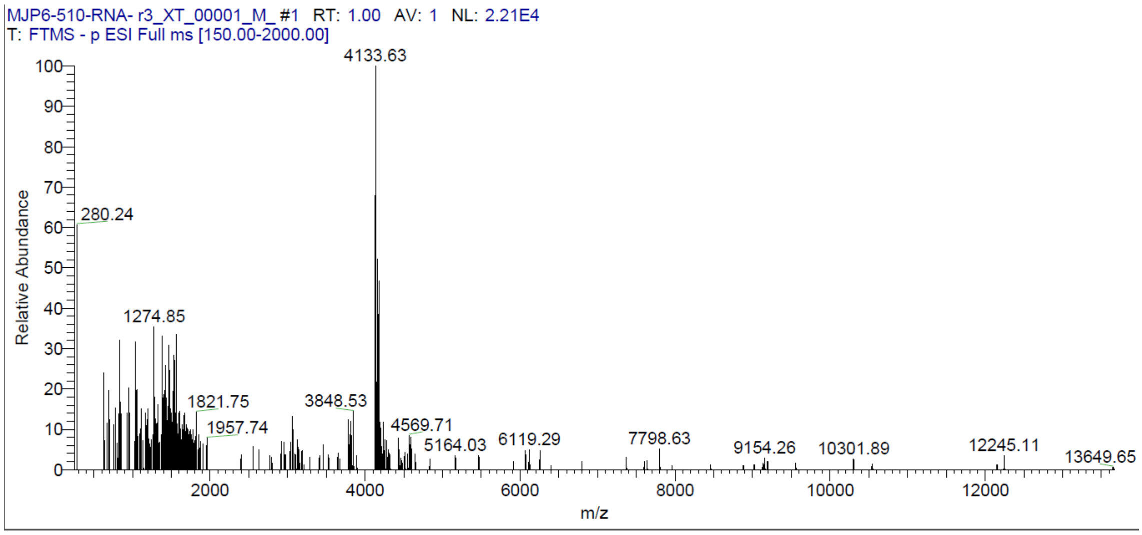

RNA Target 19 (GCUGCAAACGUCG) – found 4133.63; (M – $6H^{+}$) requires 4133.49

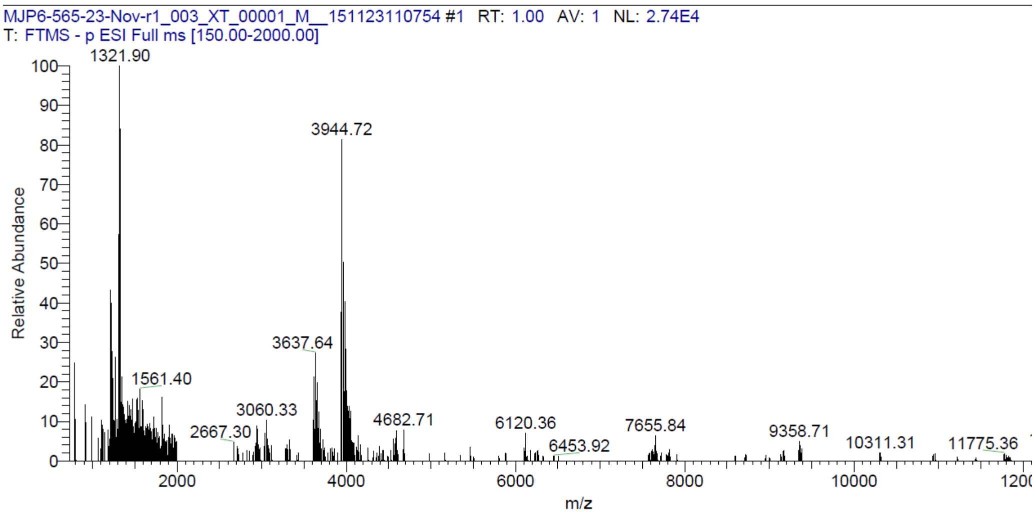

Unmodified DNA 20 (**CGACGTTTGCAGC**) – found 3944.72; (M – 6H^{+}) requires 3944.53

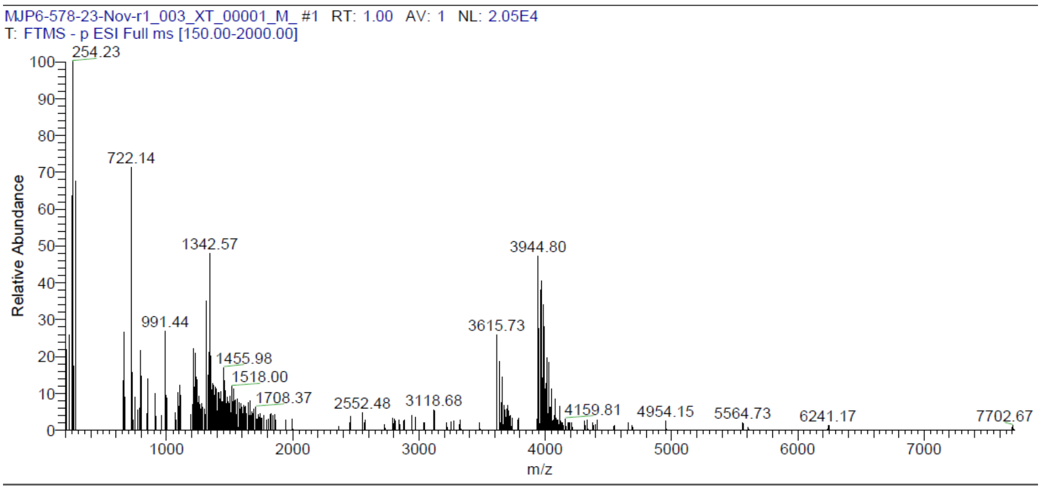

^{TL}**MO 21 (CGACGT*TTGCAGC)** – found 3944.80; (M – 6H^{+}) requires 3944.64

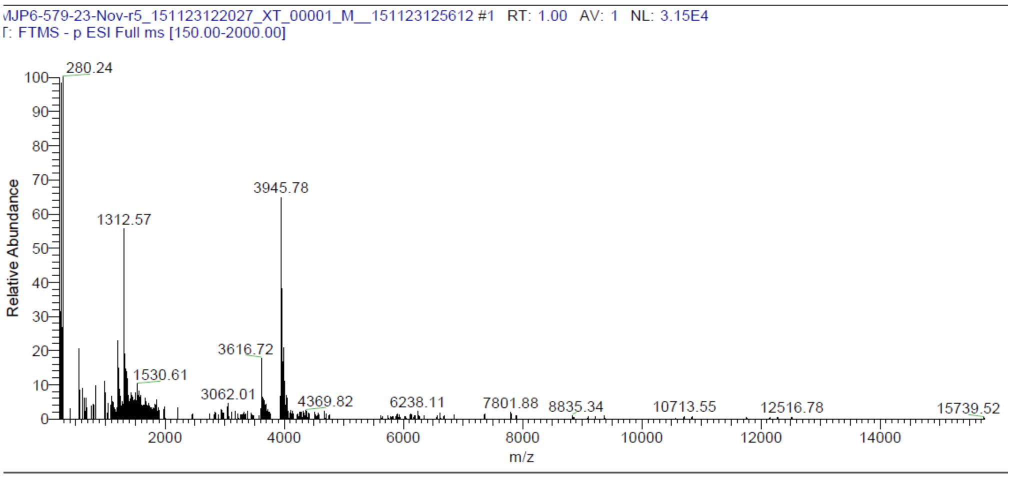

^{TL}**DNA 22 (CGACGT∧TTGCAGC)** – found 3945.78; (M – 6H^{+}) requires 3945.63

Tm data for RNA duplex at pH 7.0

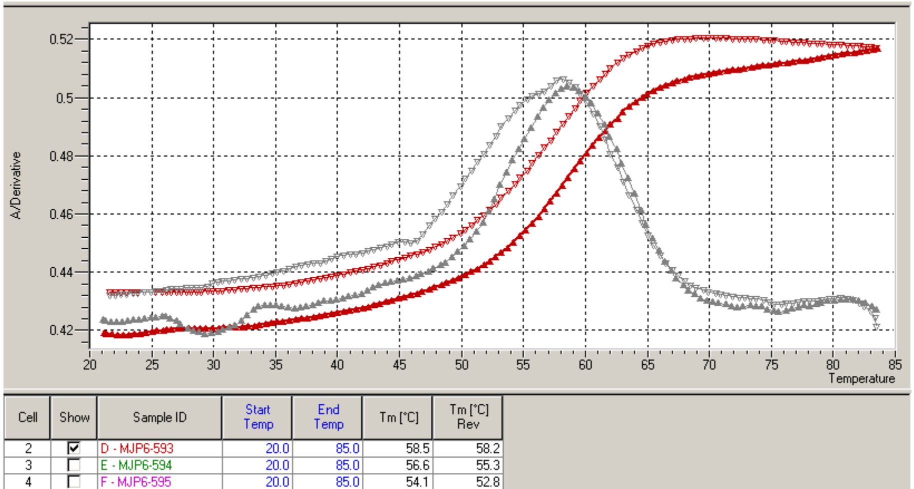

Cell	Show	Sample ID	Start Temp	End Temp	Tm [°C]	Tm [°C] Rev
2	☑	D - MJP6-593	20.0	85.0	58.5	58.2
3	☐	E - MJP6-594	20.0	85.0	56.6	55.3
4	☐	F - MJP6-595	20.0	85.0	54.1	52.8

Duplex of RNA **19** and unmodified DNA **20**

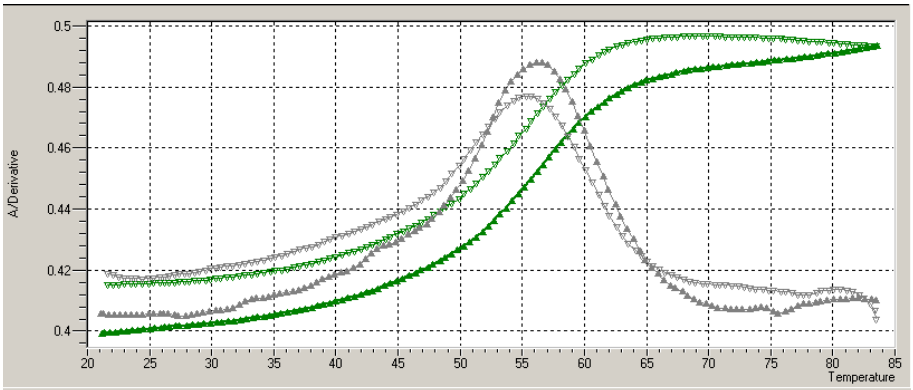

Cell	Show	Sample ID	Start Temp	End Temp	Tm [°C]	Tm [°C] Rev
2	☐	D - MJP6-593	20.0	85.0	58.5	58.2
3	☑	E - MJP6-594	20.0	85.0	56.6	55.3
4	☐	F - MJP6-595	20.0	85.0	54.1	52.8

Duplex of RNA **19** and ^{TL}MO **21**

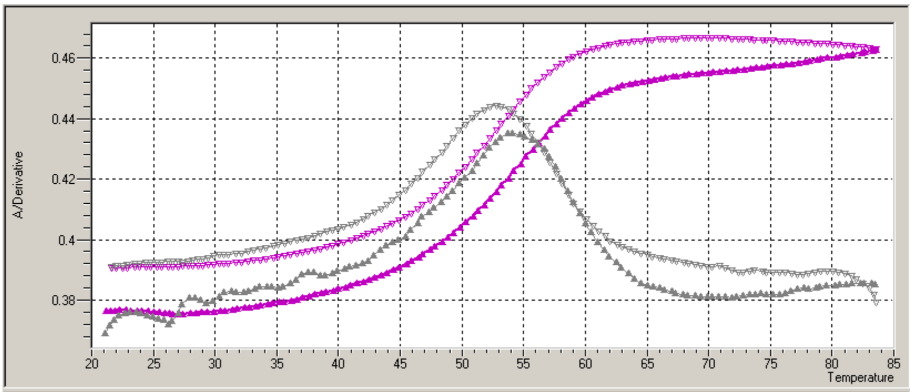

Cell	Show	Sample ID	Start Temp	End Temp	Tm [°C]	Tm [°C] Rev
2	☐	D - MJP6-593	20.0	85.0	58.5	58.2
3	☐	E - MJP6-594	20.0	85.0	56.6	55.3
4	☑	F - MJP6-595	20.0	85.0	54.1	52.8

Duplex of RNA **19** and ^{TL}DNA **22**

Tm data for DNA duplex at pH 7.0

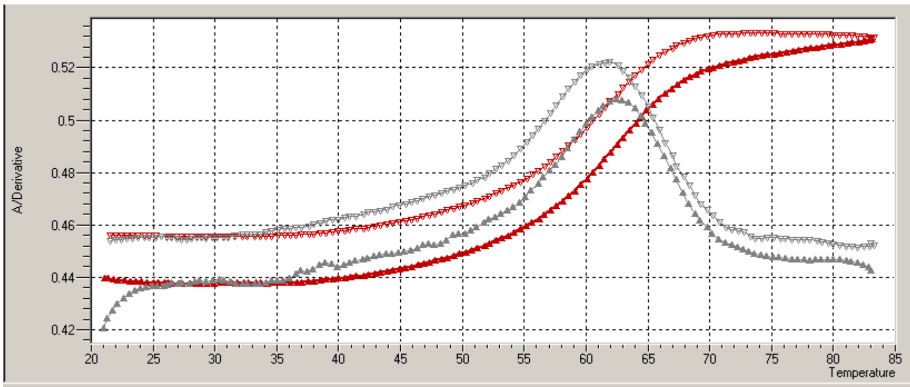

Cell	Show	Sample ID	Start Temp	End Temp	Tm [°C]	Tm [°C] Rev
2	☑	A - MJP6-590	20.0	85.0	62.4	61.9
3	☐	B - MJP6-591	20.0	85.0	56.1	54.7
4	☐	C - MJP6-592	20.0	85.0	55.1	54.1

Duplex of DNA **18** and unmodified DNA **20**

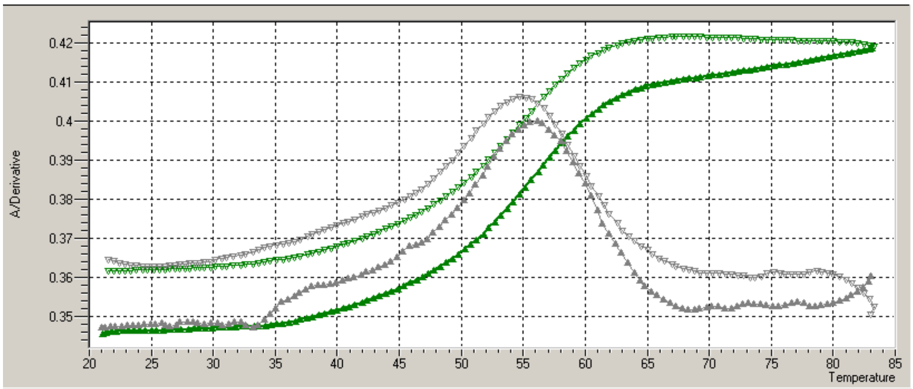

Cell	Show	Sample ID	Start Temp	End Temp	Tm [°C]	Tm [°C] Rev
2	☐	A - MJP6-590	20.0	85.0	62.4	61.9
3	☑	B - MJP6-591	20.0	85.0	56.1	54.7
4	☐	C - MJP6-592	20.0	85.0	55.1	54.1

Duplex of DNA **18** and ^{TL}MO DNA **21**

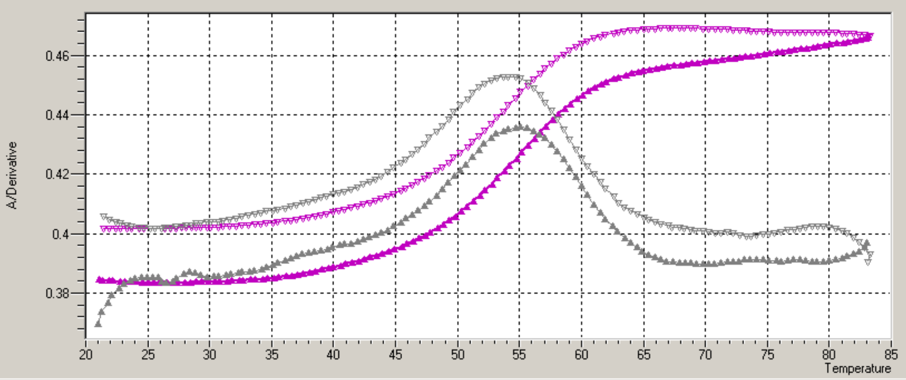

Cell	Show	Sample ID	Start Temp	End Temp	Tm [°C]	Tm [°C] Rev
2	☐	A - MJP6-590	20.0	85.0	62.4	61.9
3	☐	B - MJP6-591	20.0	85.0	56.1	54.7
4	☑	C - MJP6-592	20.0	85.0	55.1	54.1

Duplex of DNA **19** and ^{TL}DNA **22**

m_pal.MJP3-294
Slot No. 54 Sample ID MJP 3-294 C2 SupervisorID gpatt Lab Phone No. x UserID m_pal

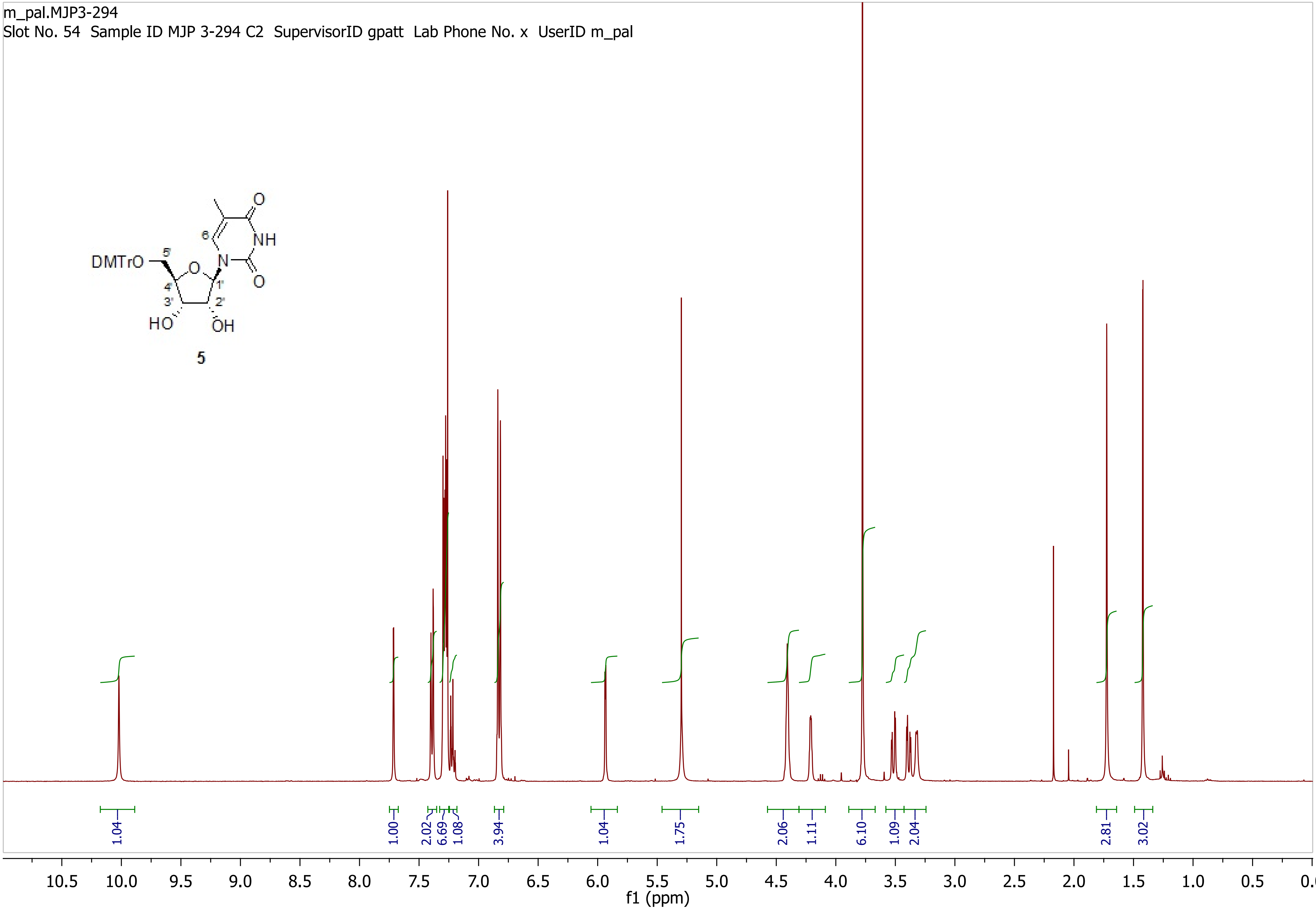

m_pal.MJP3-294
Slot No. 54 Sample ID MJP 3-294 C2 SupervisorID gpatt Lab Phone No. x UserID m_pal

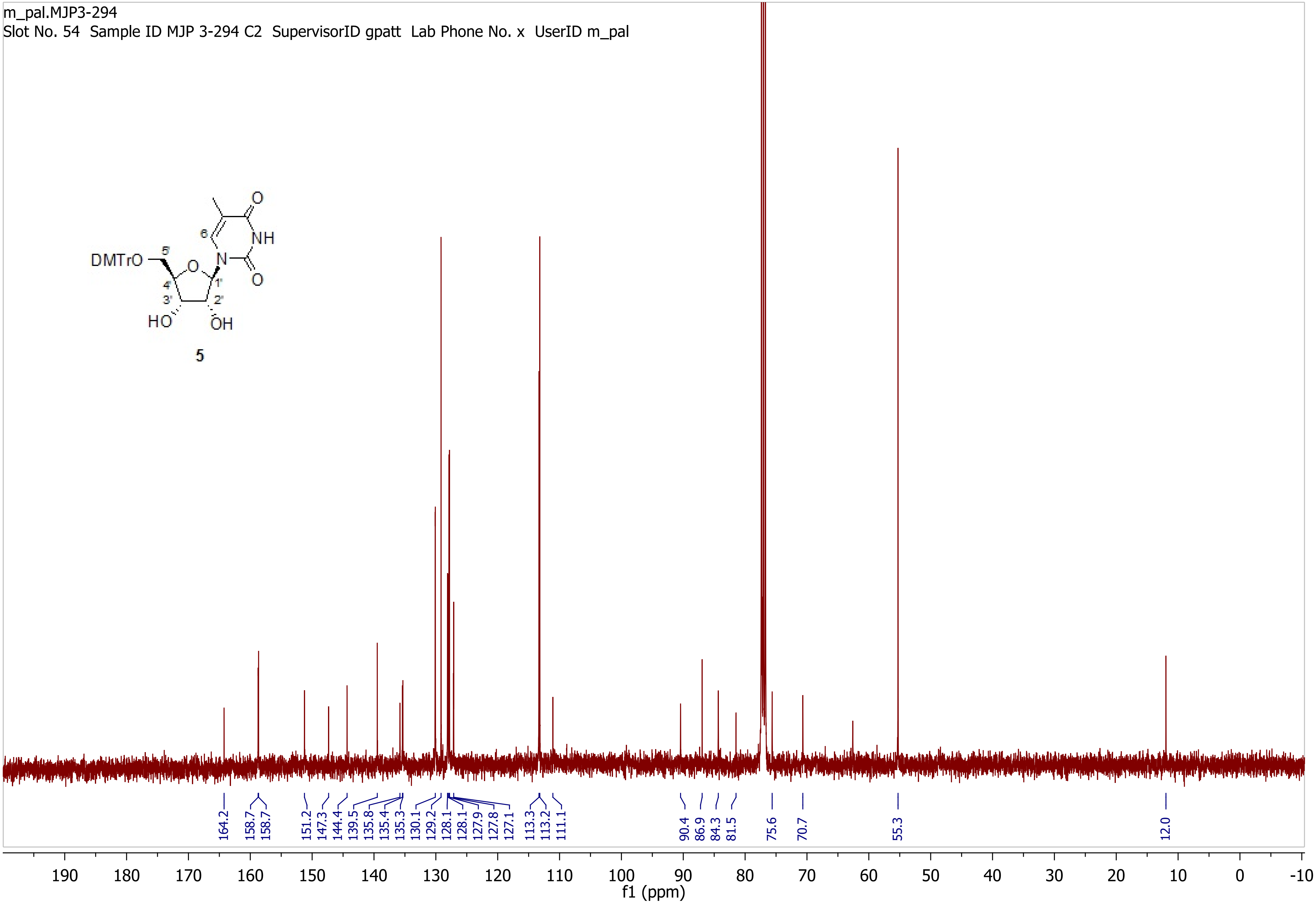

m_pal.MJP3-294
Slot No. 54 Sample ID MJP 3-294 C2 SupervisorID gpatt Lab Phone No. x UserID m_pal

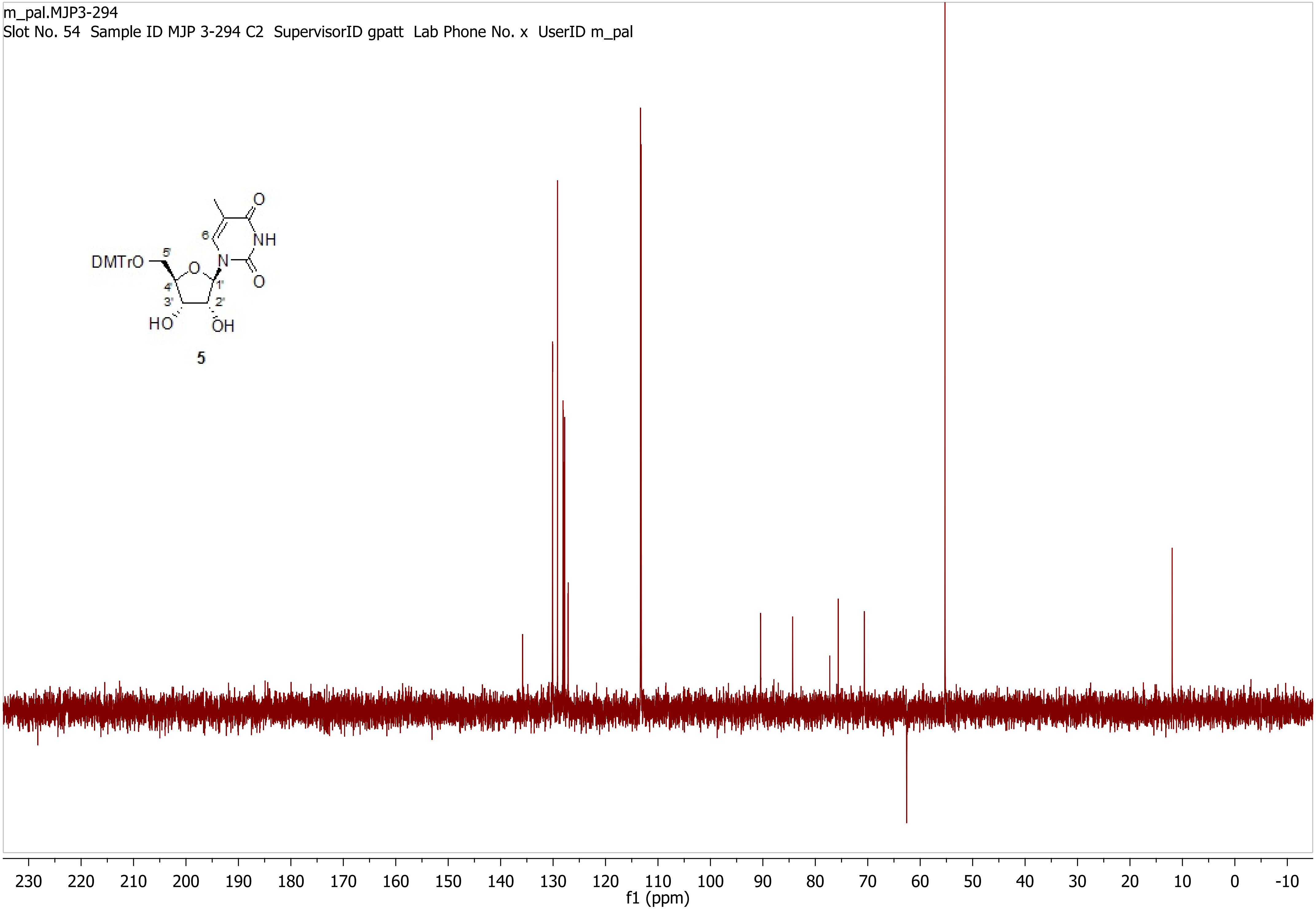

r_alh.RA587.1.fid
Slot No. 19 Sample ID RA587 SupervisorID hayes Lab Phone No. 13536 UserID r_alh

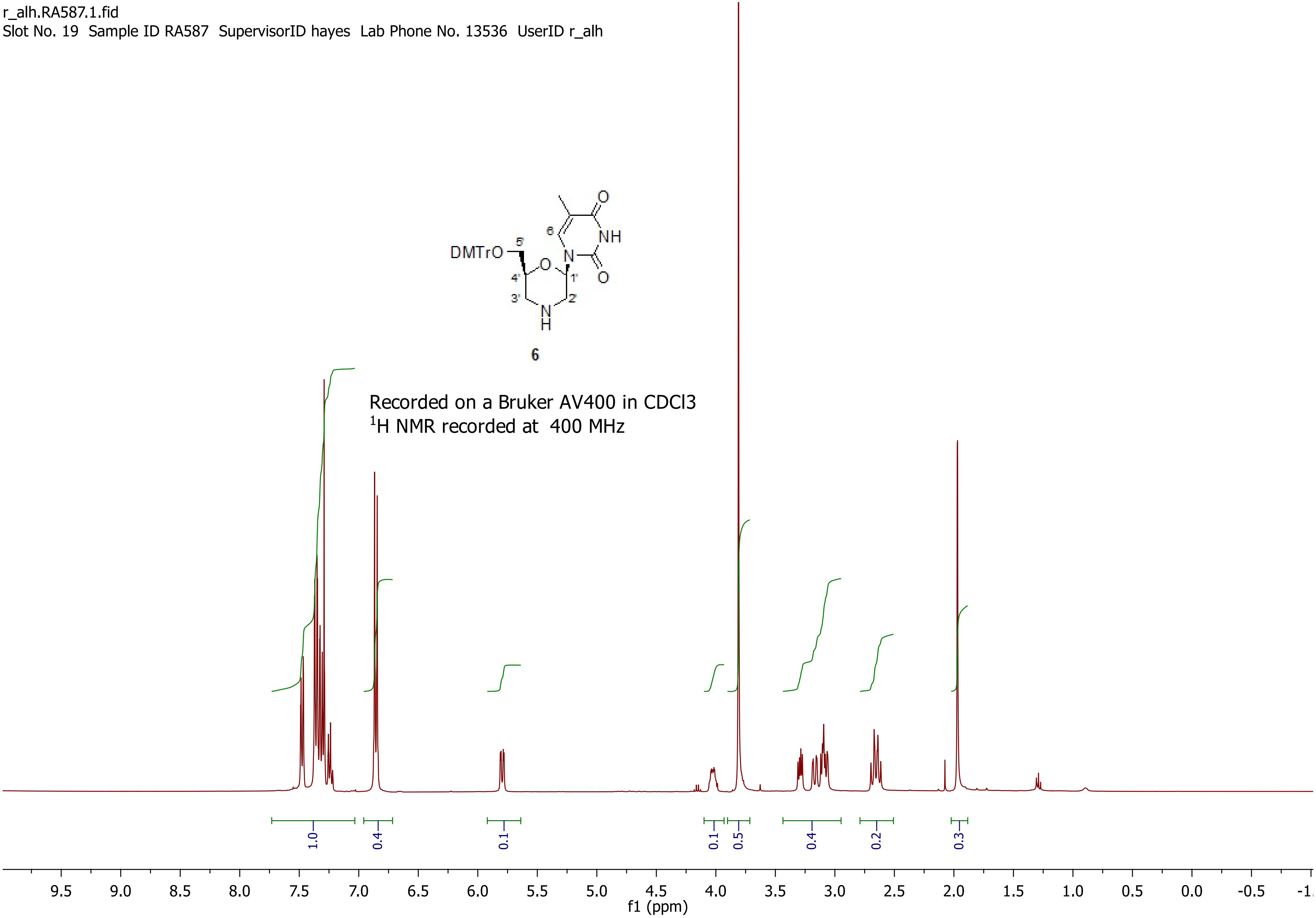

r_alh.RA587-DATA.2.fid
Slot No. 9 Sample ID RA587-DATA SupervisorID hayes Lab Phone No. 13536 UserID r_alh

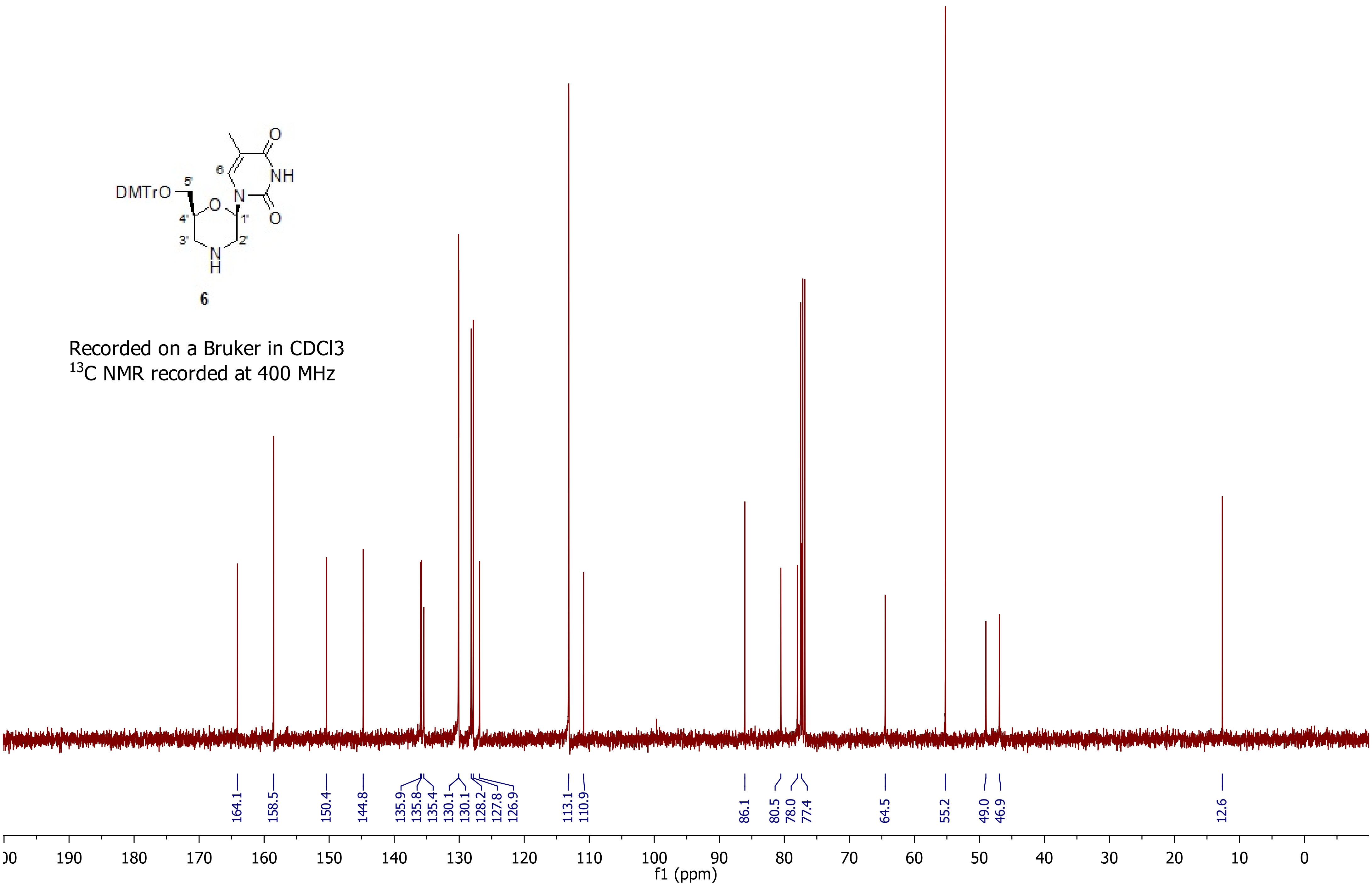

r_alh.RA587-DATA.4.fid
Slot No. 9 Sample ID RA587-DATA SupervisorID hayes Lab Phone No. 13536 UserID r_alh

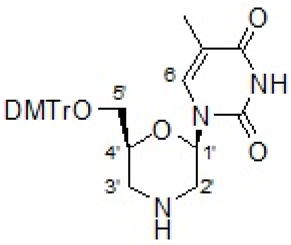

6

Recorded on a Bruker in CDCl3
DEPT NMR recorded at 400 MHz

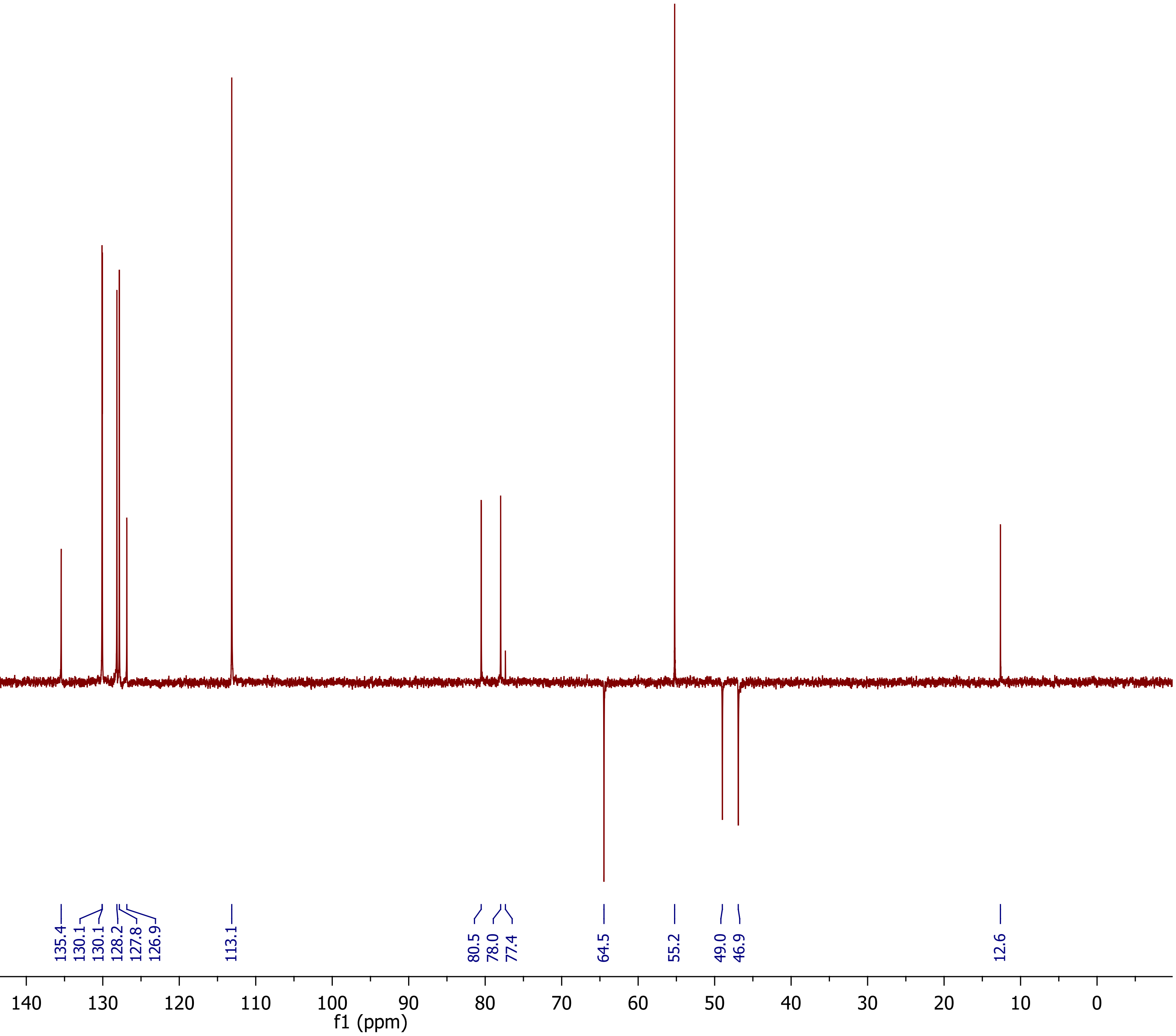

r_alh.RA586C.1.fid
Slot No. 13 Sample ID RA586C SupervisorID hayes Lab Phone No. 13536 UserID r_alh

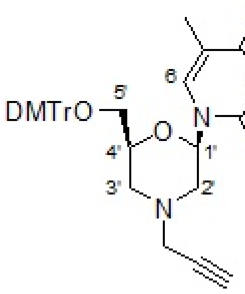

3

Recorded on a Bruker in CDCl3
^{1}H NMR recorded at 101 MHz

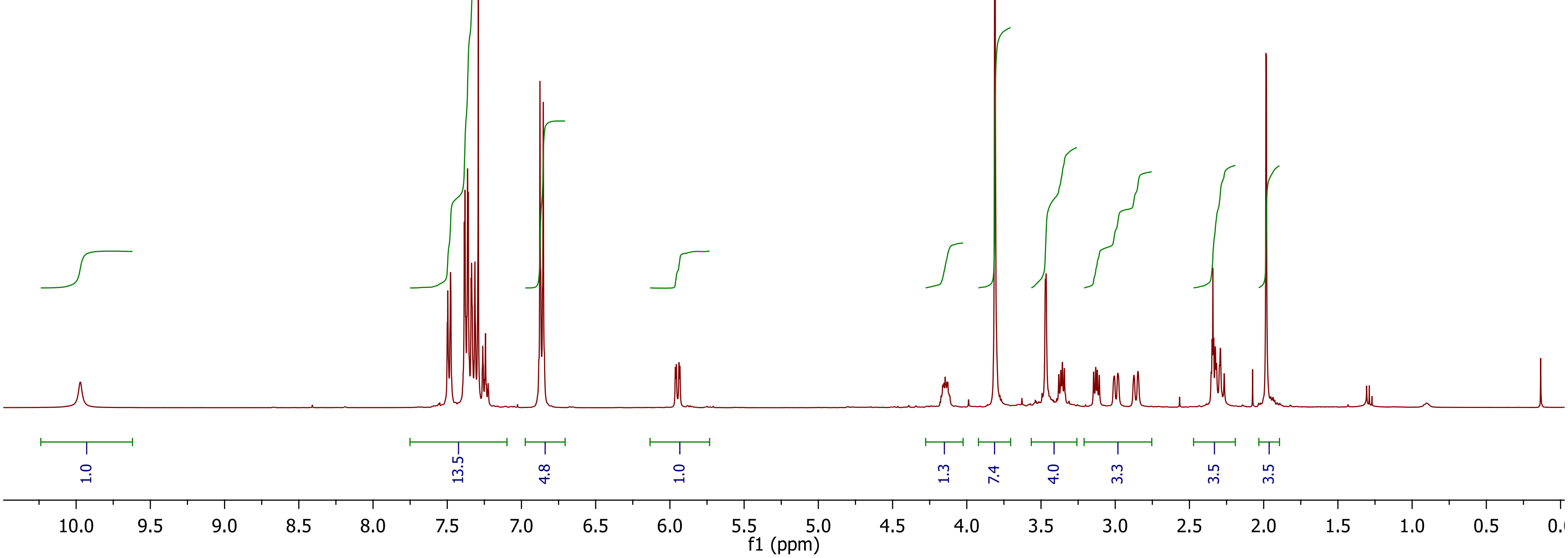

r_alh.RA586C-DATA.2.fid
Slot No. 8 Sample ID RA586C-DATA SupervisorID hayes Lab Phone No. 13536 UserID r_alh

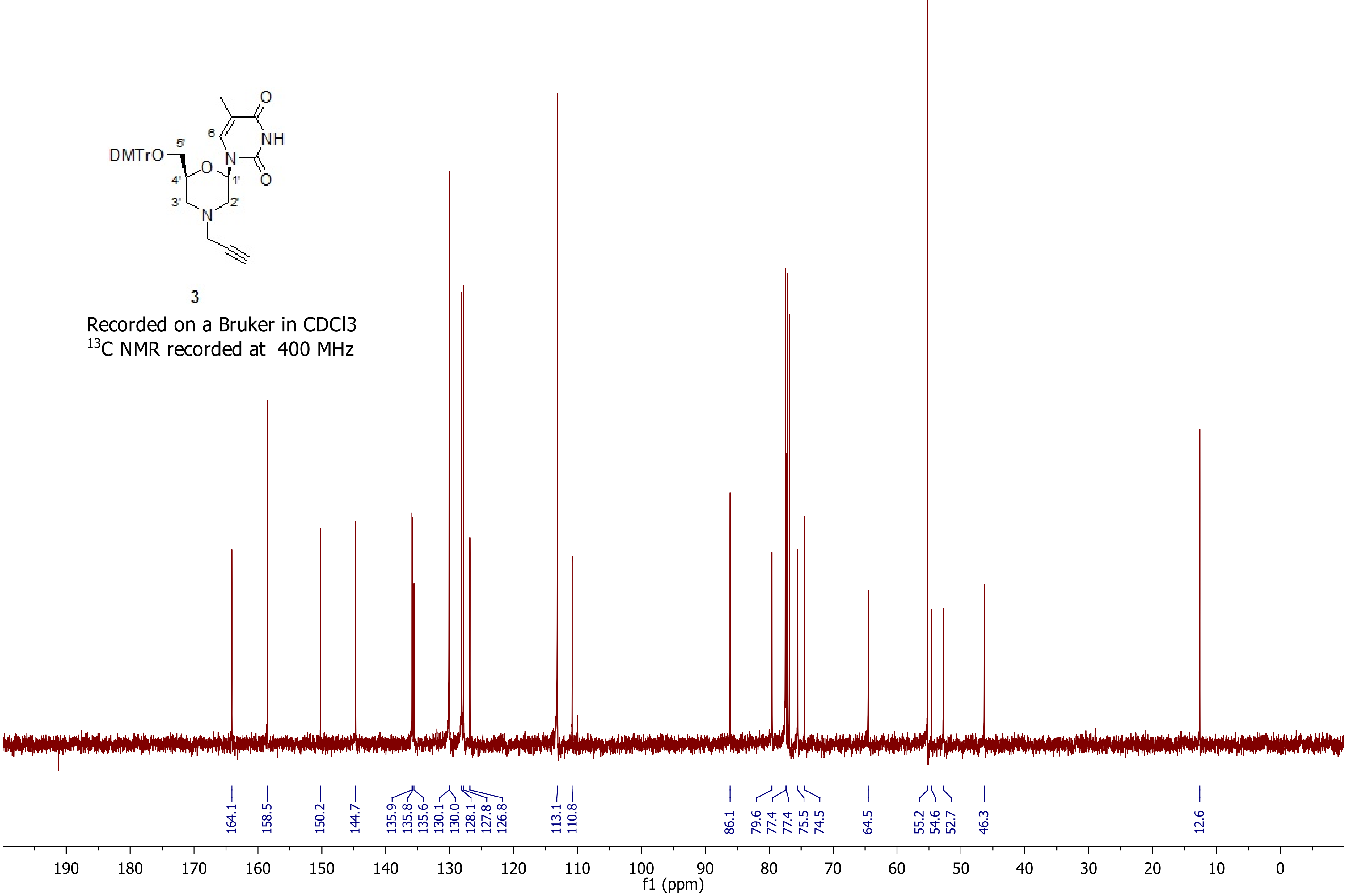

r_alh.RA586C-DATA.4.fid
Slot No. 8 Sample ID RA586C-DATA SupervisorID hayes Lab Phone No. 13536 UserID r_alh

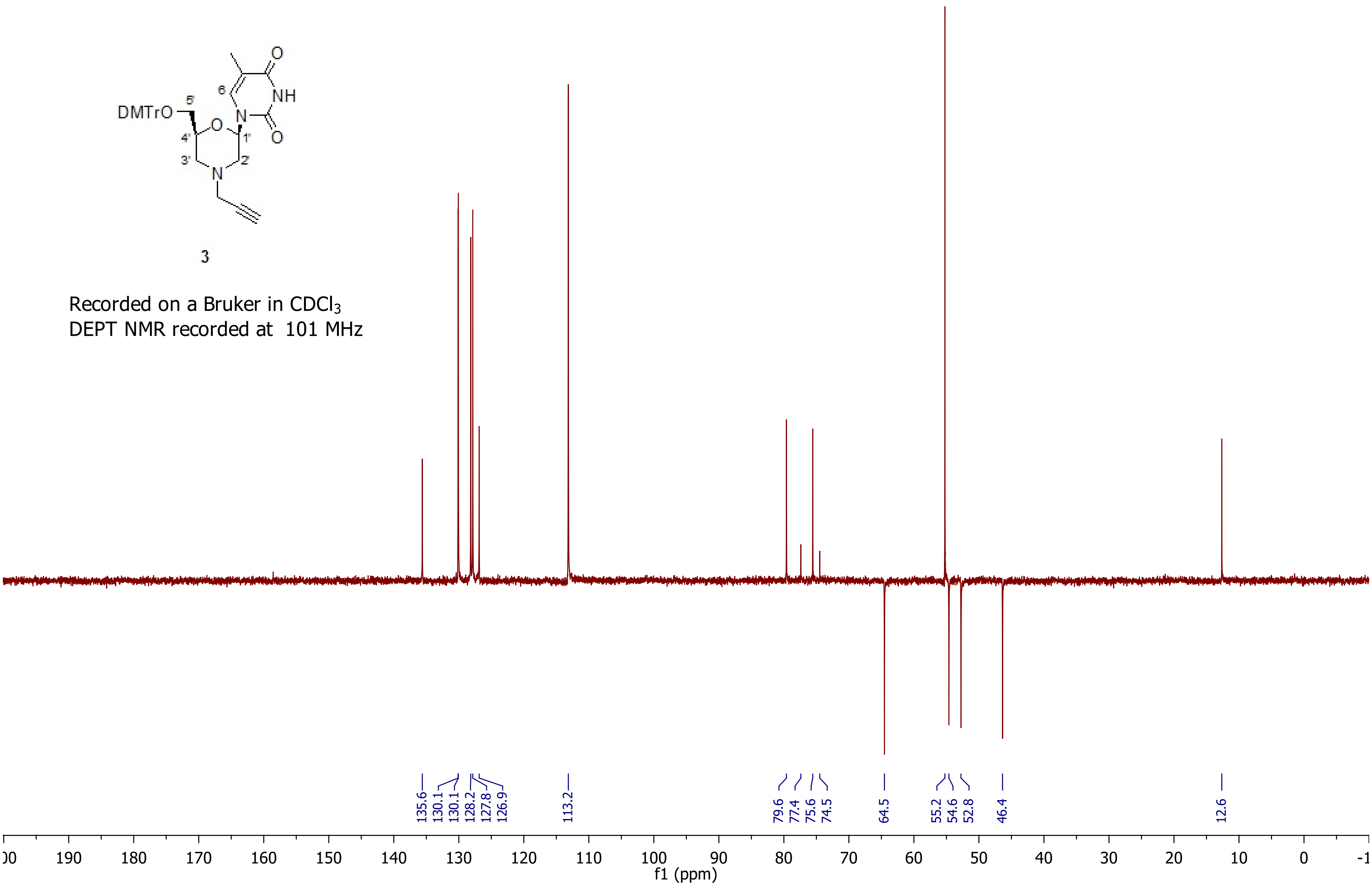

3

Recorded on a Bruker in $CDCl_3$
DEPT NMR recorded at 101 MHz

m_pal.MJP4-305
UserID m_pal SampleID MJP 4-305 col SupervisorID gpatt Lab Phone No. 14190 Slot Number 4

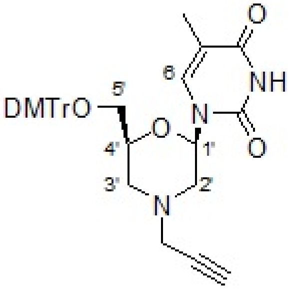

3

Recorded on a Bruker AV400 in C_6D_6
^{1}H NMR recorded at 400 MHz

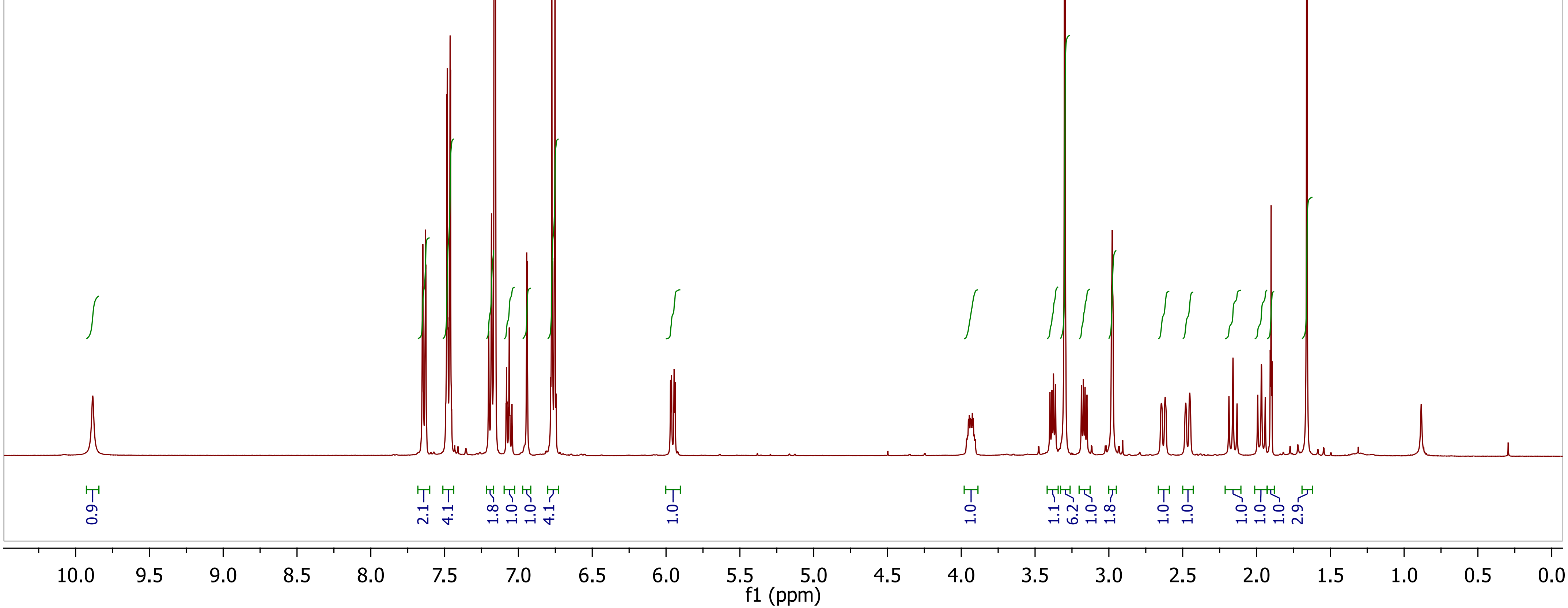

m_pal.MJP4-305
UserID m_pal SampleID MJP 4-305 col SupervisorID gpatt Lab Phone No. 14190 Slot Number 4

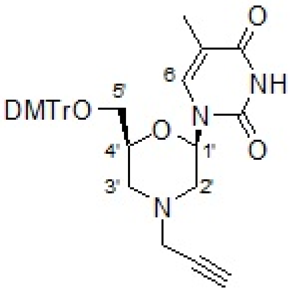

3

Recorded on a Bruker AV400 in C_6D_6
^{13}C NMR recorded at 101 MHz

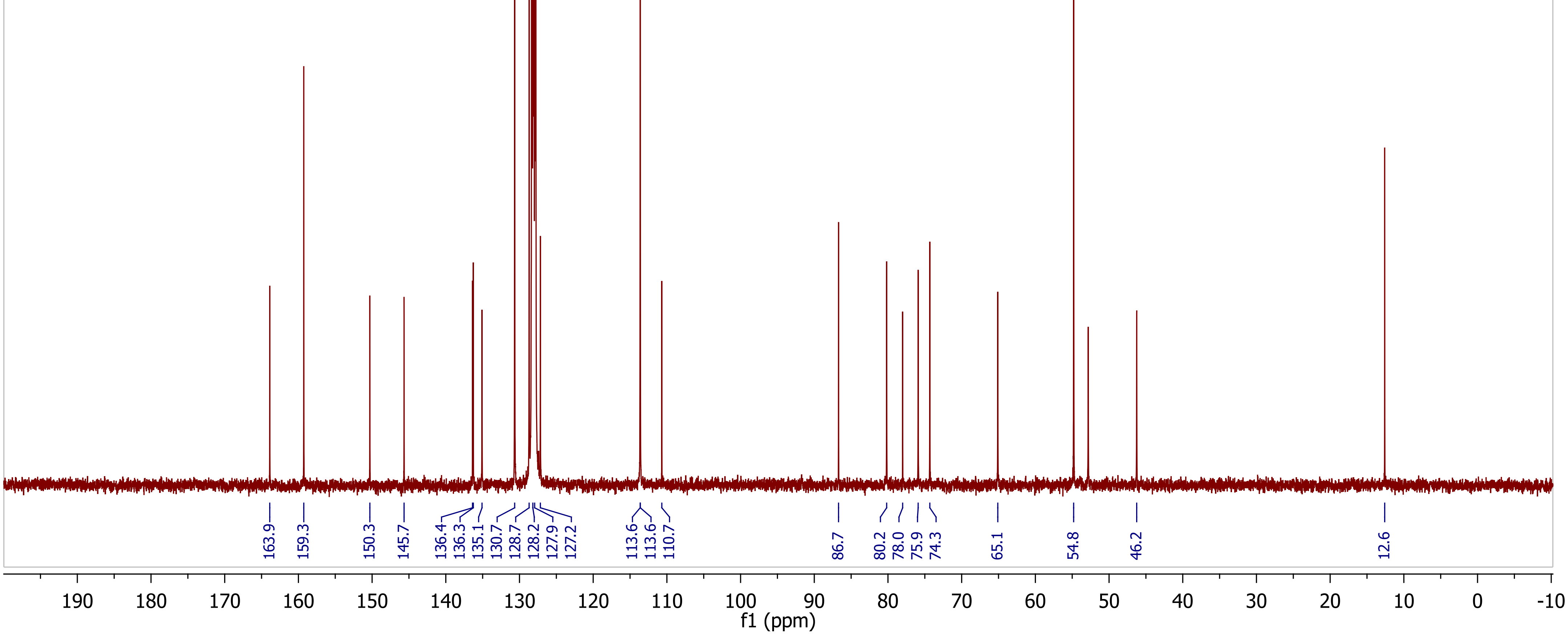

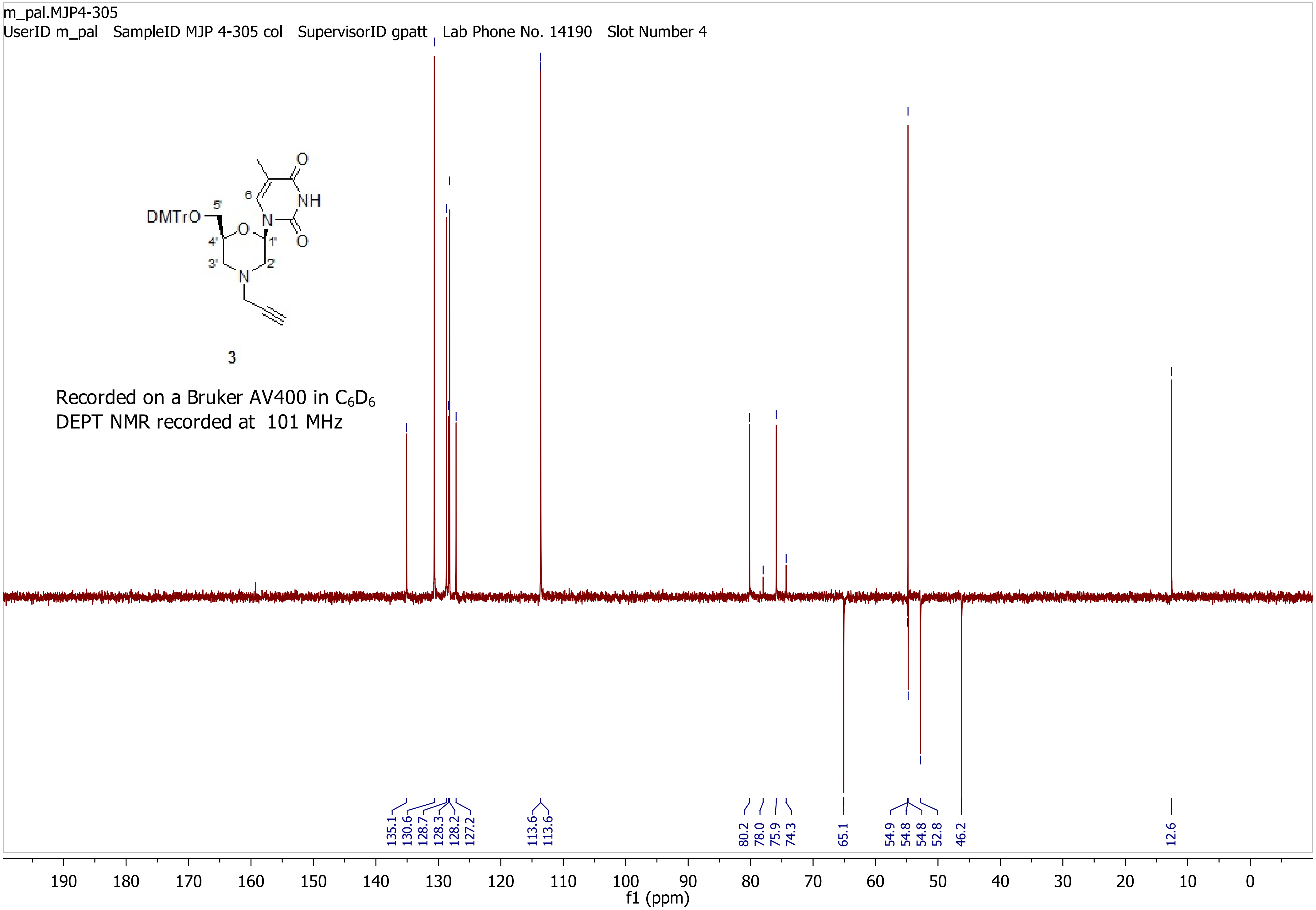

m_pal.MJP4-305
UserID m_pal SampleID MJP 4-305 col SupervisorID gpatt Lab Phone No. 14190 Slot Number 4
DMTrO
O
N
NH
O
O
N
3
Recorded on a Bruker AV400 in C_6D_6
DEPT NMR recorded at 101 MHz
135.1
130.6
128.7
128.3
128.2
127.2
113.6
113.6
80.2
78.0
75.9
74.3
65.1
54.9
54.8
54.8
52.8
46.2
12.6
190
180
170
160
150
140
130
120
110
100
90
80
70
60
50
40
30
20
10
0
f1 (ppm)

COSY recorded on a Bruker AV400 in C_6D_6

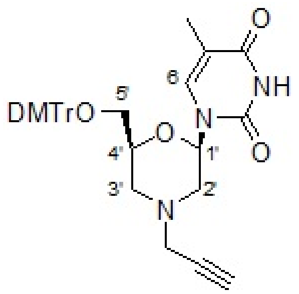

3

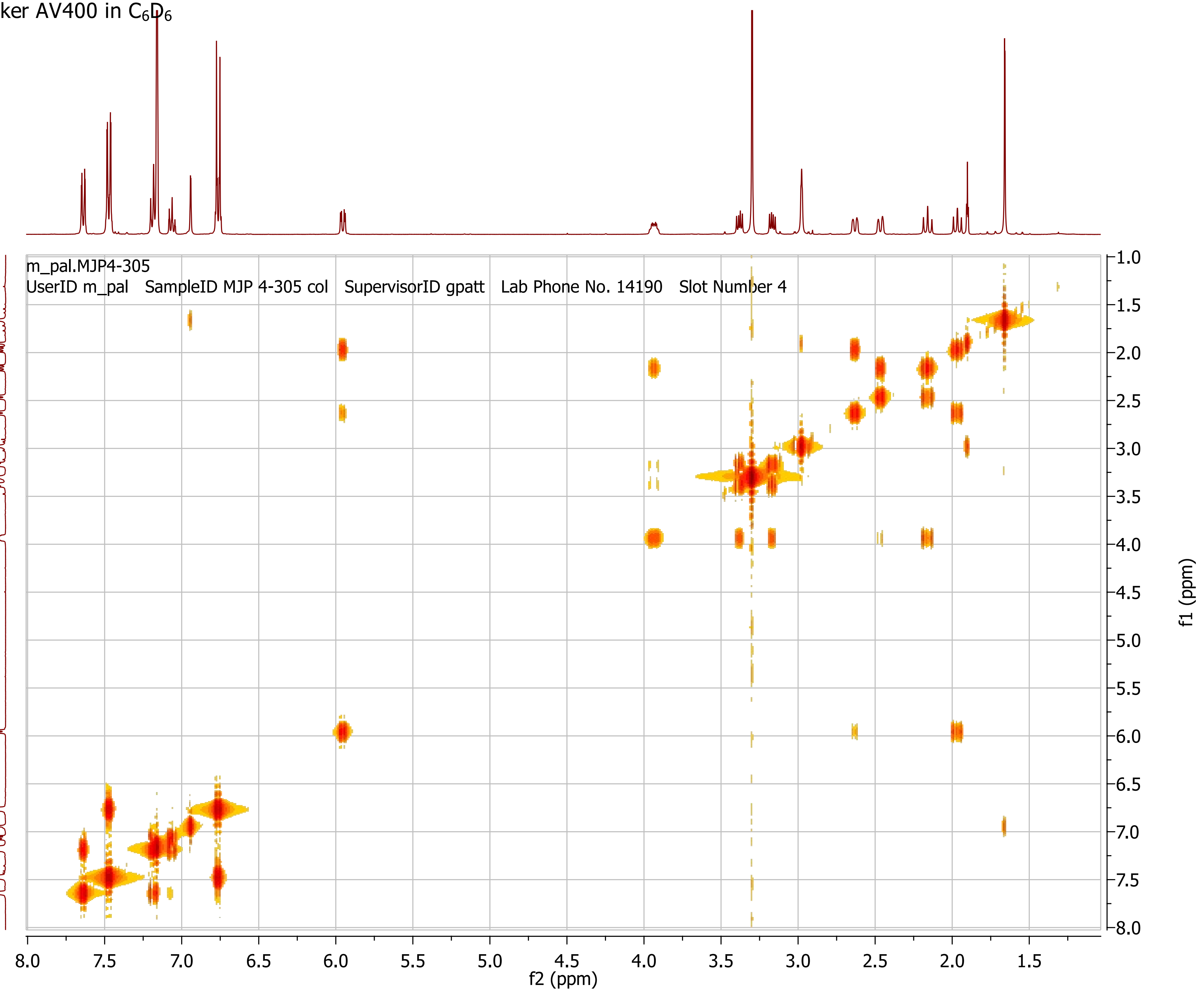

HSQC recorded on a Bruker AV400 in C_6D_6

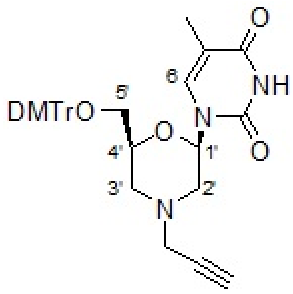

3

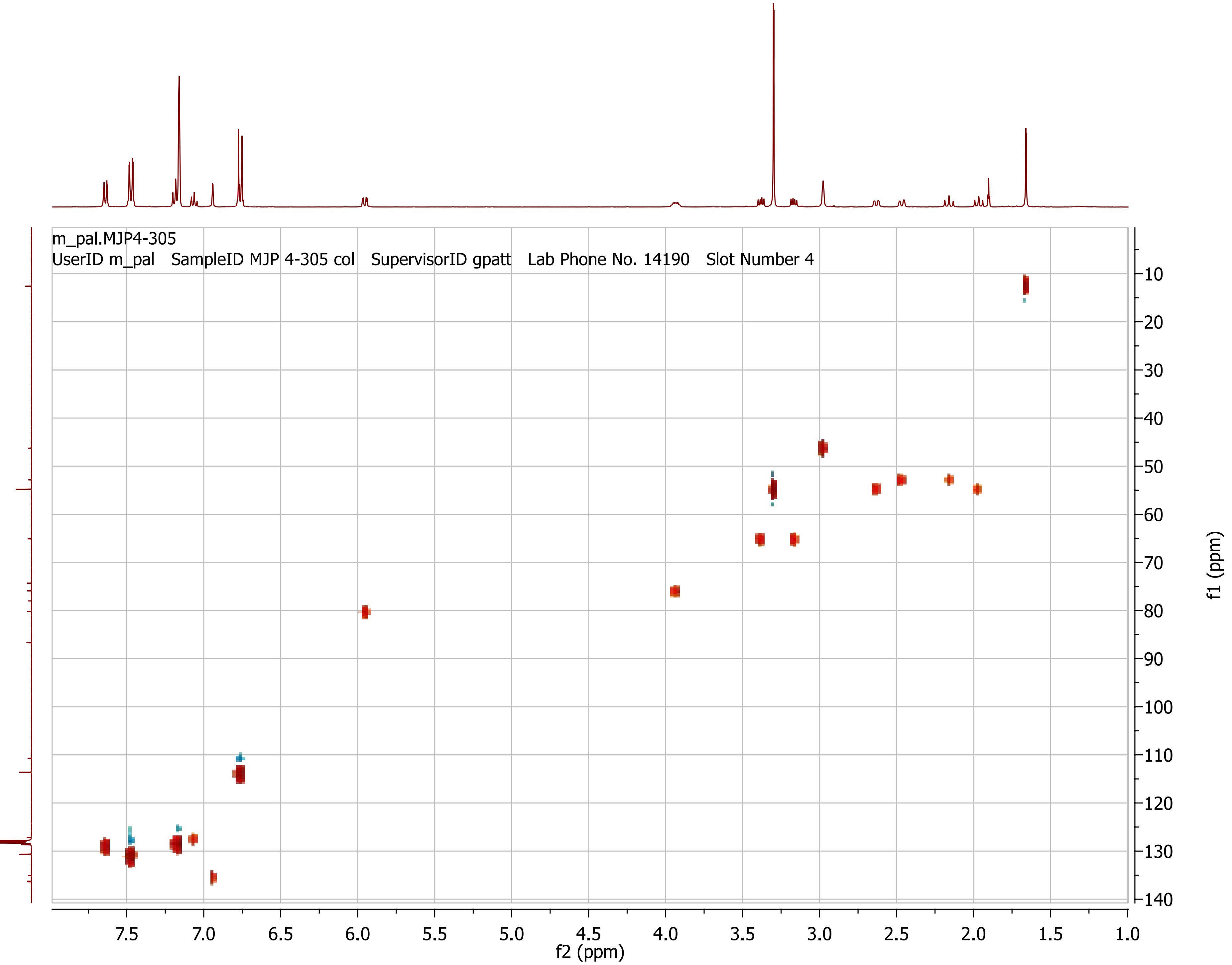

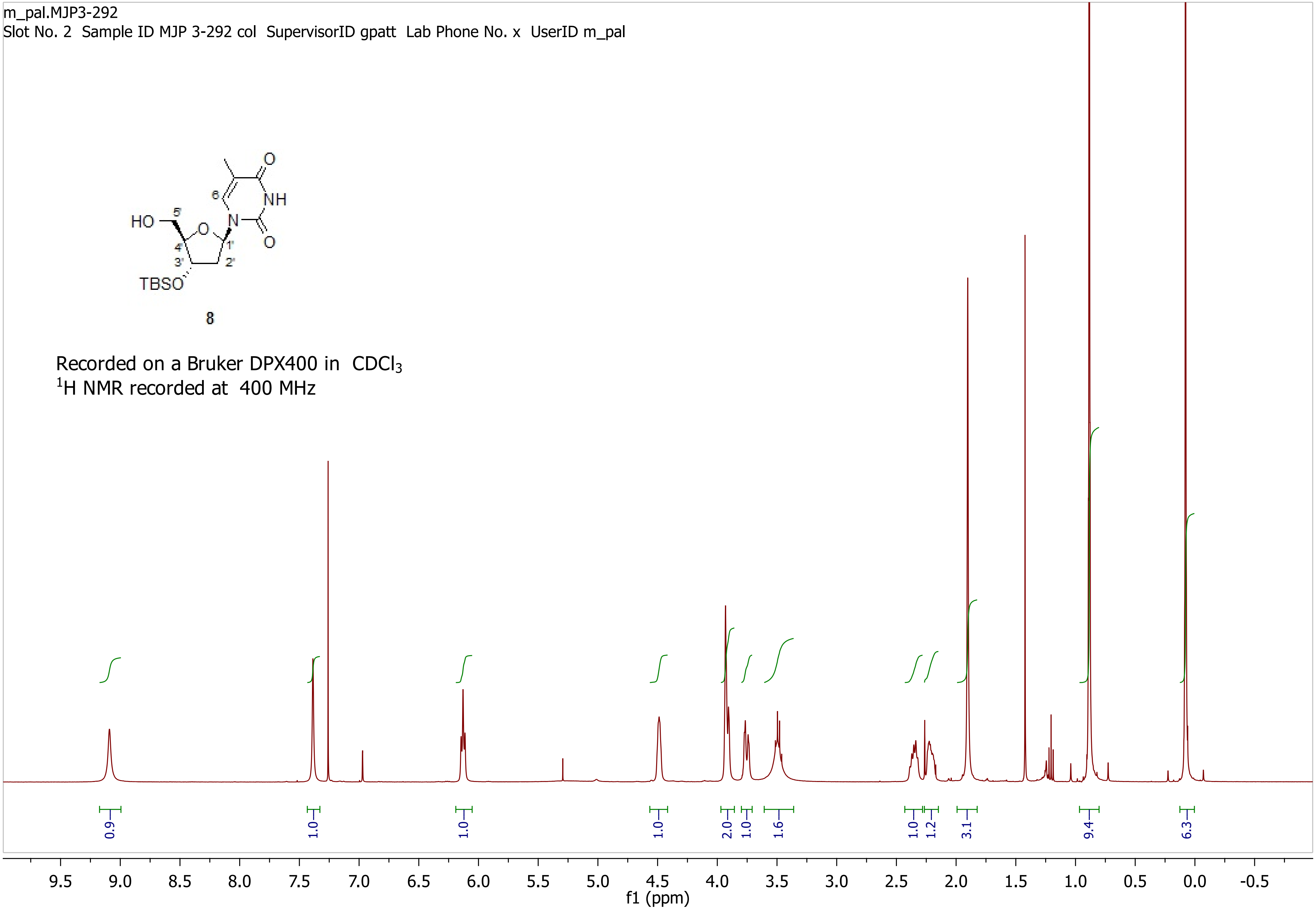
m_pal.MJP3-292
Slot No. 2 Sample ID MJP 3-292 col SupervisorID gpatt Lab Phone No. x UserID m_pal
HO
TBSO
NH
8
Recorded on a Bruker DPX400 in $CDCl_3$
^{1}H NMR recorded at 400 MHz
0.9
1.0
1.0
1.0
2.0
1.0
1.6
1.0
1.2
3.1
9.4
6.3
9.5 9.0 8.5 8.0 7.5 7.0 6.5 6.0 5.5 5.0 4.5 4.0 3.5 3.0 2.5 2.0 1.5 1.0 0.5 0.0 -0.5
f1 (ppm)

m_pal.MJP3-292
Slot No. 2 Sample ID MJP 3-292 col SupervisorID gpatt Lab Phone No. x UserID m_pal

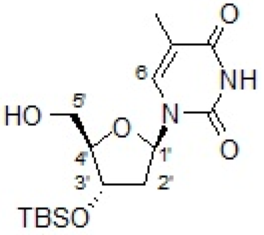

8

Recorded on a Bruker DPX400 in $CDCl_3$
^{13}C NMR recorded at 101 MHz

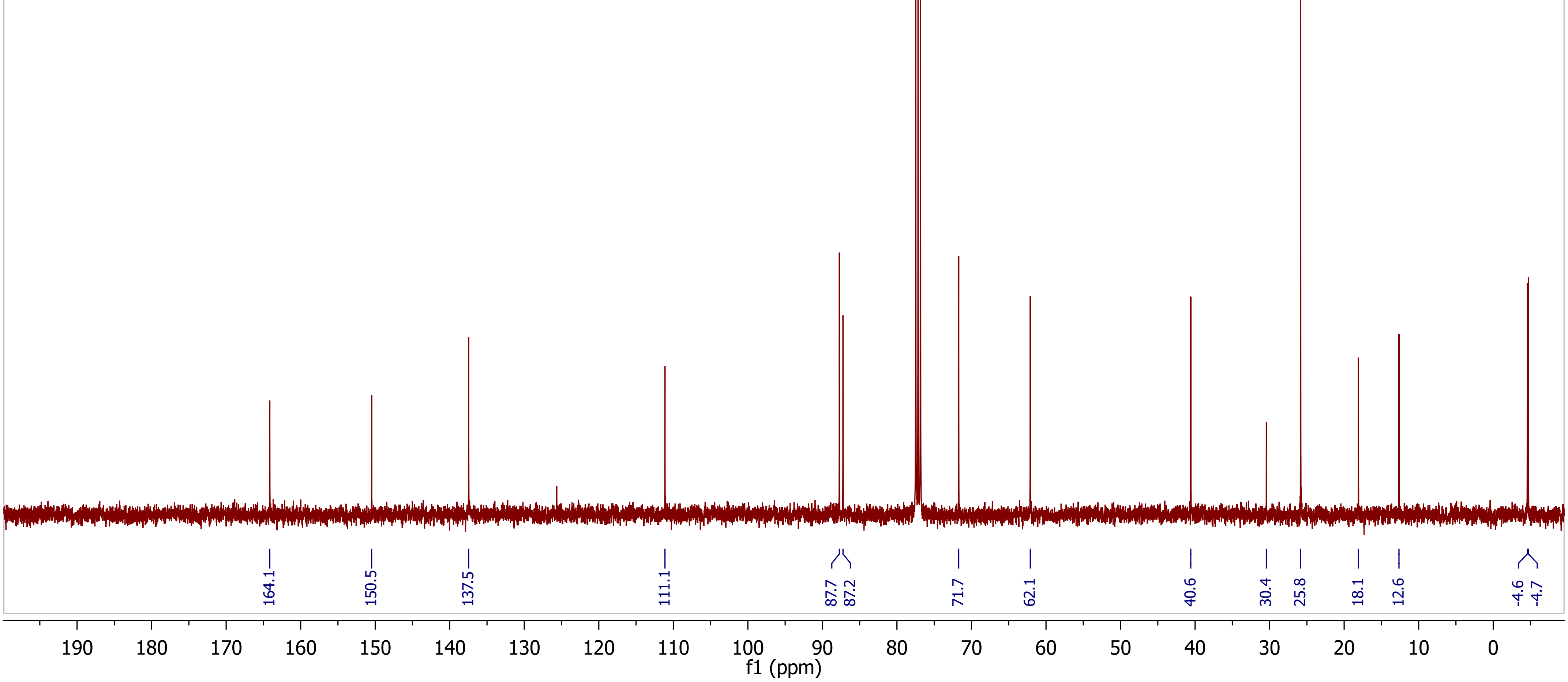

m_pal.MJP3-292
Slot No. 2 Sample ID MJP 3-292 col SupervisorID gpatt Lab Phone No. x UserID m_pal

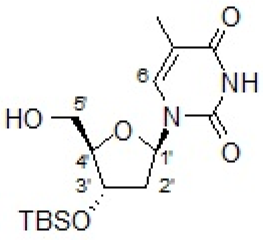

8

Recorded on a Bruker DPX400 in $CDCl_3$
DEPT NMR recorded at 101 MHz

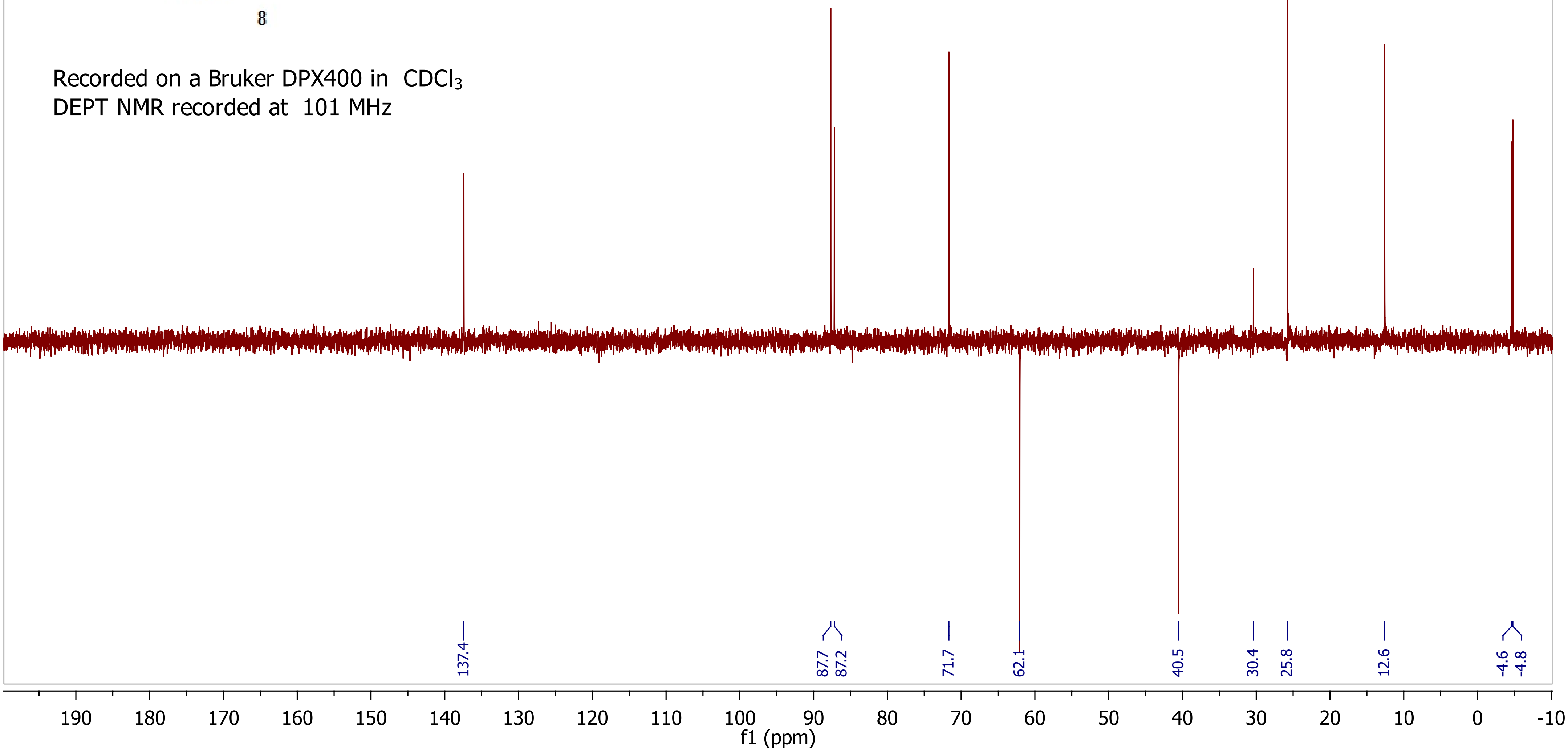

m_pal.MJP3-291
Slot No. 1 Sample ID MJP 3-291 SupervisorID gpatt Lab Phone No. x UserID m_pal

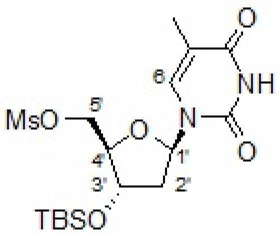

9

Recorded on a Brucker DPX-400 in $CDCl_3$
^{1}H NMR recorded at 400 MHz

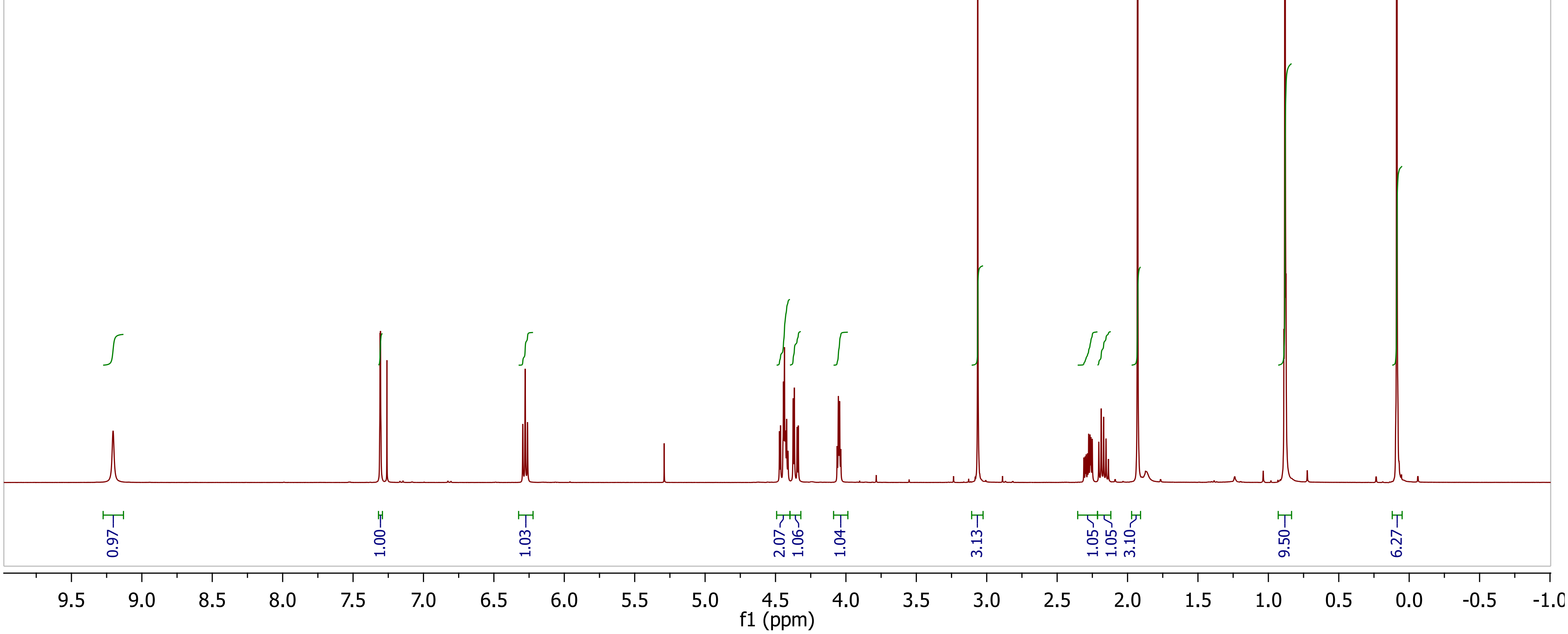

m_pal.MJP3-291
Slot No. 1 Sample ID MJP 3-291 SupervisorID gpatt Lab Phone No. x UserID m_pal

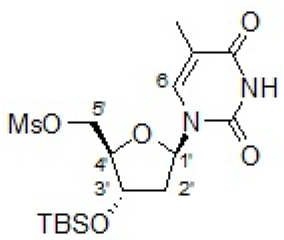

9

Recorded on a Brucker DPX-400 in $CDCl_3$
^{13}C NMR recorded at 101 MHz

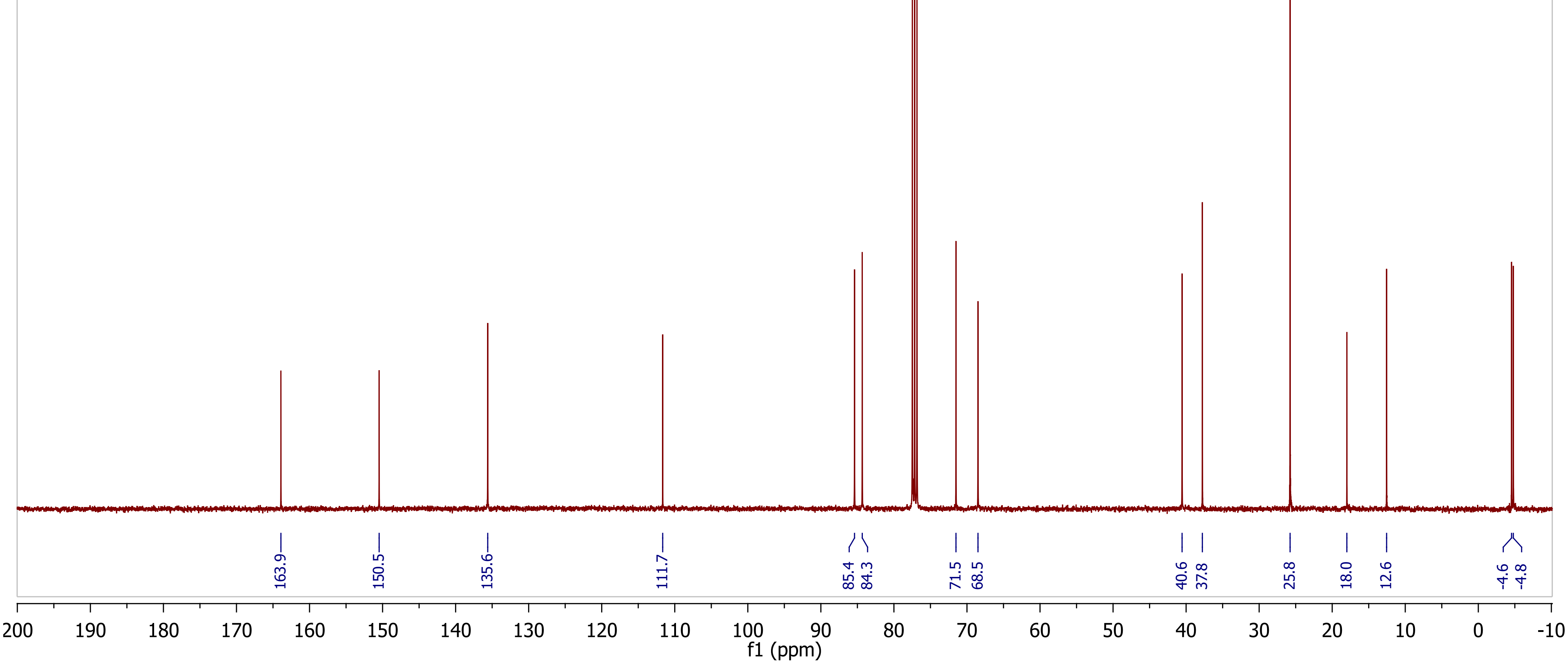

m_pal.mjp3-293
UserID m_pal SampleID mjp3-293 SupervisorID gpatt Lab Phone No. 14190 Slot 2

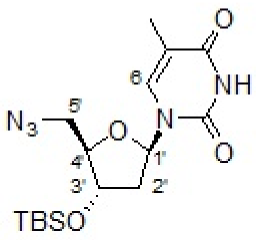

10

Recorded on a Brucker AV3-500 in $CDCl_3$
^{1}H NMR recorded at 500 MHz

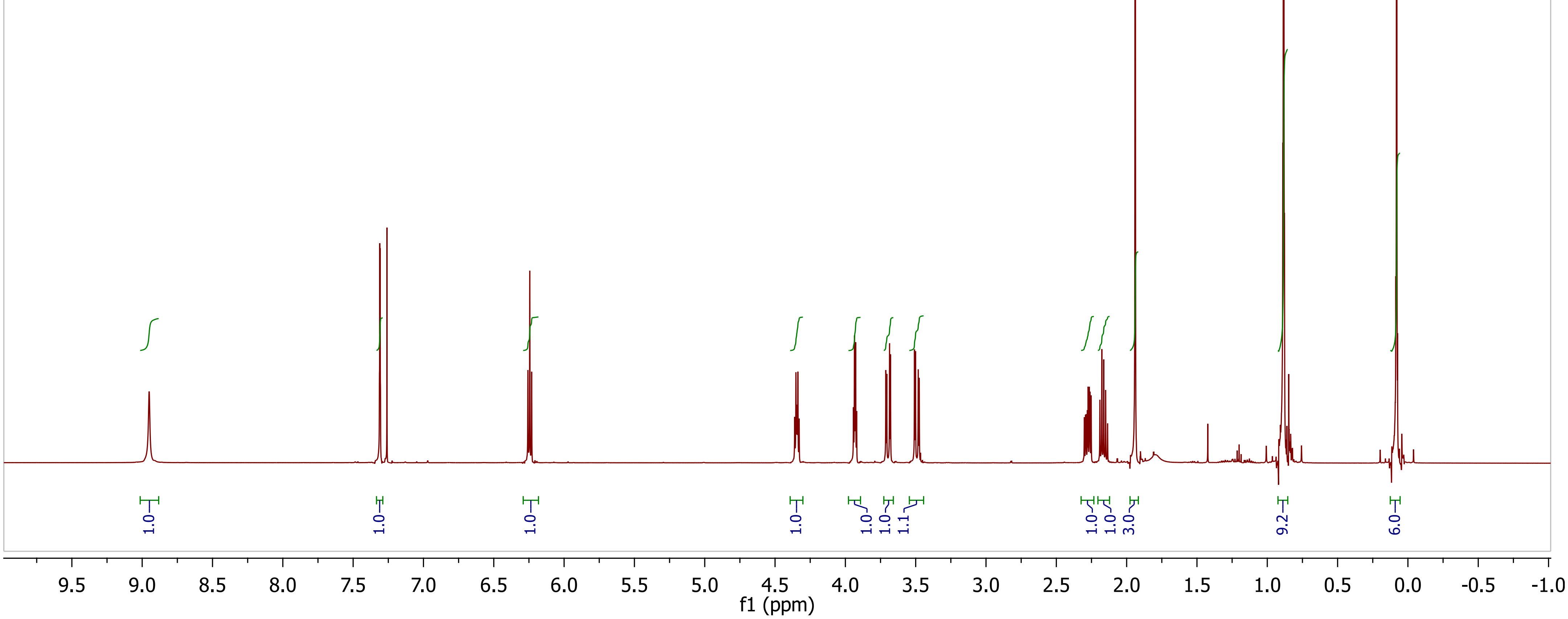

m_pal.mjp3-293
UserID m_pal SampleID mjp3-293 SupervisorID gpatt Lab Phone No. 14190 Slot 2

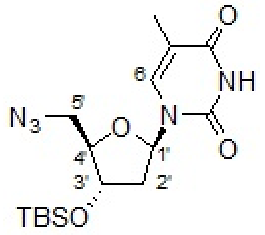

10

Recorded on a Bruker AV3-500 in $CDCl_3$
^{1}H NMR recorded at 500 MHz

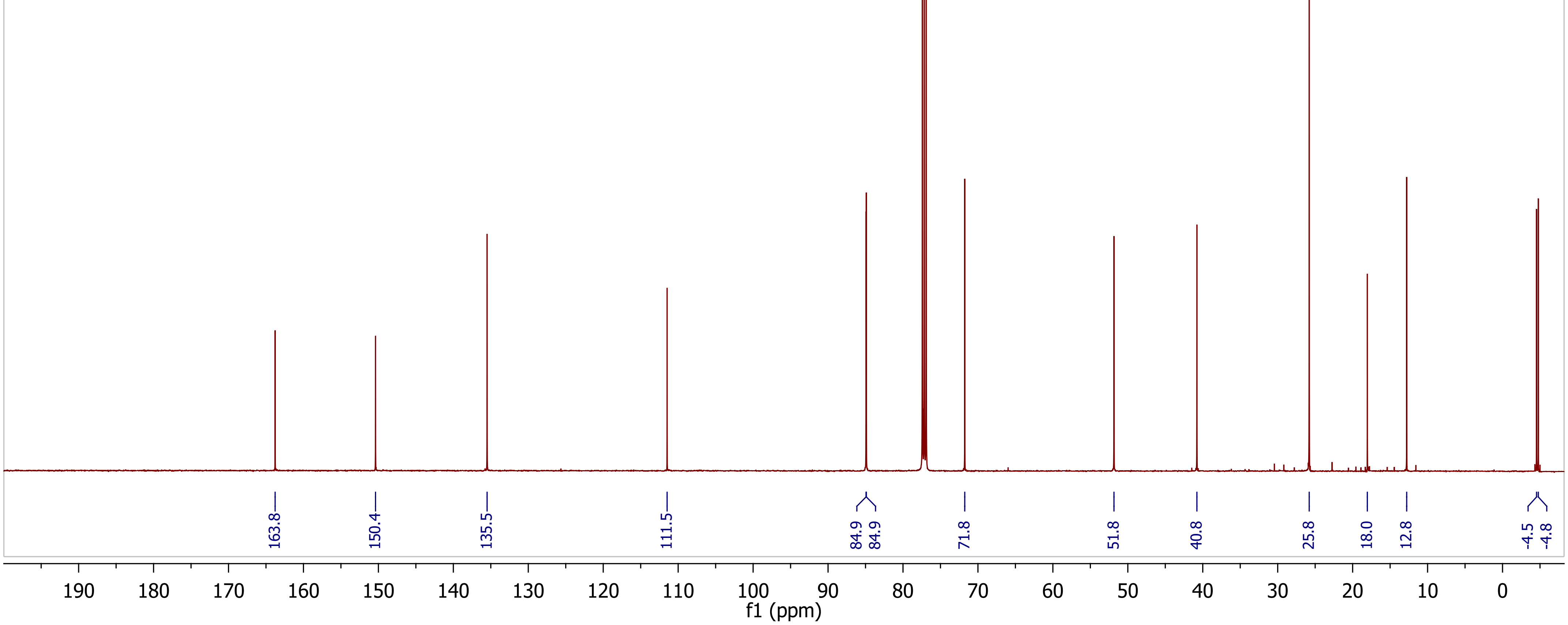

m_pal.mjp3-293
UserID m_pal SampleID mjp3-293 SupervisorID gpatt Lab Phone No. 14190 Slot 2

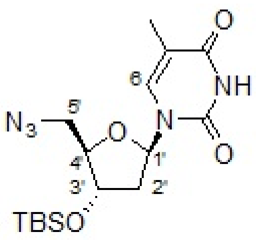

10

Recorded on a Bruker AV3-500 in $CDCl_3$
^{1}H NMR recorded at 500 MHz

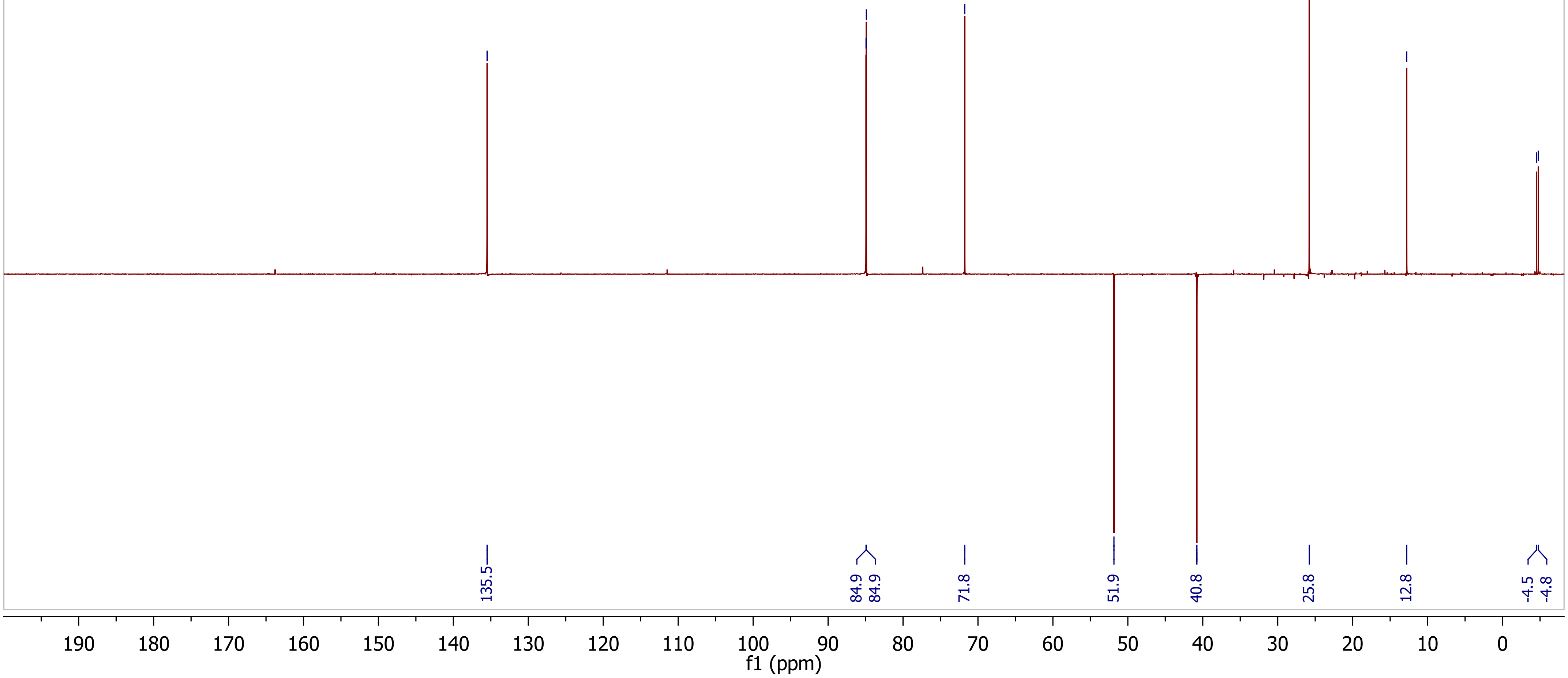

HSQC Recorded on a Bruker AV3-500 in $CDCl_3$

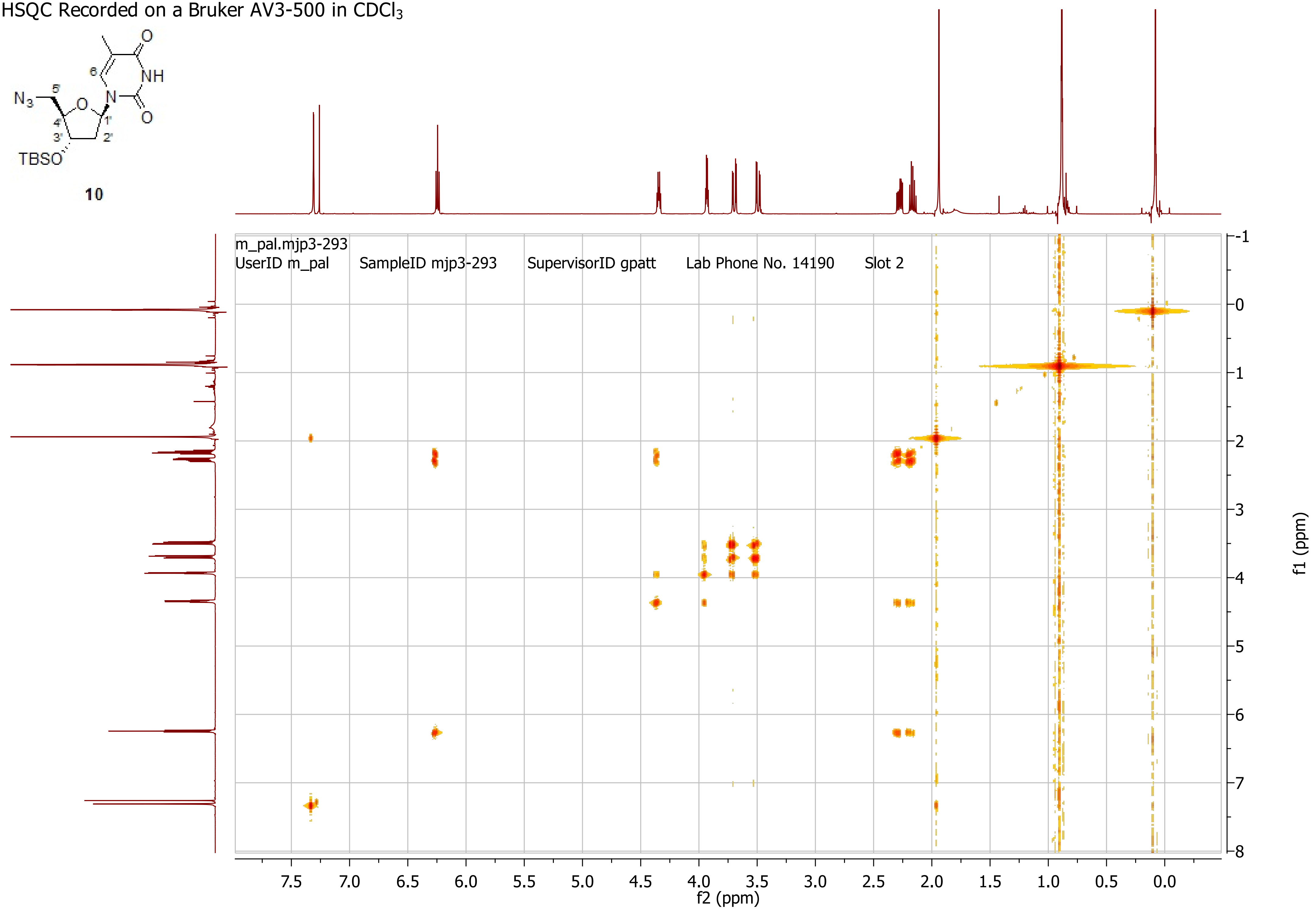

HSQC Recorded on a Bruker AV3-500 in $CDCl_3$

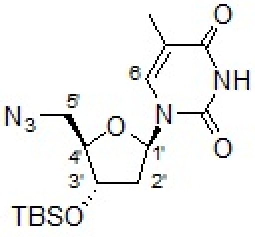

10

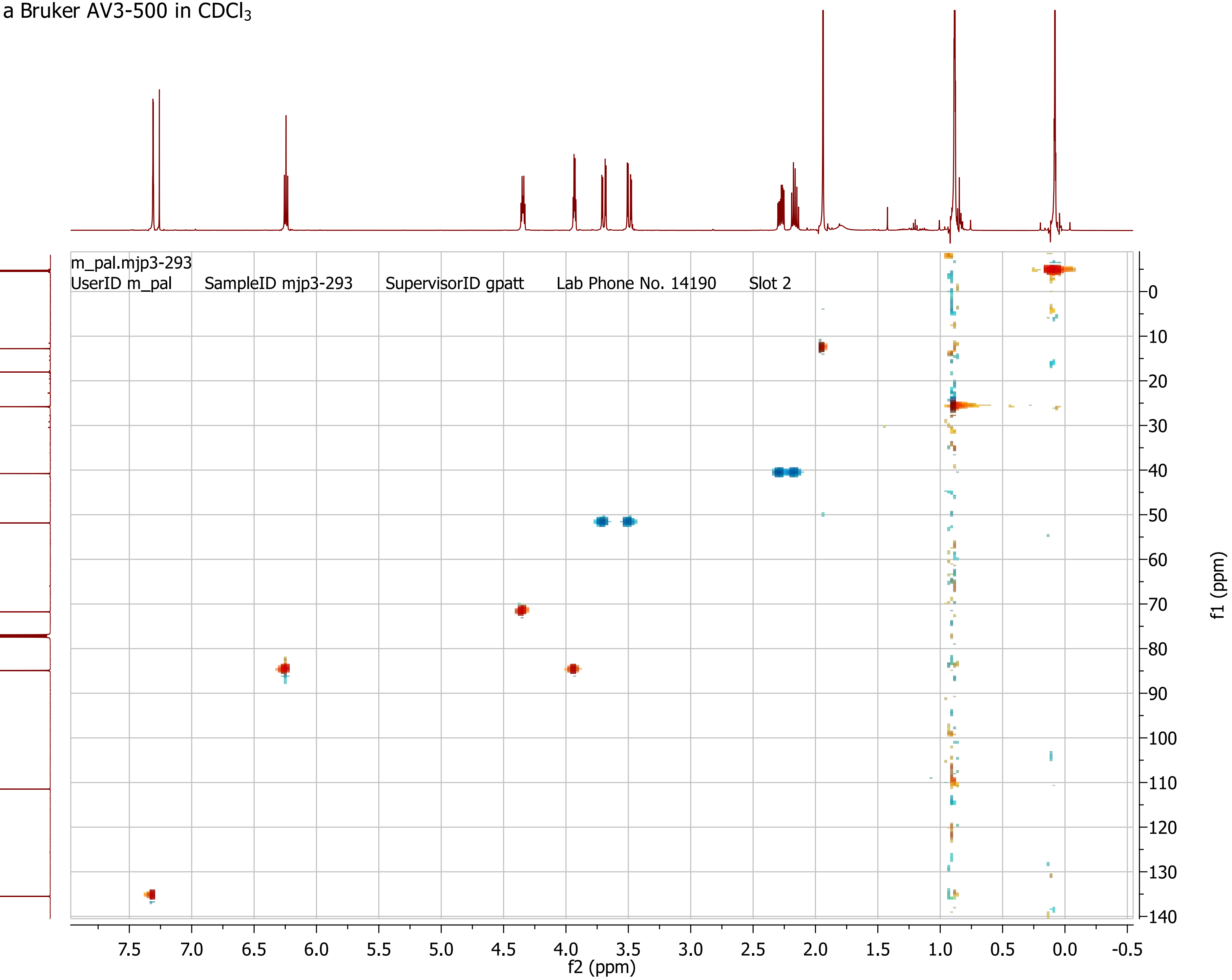

m_pal.mjp4-308
UserID m_pal SampleID mjp4-308 SupervisorID gpatt Lab Phone No. 14190 Slot 2

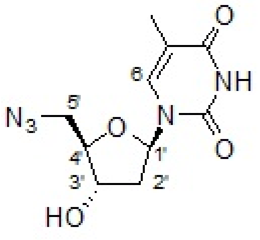

4

Recorded on a Bruker AV3-500 in D4-MeOD
^{1}H NMR recorded at 500 MHz

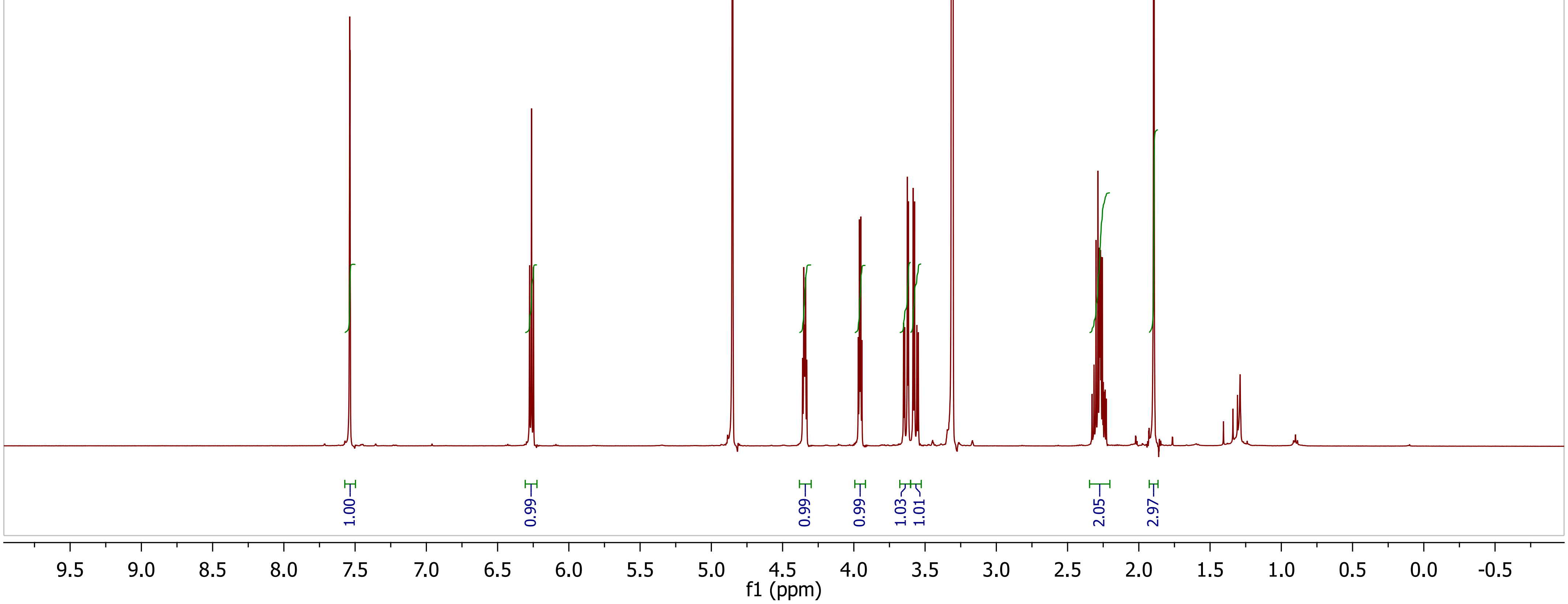

m_pal.mjp4-308
UserID m_pal SampleID mjp4-308 SupervisorID gpatt Lab Phone No. 14190 Slot 2

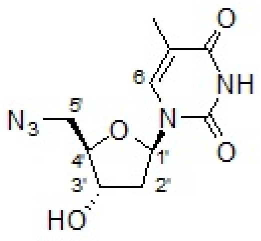

4

Recorded on a Bruker AV3-500 in D4-MeOD
^{1}H NMR recorded at 126 MHz

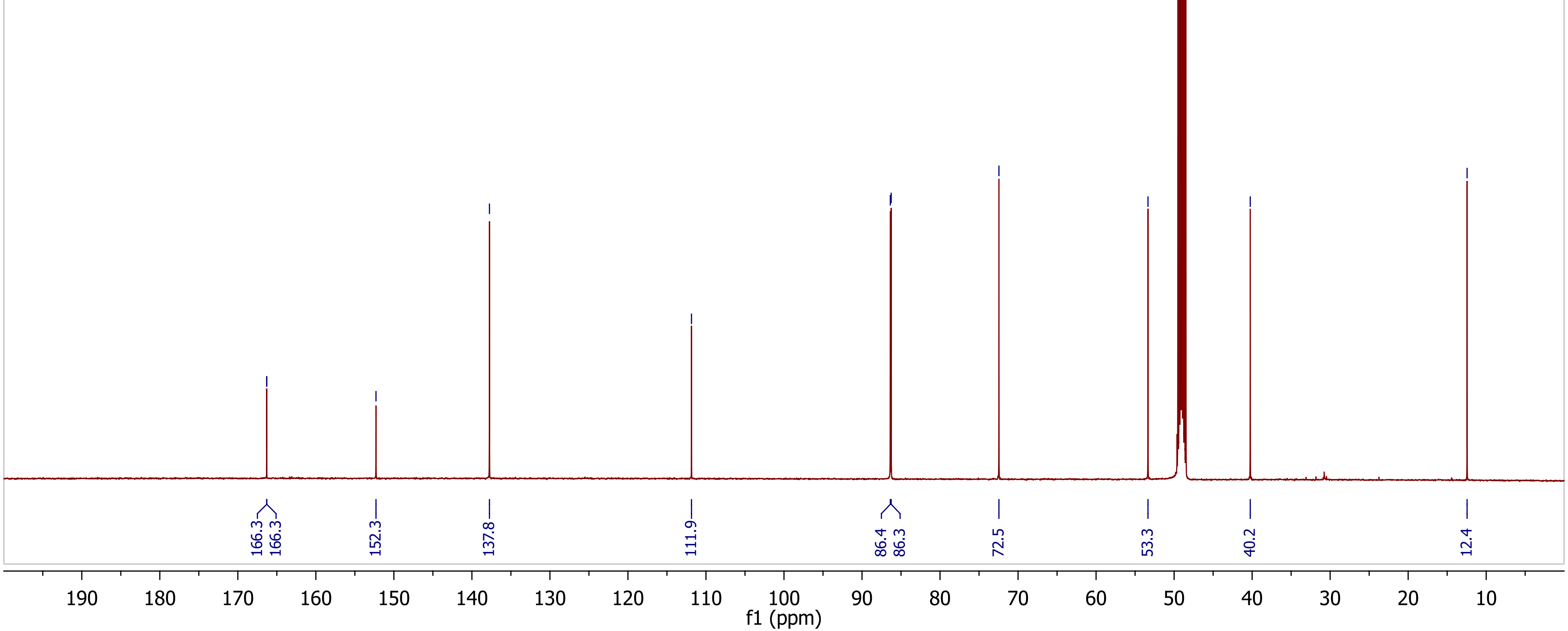

m_pal.mjp4-308
UserID m_pal SampleID mjp4-308 SupervisorID gpatt Lab Phone No. 14190 Slot 2

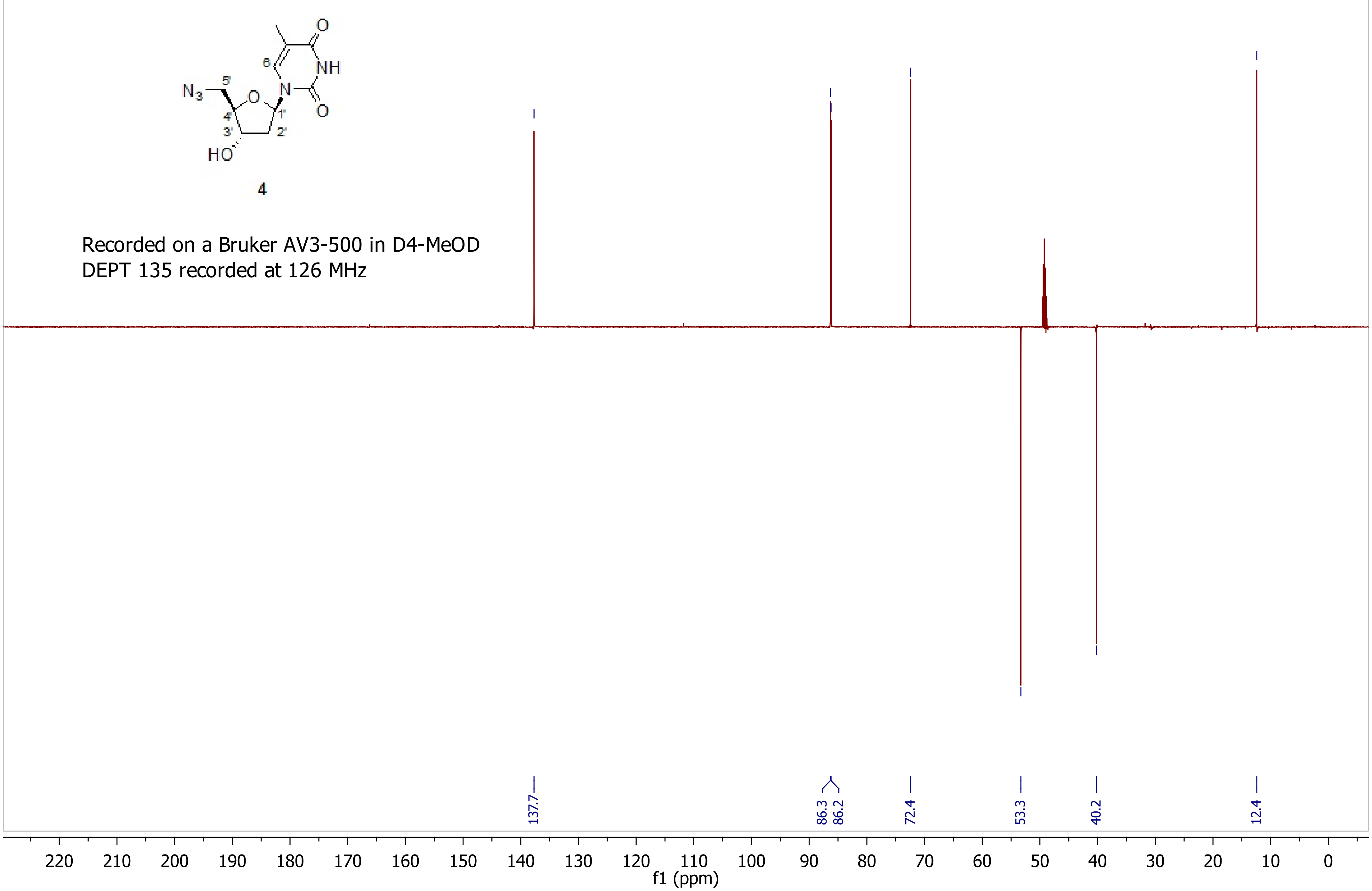

COSY Recorded on a Bruker AV3-500 in D4-MeOD

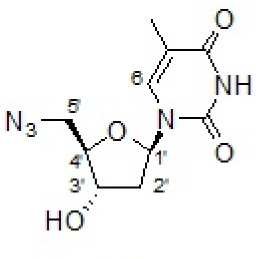

4

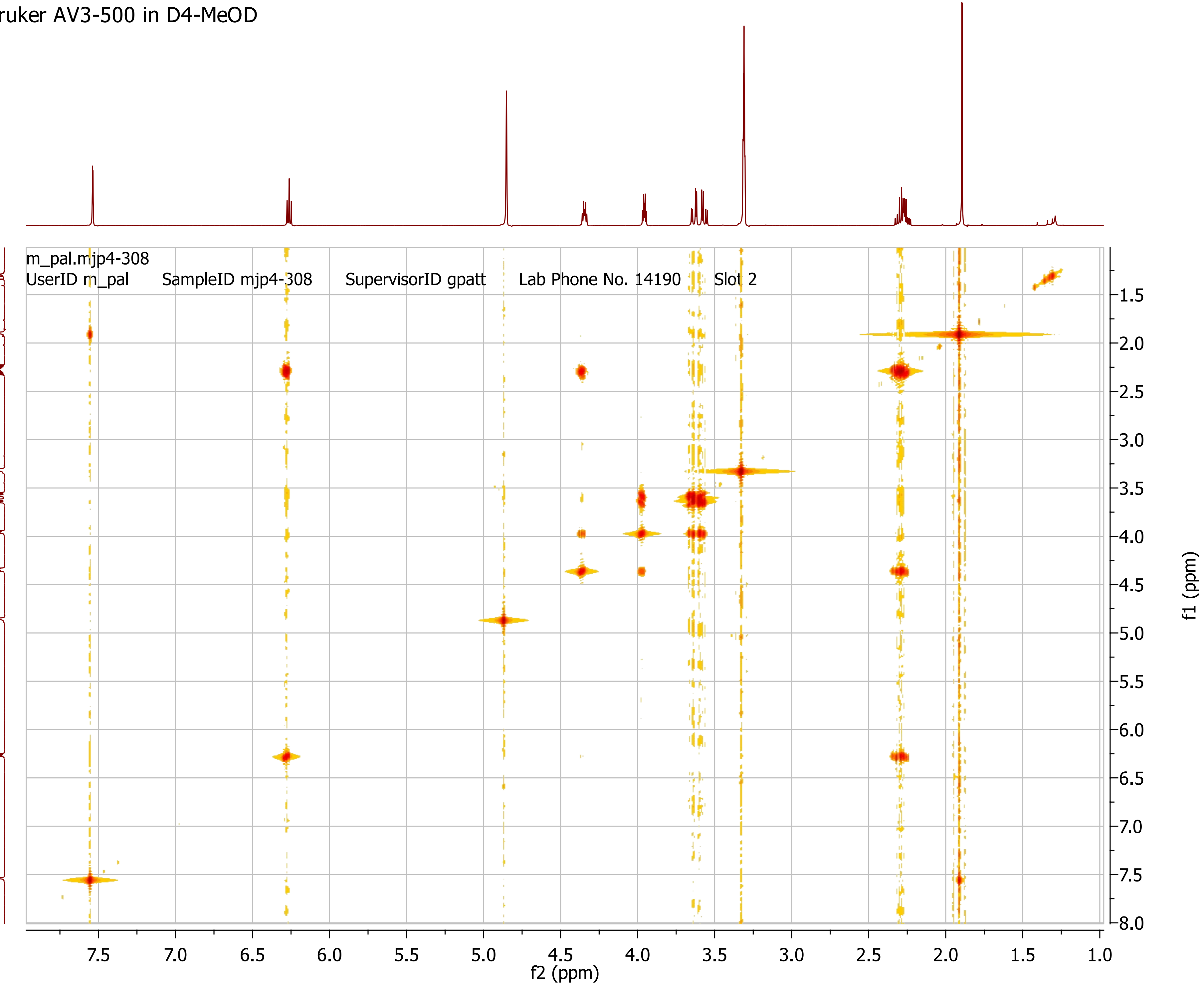

1H-13C HSQC recorded on Bruker AV3-500 in D4-MeOD

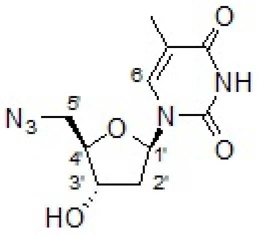

4

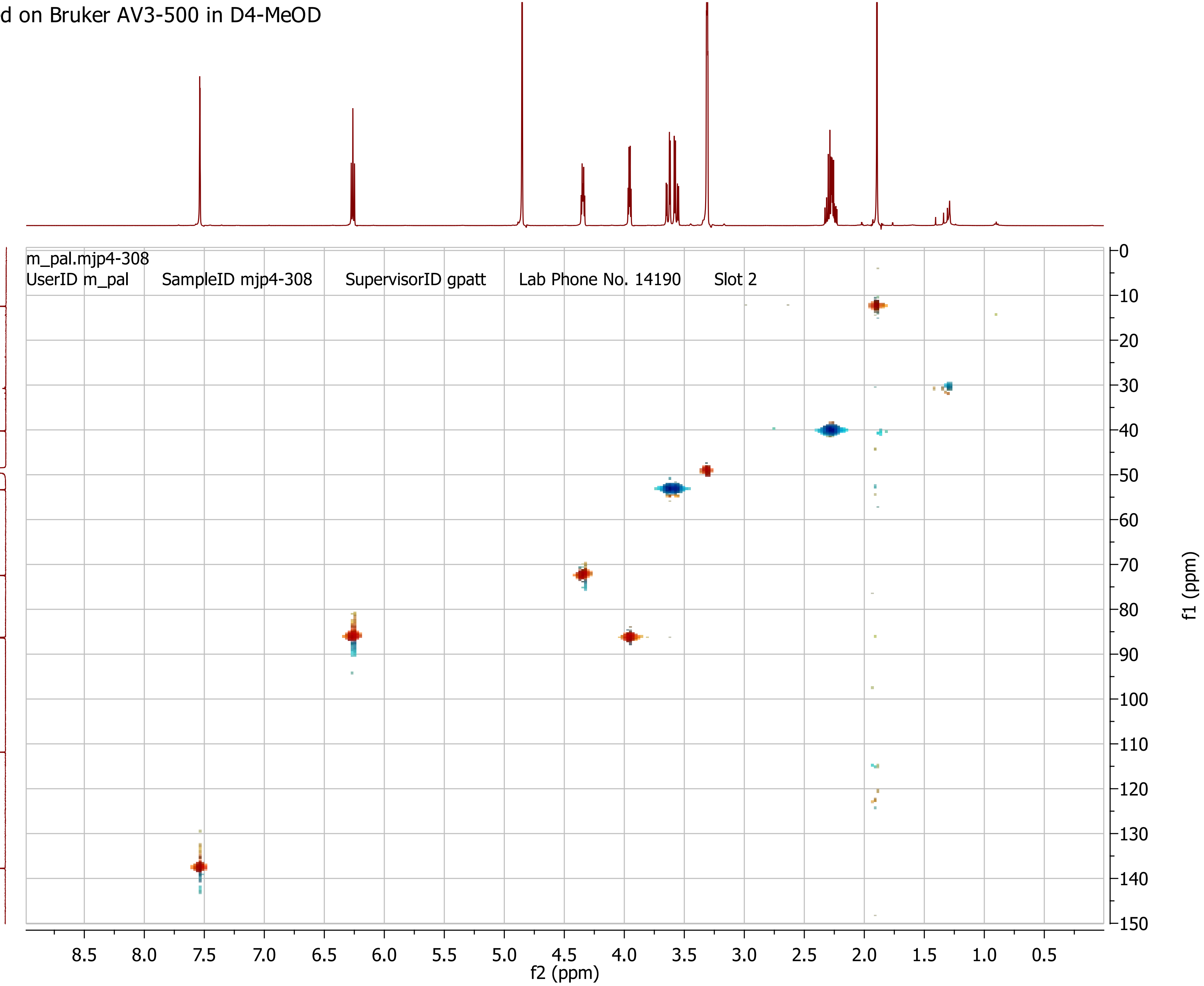

m_pal.MJP4-306
Slot No. 57 Sample ID MJP 4-306 col CDCl3 SupervisorID gpatt Lab Phone No. x UserID m_pal

Recorded on a Bruker DPX400 in $CDCl_3$
^{1}H NMR recorded at 400 MHz

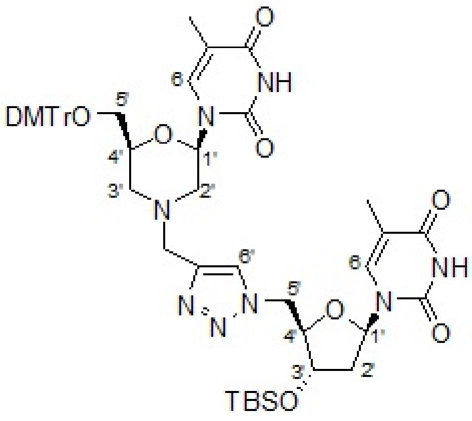

11

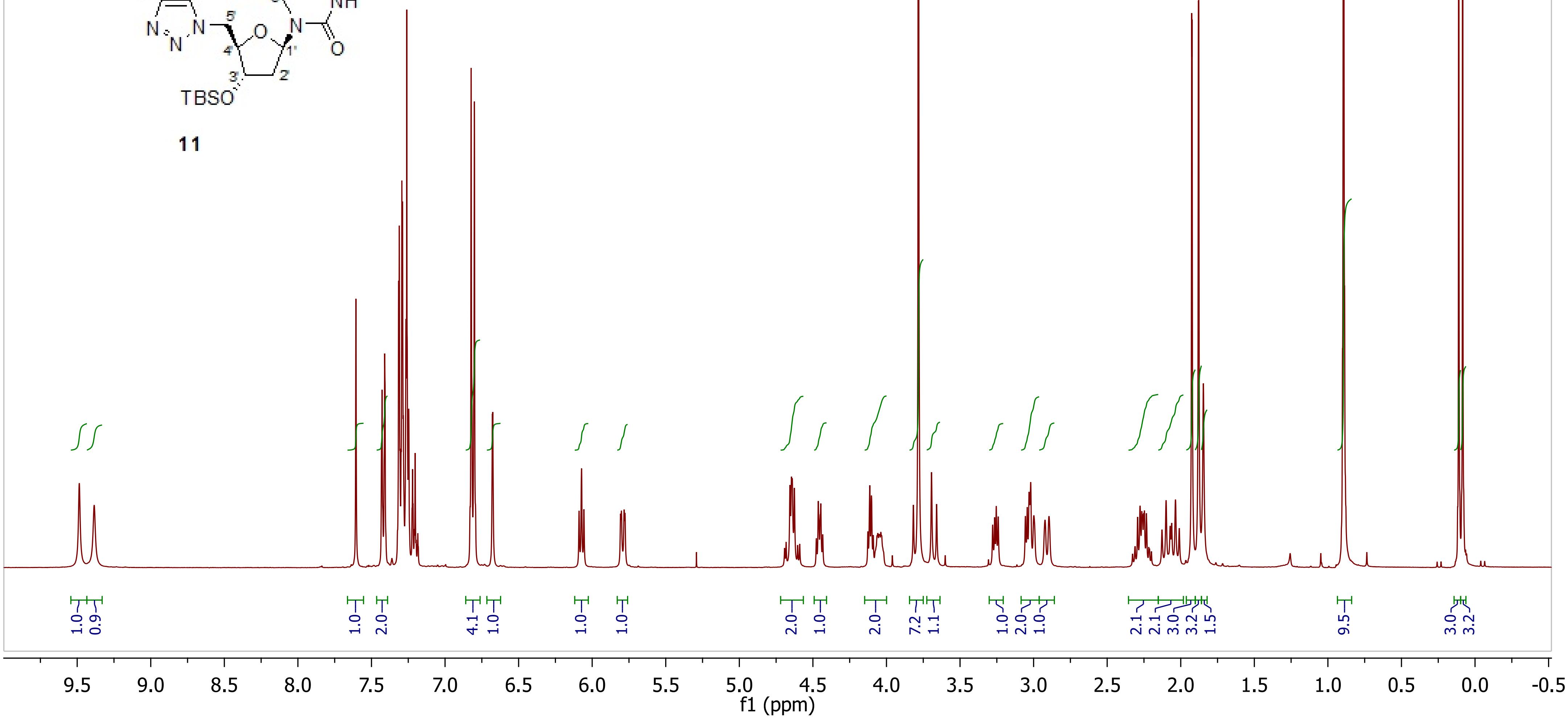

m_pal.MJP4-306
Slot No. 57 Sample ID MJP 4-306 col CDCl3 SupervisorID gpatt Lab Phone No. x UserID m_pal

Recorded on a Bruker DPX400 in $CDCl_3$
^{13}C NMR recorded at 101 MHz

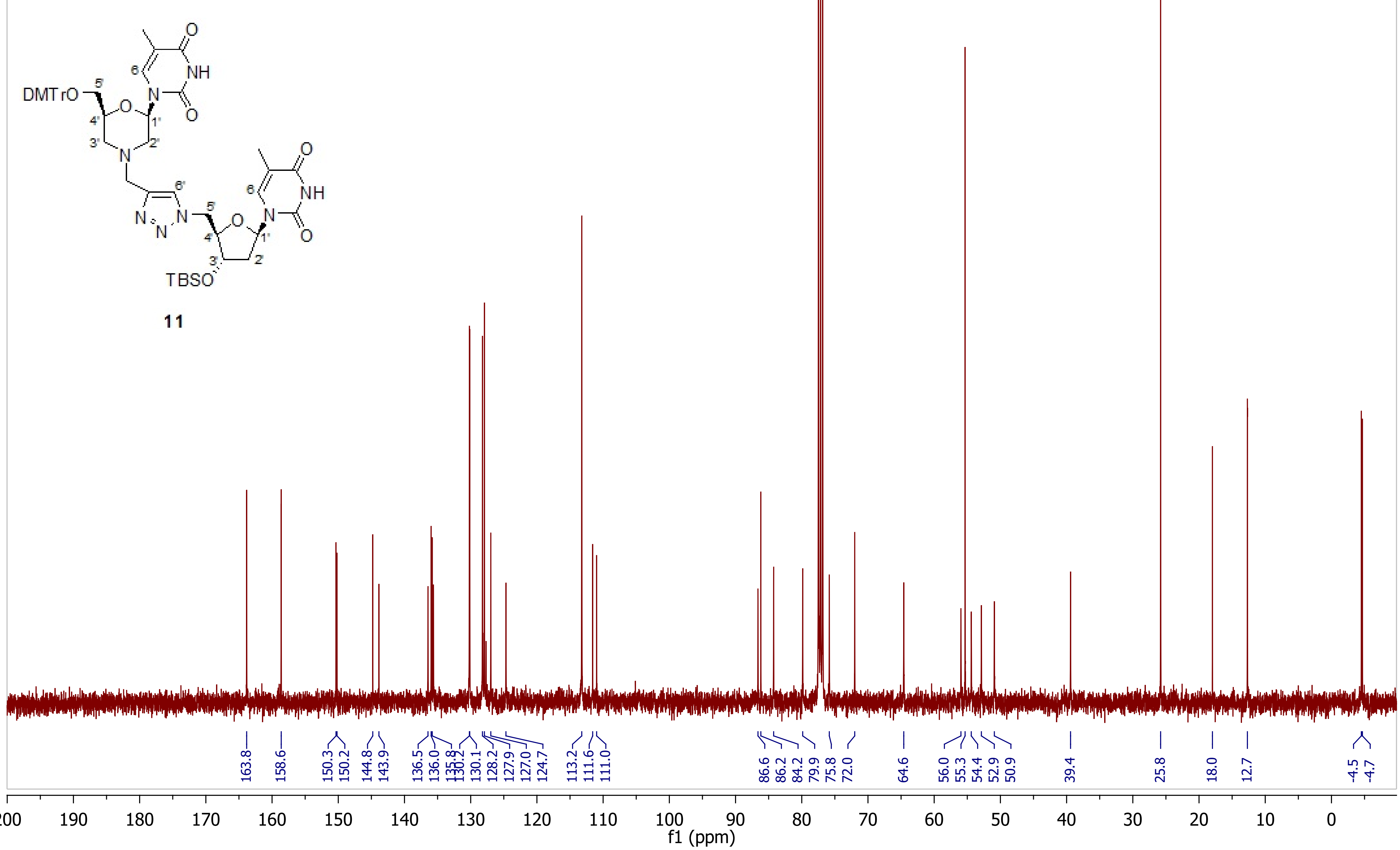

m_pal.MJP4-306
Slot No. 57 Sample ID MJP 4-306 col CDCl3 SupervisorID gpatt Lab Phone No. x UserID m_pal

Recorded on a Bruker DPX400 in $CDCl_3$
DEPT135 NMR recorded at 101 MHz

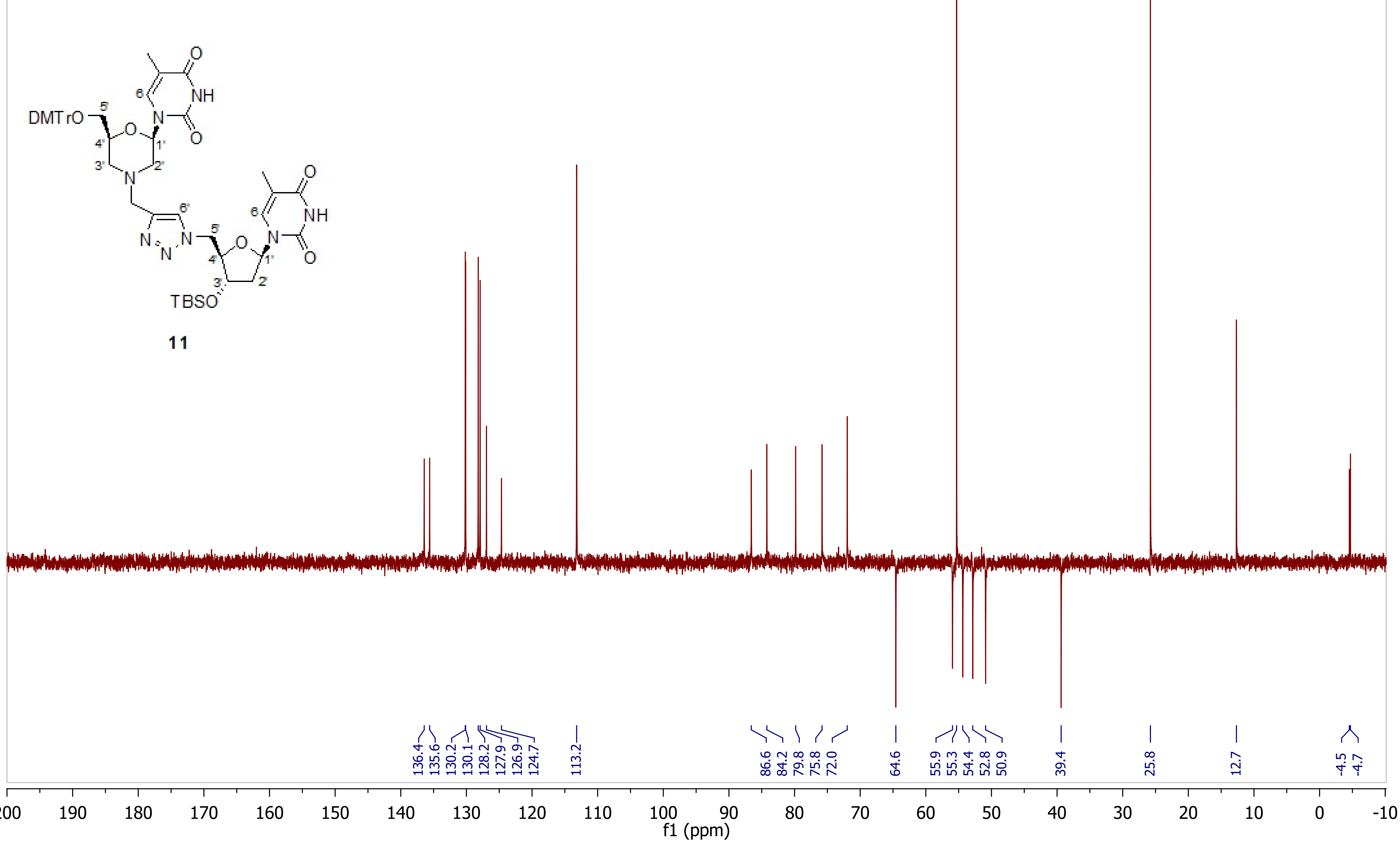

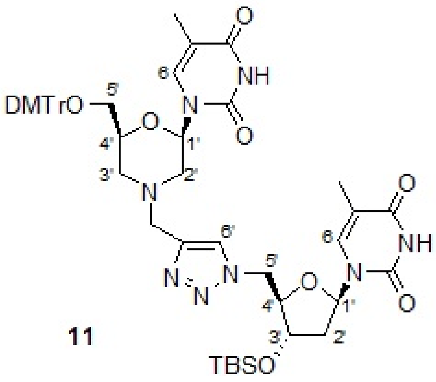
DMTrO
NH
O
N
TBSO
5'
4'
3'
2'
1'
6
6'

11

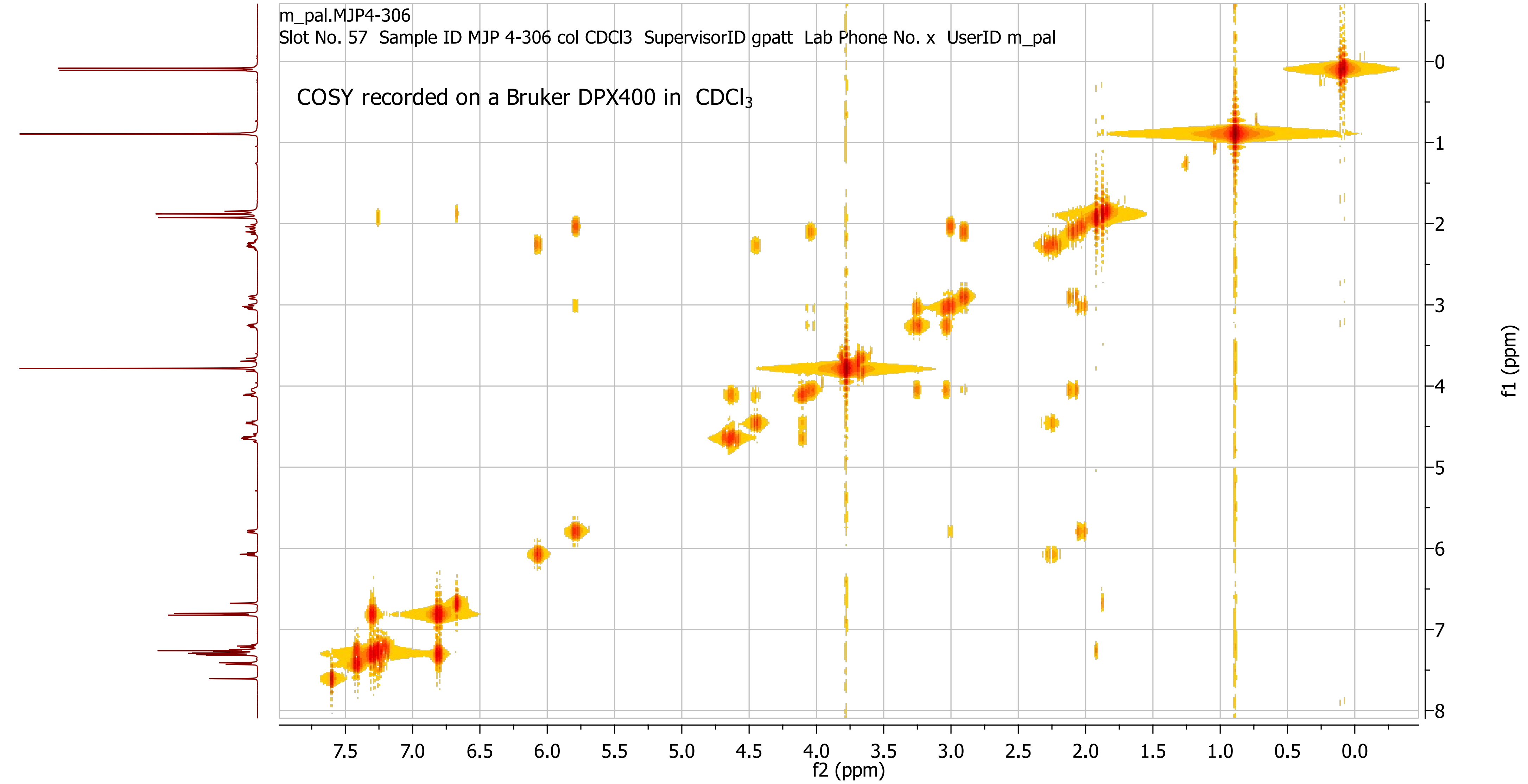
m_pal.MJP4-306
Slot No. 57 Sample ID MJP 4-306 col CDCl3 SupervisorID gpatt Lab Phone No. x UserID m_pal
COSY recorded on a Bruker DPX400 in CDCl3
f2 (ppm)
f1 (ppm)
7.5 7.0 6.5 6.0 5.5 5.0 4.5 4.0 3.5 3.0 2.5 2.0 1.5 1.0 0.5 0.0
0 1 2 3 4 5 6 7 8

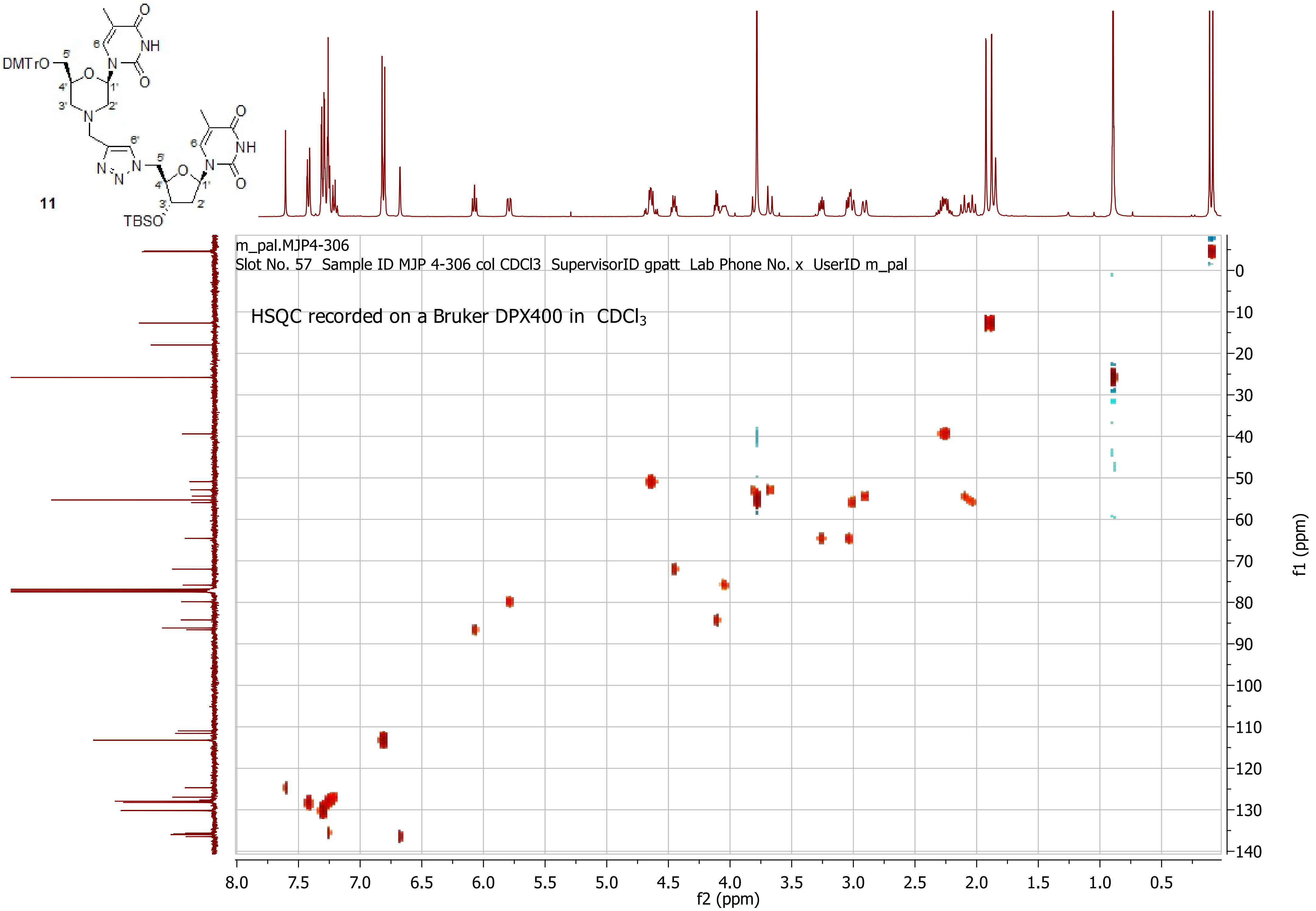

DMTrO
TBSO
11
m_pal.MJP4-306
Slot No. 57 Sample ID MJP 4-306 col CDCl3 SupervisorID gpatt Lab Phone No. x UserID m_pal
HSQC recorded on a Bruker DPX400 in CDCl$_3$
f2 (ppm)
f1 (ppm)

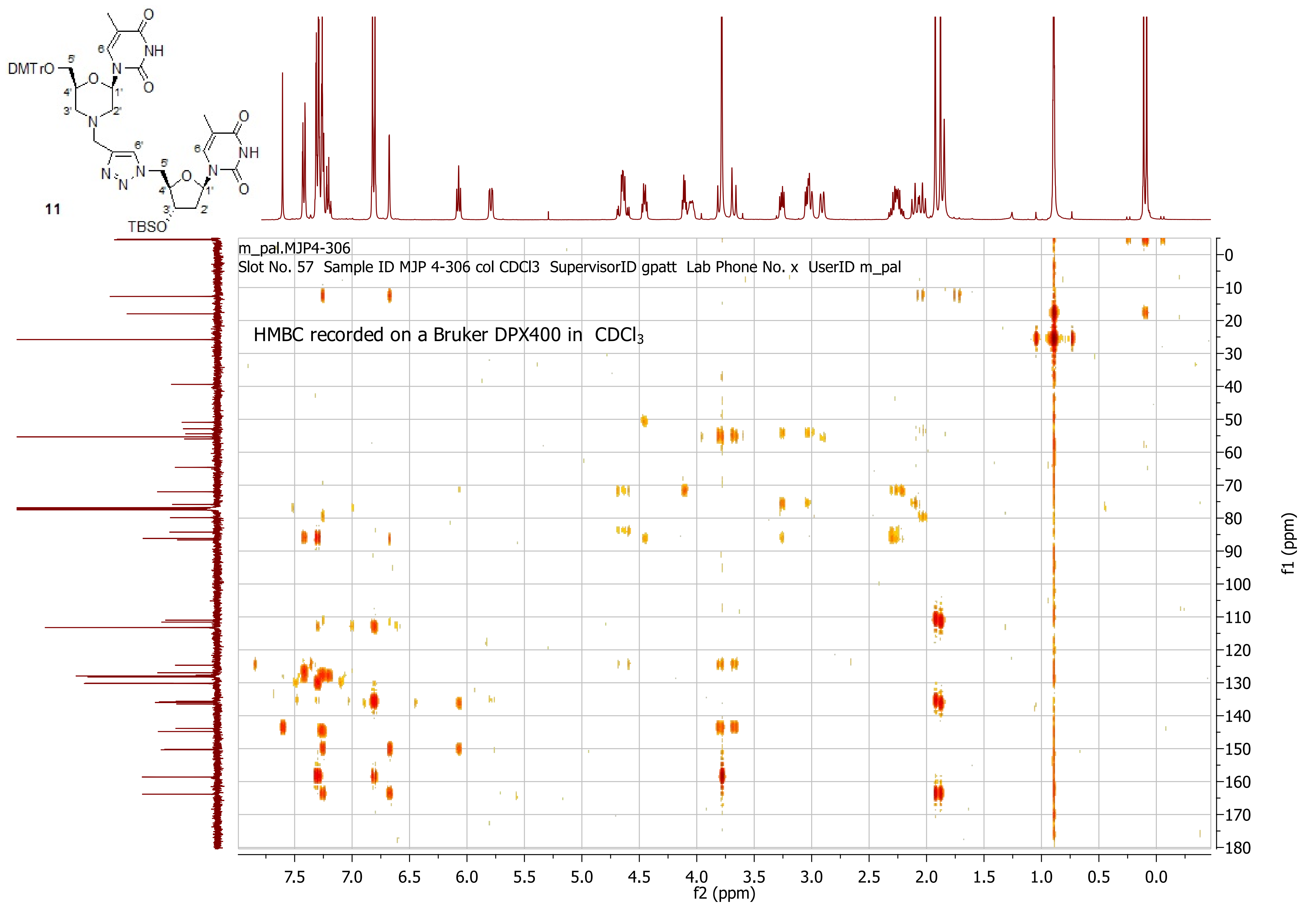

DMTrO
NH
N
O
11
TBSO
m_pal.MJP4-306
Slot No. 57 Sample ID MJP 4-306 col CDCl3 SupervisorID gpatt Lab Phone No. x UserID m_pal
HMBC recorded on a Bruker DPX400 in CDCl3
f2 (ppm)
f1 (ppm)

m_pal.mjp4-309
UserID m_pal SampleID mjp4-309 SupervisorID gpatt Lab Phone No. 14190 Slot 10

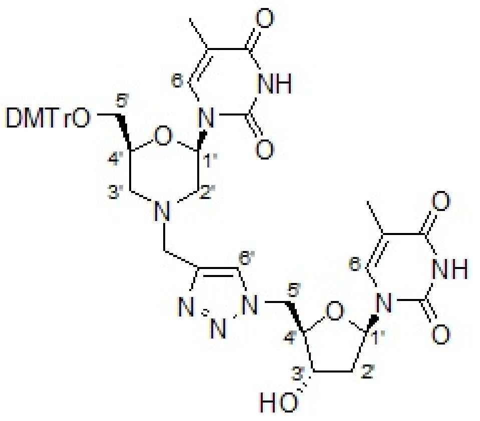

12

Recorded on a Bruker AV3-500 in $CDCl_3$
^1H NMR recorded at 500 MHz

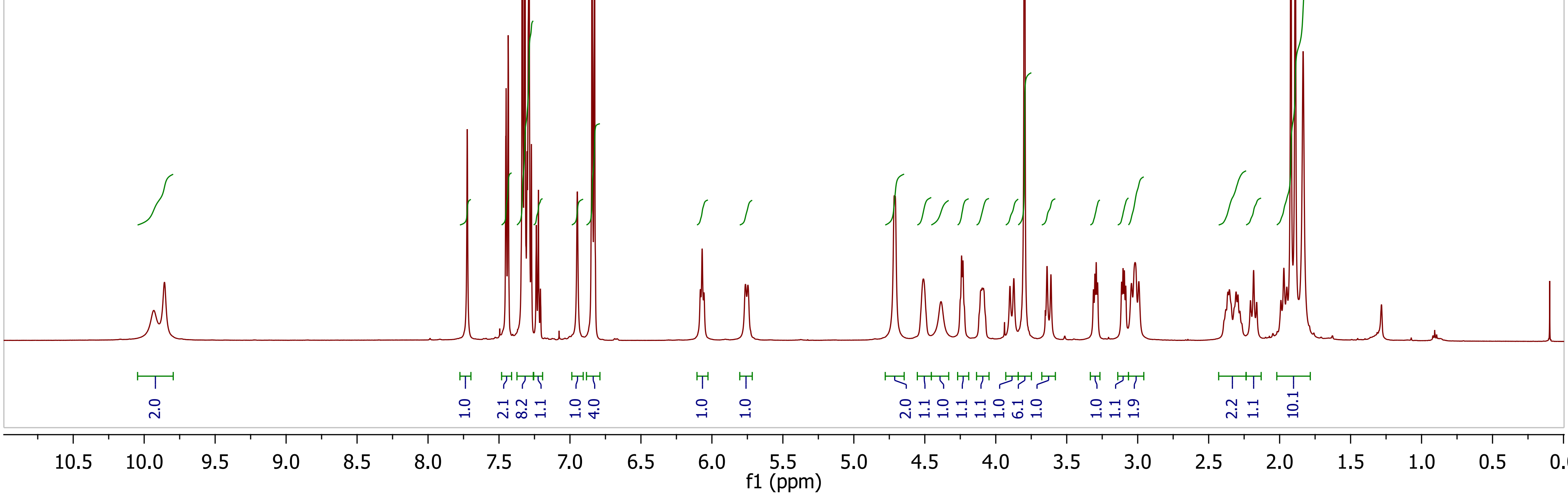

m_pal.mjp4-309
UserID m_pal SampleID mjp4-309 SupervisorID gpatt Lab Phone No. 14190 Slot 10

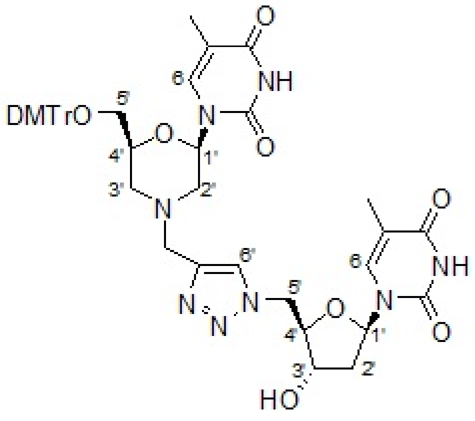

12

Recorded on a Bruker AV3-500 in $CDCl_3$
^{13}C NMR recorded at 126 MHz

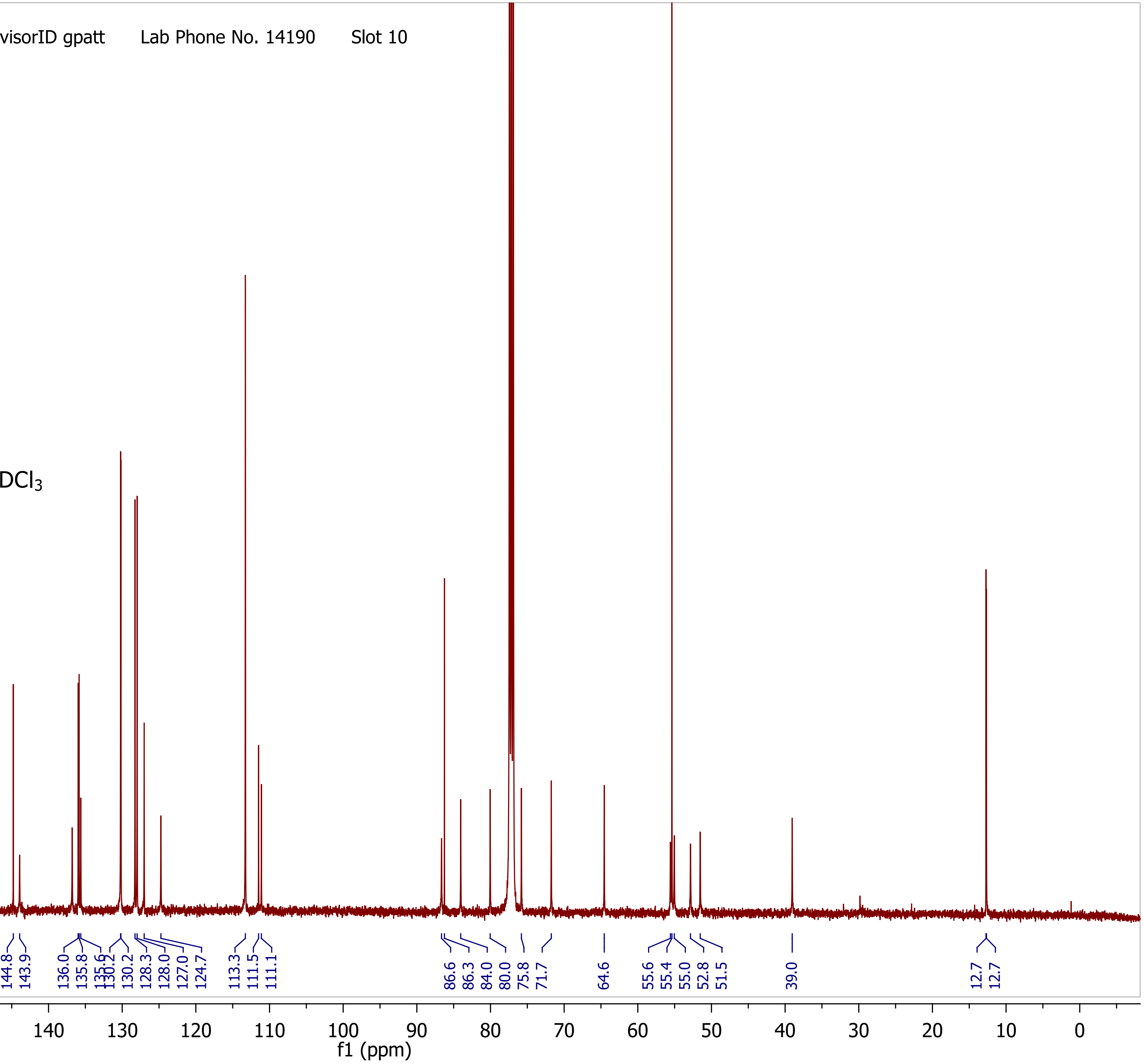

m_pal.mjp4-309

UserID m_pal SampleID mjp4-309 SupervisorID gpatt Lab Phone No. 14190 Slot 10

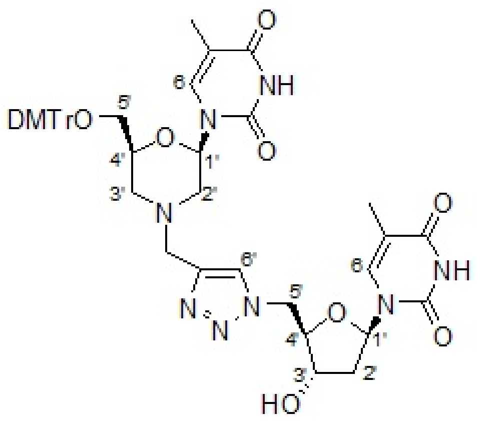

12

Recorded on a Bruker AV3-500 in $CDCl_3$

^{13}C NMR recorded at 126 MHz

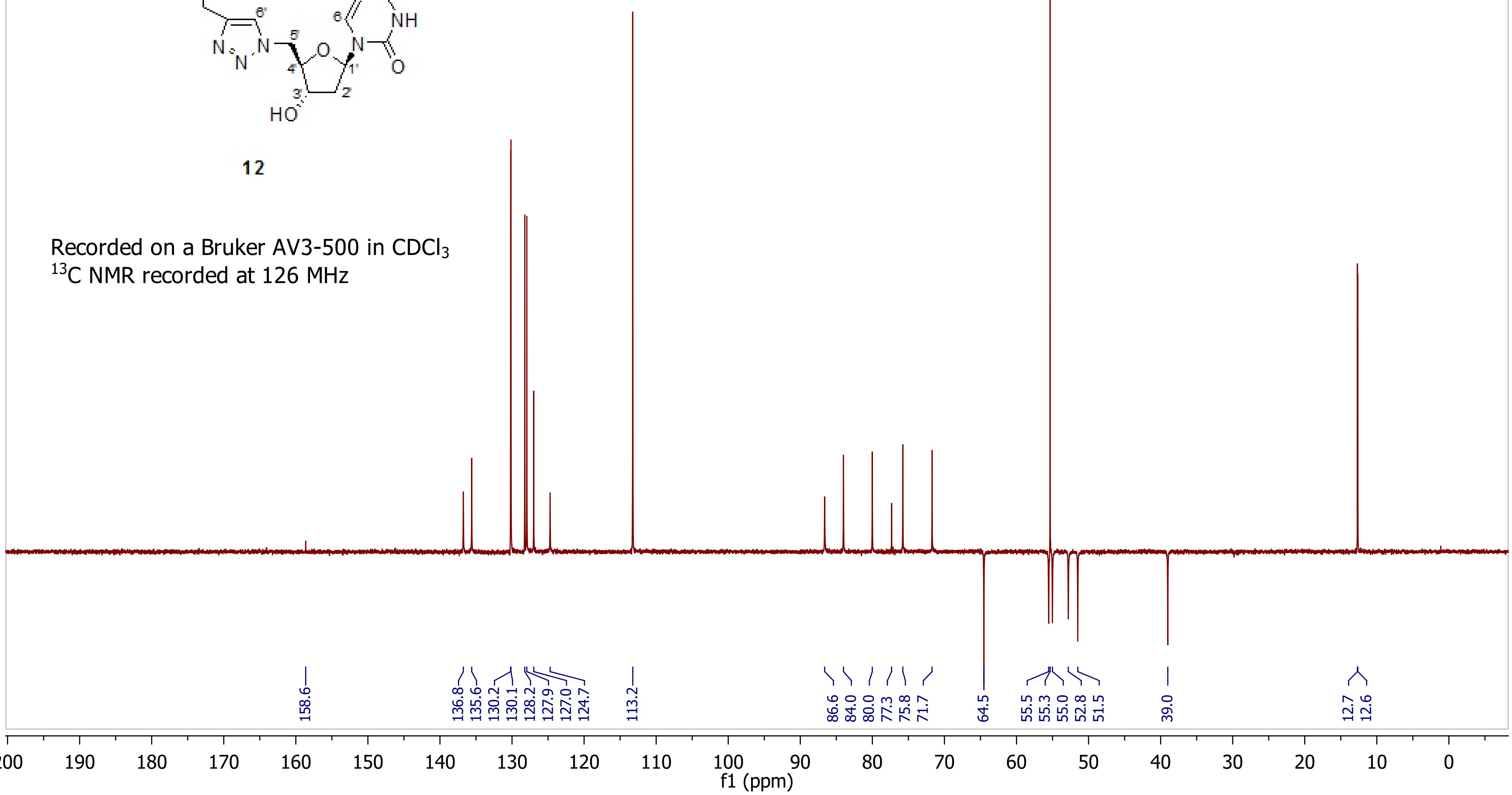

HSQC recorded on a Bruker AV3-500 in $CDCl_3$

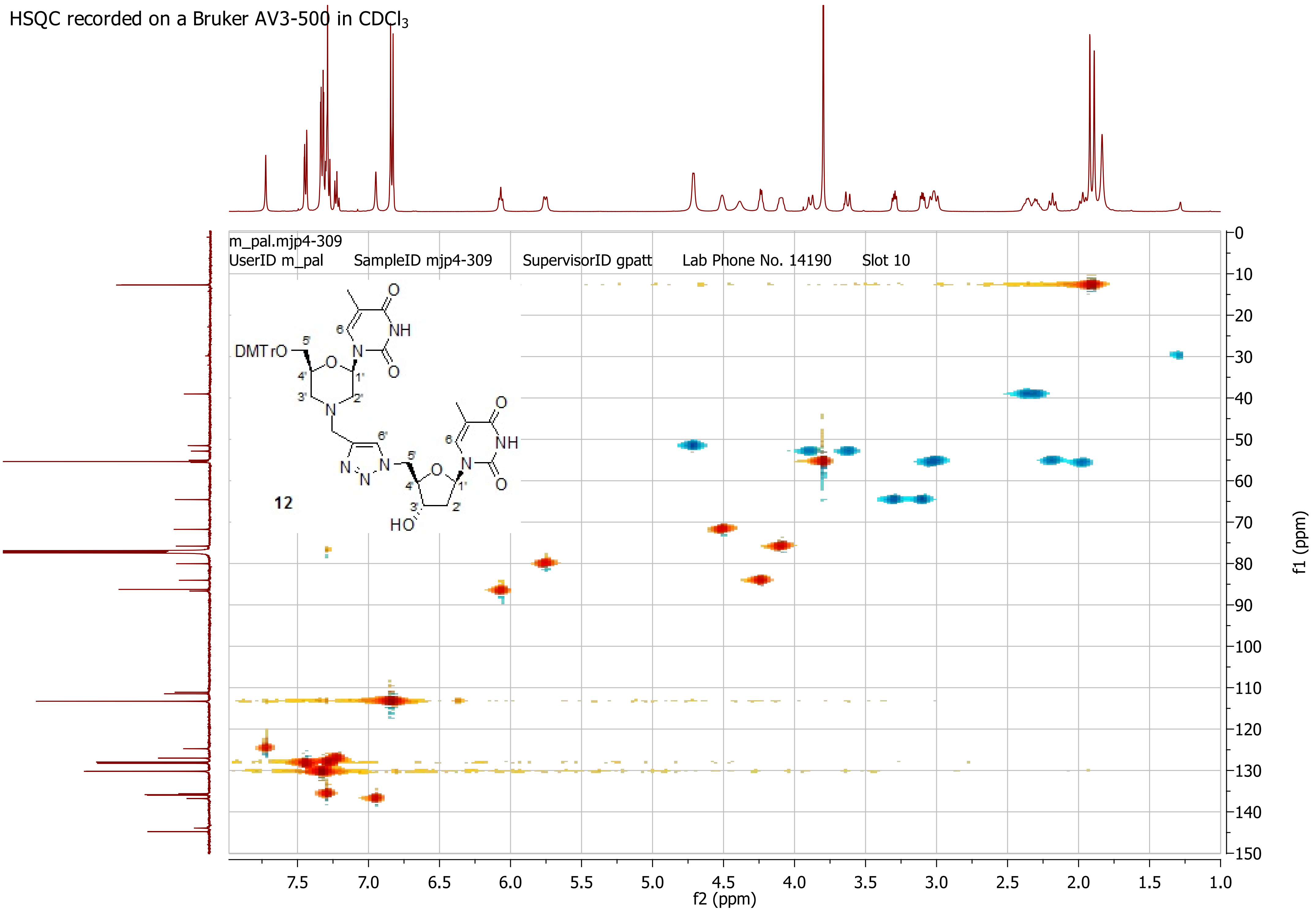

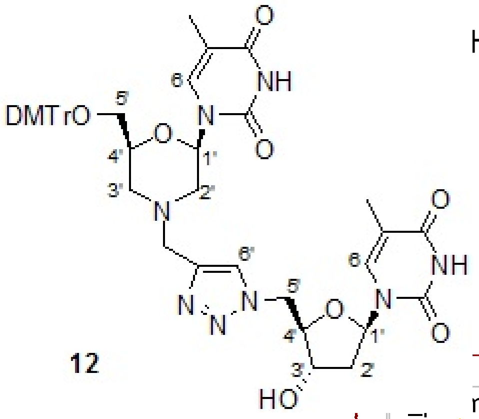

12

HMBC recorded on a Bruker AV3-500 in $CDCl_3$

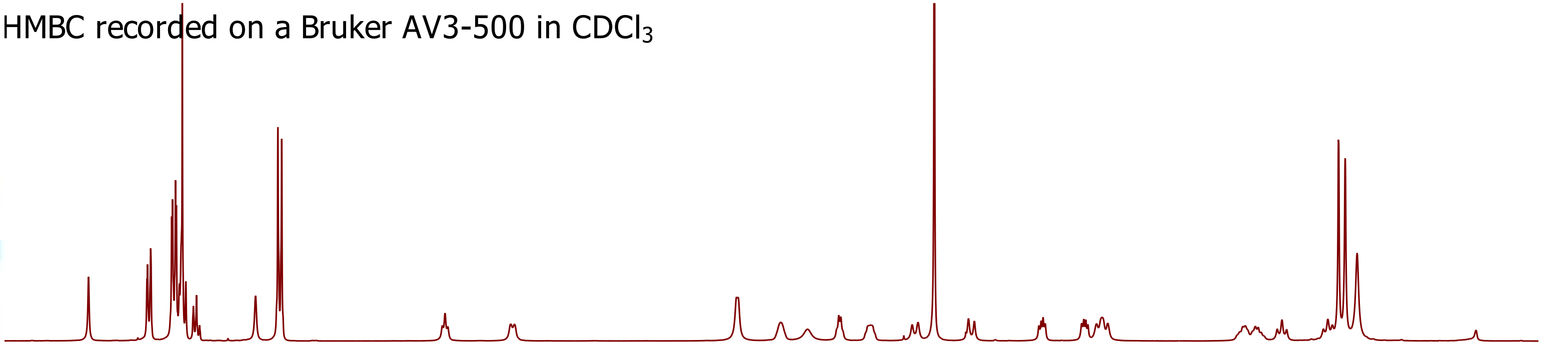

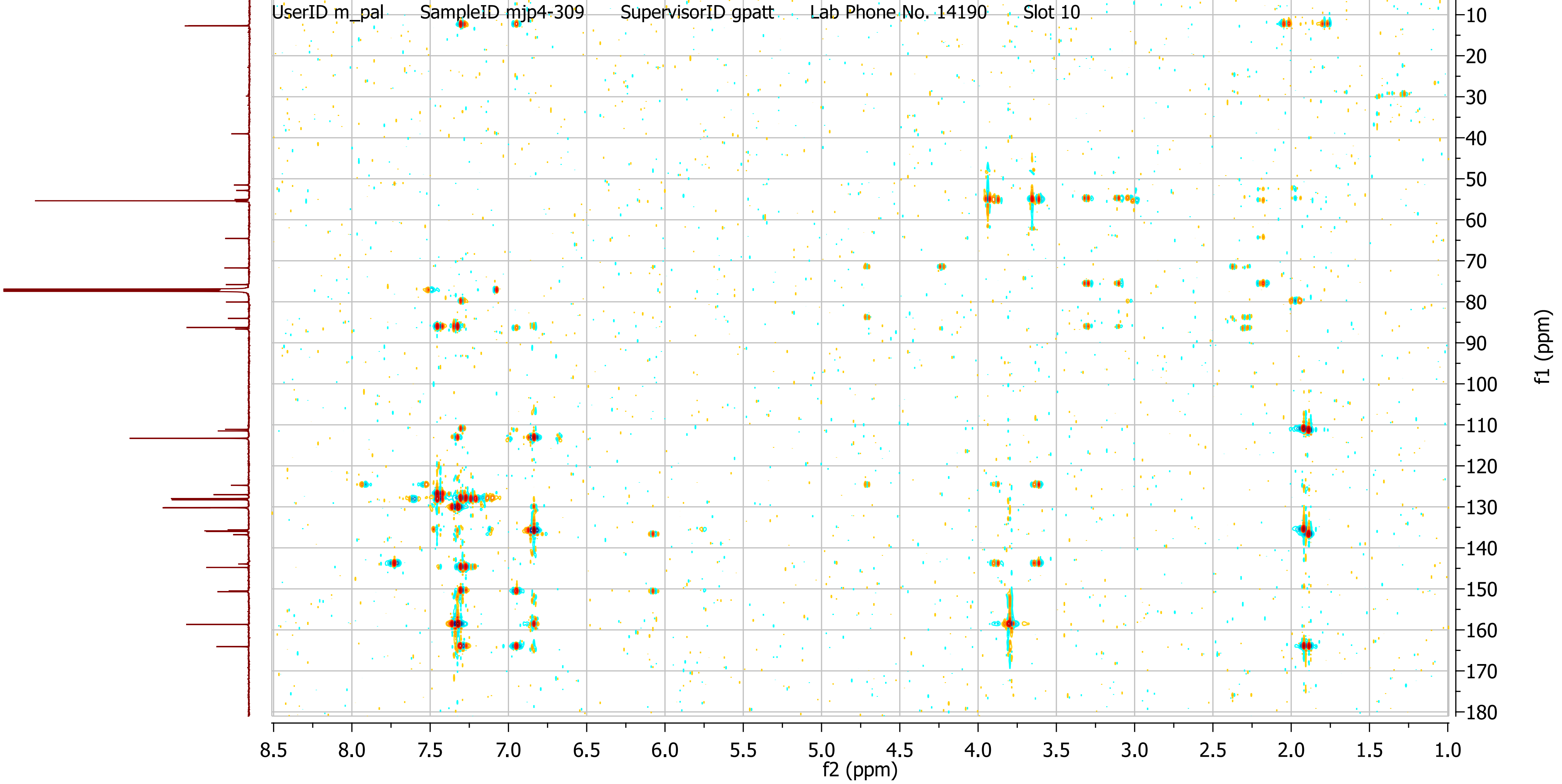

m_pal.4-334
UserID m_pal SampleID 4-334 a3a5 SupervisorID gpatt Lab Phone No. 14190 Slot 1

Recorded on a Bruker AV3-500 in $CDCl_3$
^1H NMR recorded at 500 MHz

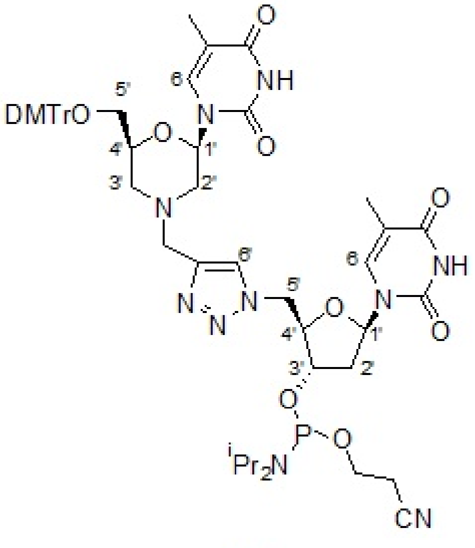

13

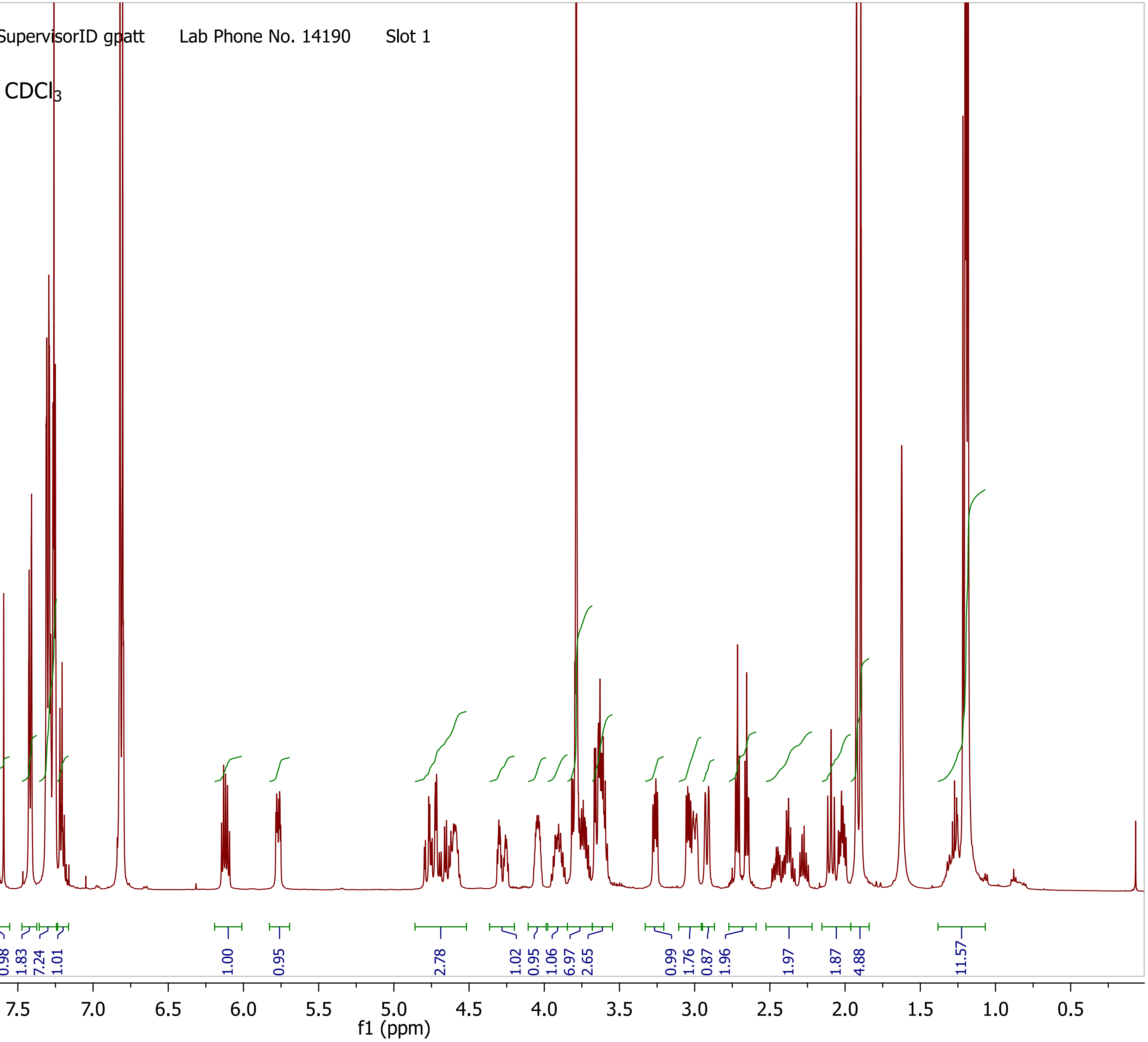

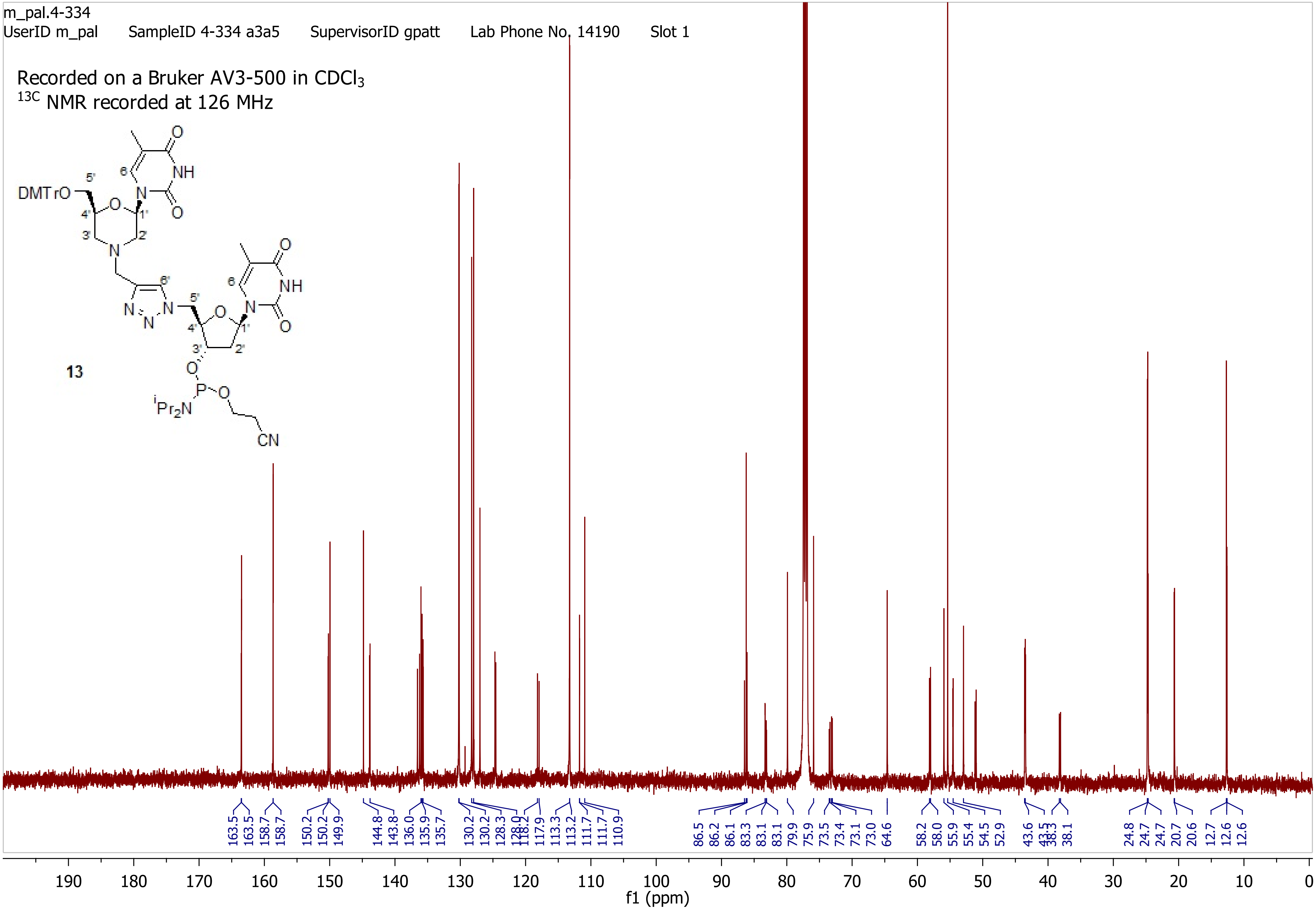
m_pal.4-334
UserID m_pal SampleID 4-334 a3a5 SupervisorID gpatt Lab Phone No. 14190 Slot 1
Recorded on a Bruker AV3-500 in CDCl3
13C NMR recorded at 126 MHz
DMTrO
NH
O
N
iPr2N
P
CN
13
163.5 163.5 158.7 158.7 150.2 150.2 149.9 144.8 143.8 136.0 135.9 135.7 130.2 130.2 128.3 128.0 118.2 117.9 113.3 113.2 111.7 111.7 110.9 86.5 86.2 86.1 83.3 83.1 83.1 79.9 75.9 73.5 73.4 73.1 73.0 64.6 58.2 58.0 55.9 55.4 54.5 52.9 43.6 43.5 38.3 38.1 24.8 24.7 24.7 20.7 20.6 12.7 12.6 12.6
190 180 170 160 150 140 130 120 110 100 90 80 70 60 50 40 30 20 10 0
f1 (ppm)

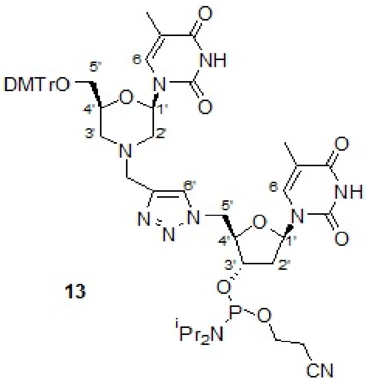
DMTrO
NH
N
O
N
N
NH
O
O
P
O
$^{i}Pr_2N$
CN

13

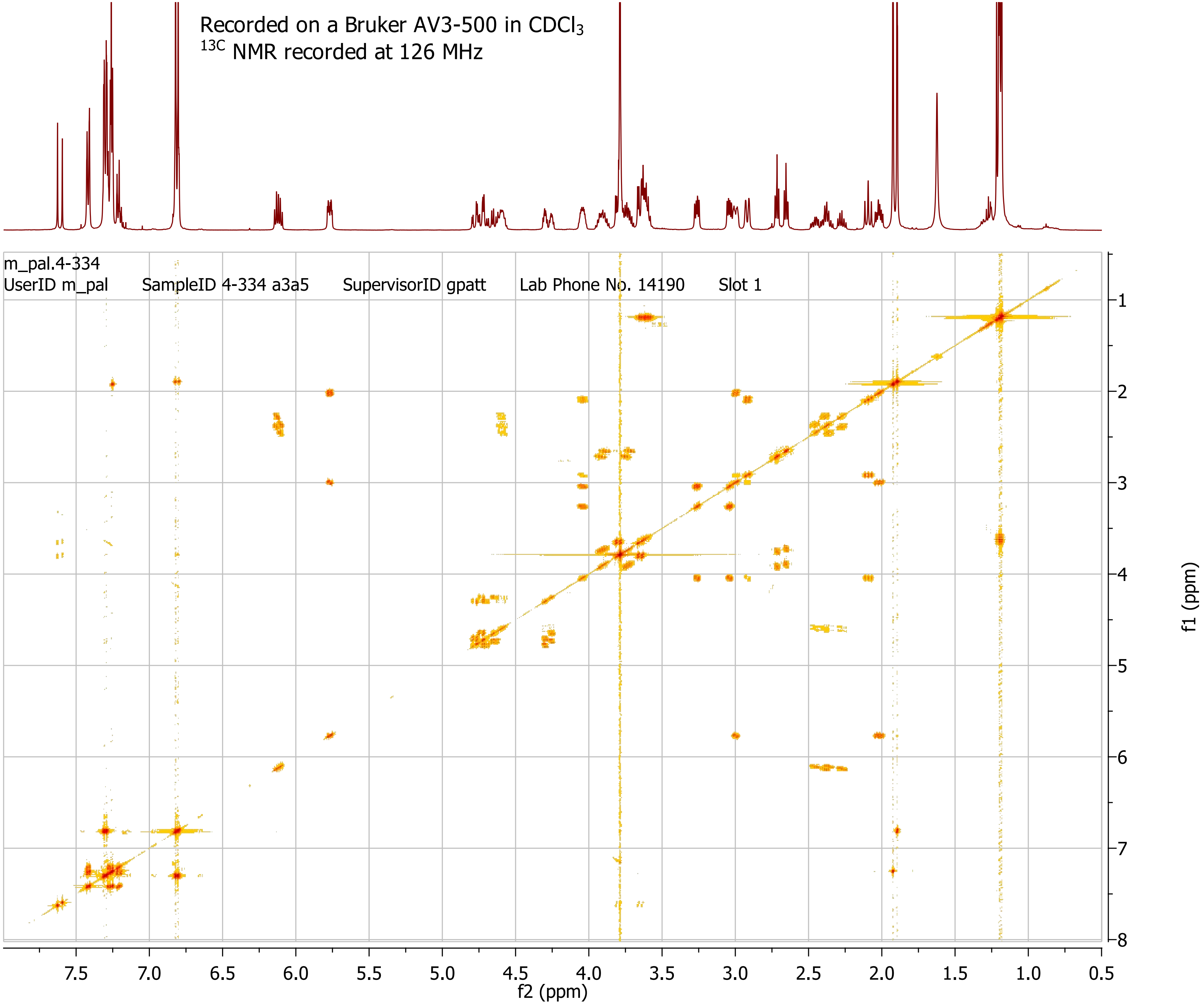
Recorded on a Bruker AV3-500 in $CDCl_3$
^{13}C NMR recorded at 126 MHz
m_pal.4-334
UserID m_pal SampleID 4-334 a3a5 SupervisorID gpatt Lab Phone No. 14190 Slot 1
f2 (ppm)
f1 (ppm)

Recorded on a Bruker AV3-500 in $CDCl_3$
^{13}C NMR recorded at 126 MHz

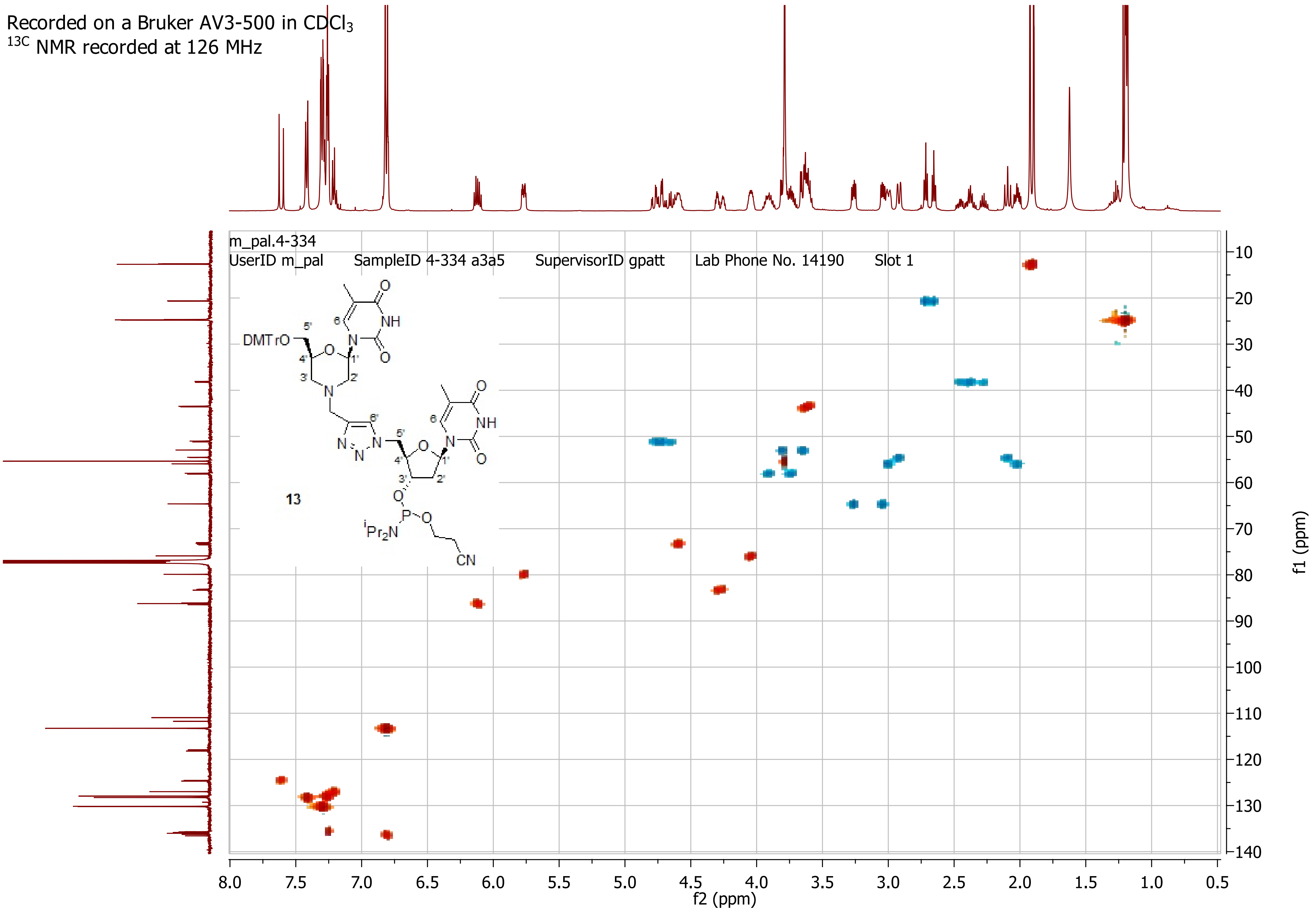

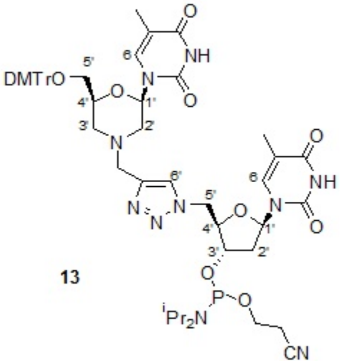
DMTrO
NH
O
N
N
NH
O
CN

13

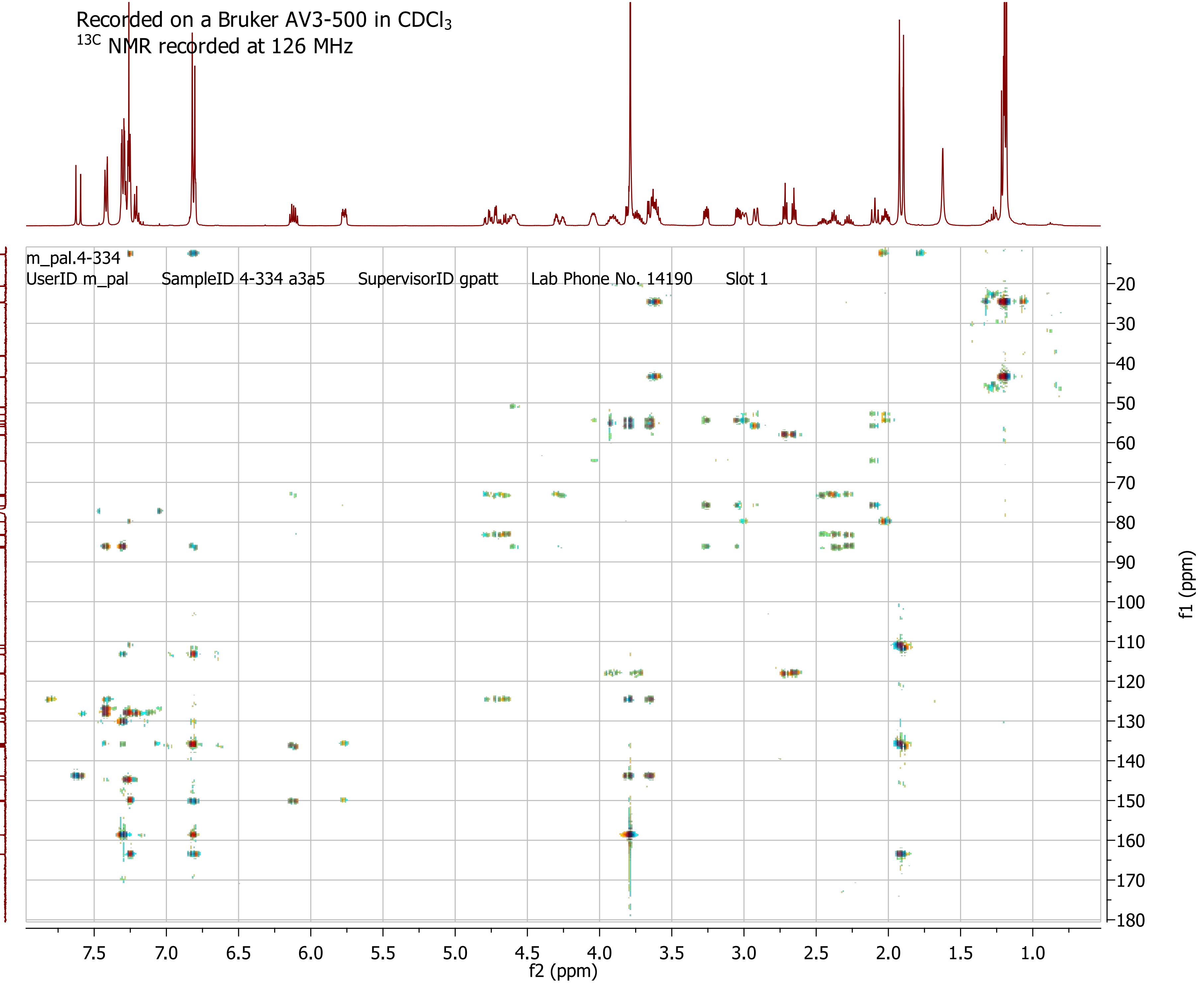
Recorded on a Bruker AV3-500 in CDCl3
13C NMR recorded at 126 MHz
m_pal.4-334
UserID m_pal SampleID 4-334 a3a5 SupervisorID gpatt Lab Phone No. 14190 Slot 1
f2 (ppm)
f1 (ppm)

m_pal.MJP4-334
Slot No. 44 Sample ID MJP 4-334 A3A5 CDCl3 SupervisorID gpatt Lab Phone No. x UserID m_pal

Recorded on a Bruker DPX400 in $CDCl_3$
^{31}P NMR recorded at 162 MHz

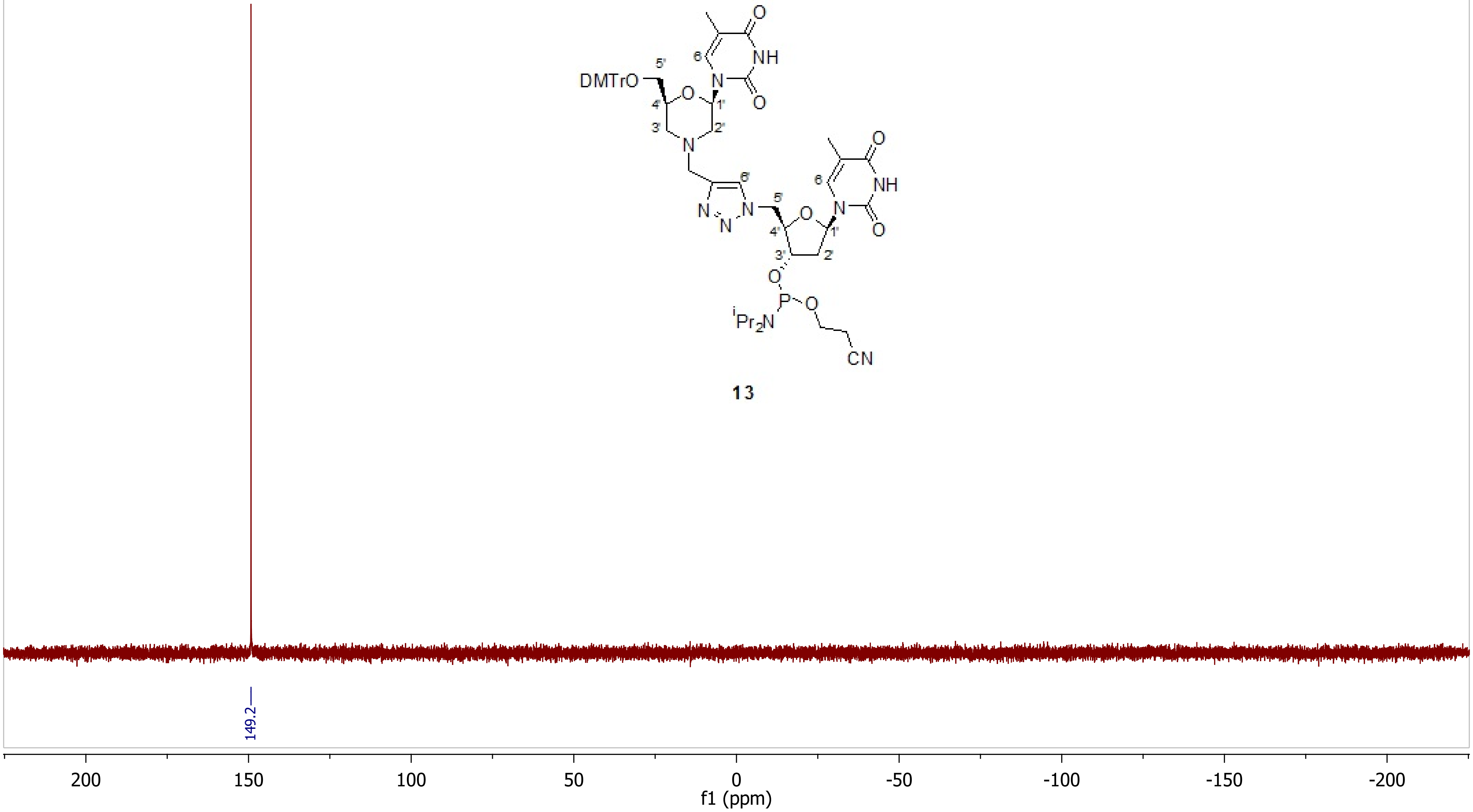

m_pal.MJP5-468
UserID m_pal SampleID MJP 5-469 col SupervisorID hayes Lab Phone No. 13536 Slot Number 38

Recorded on a Bruker AV-3-400 in $CDCl_3$
^1H NMR recorded at 400 MHz

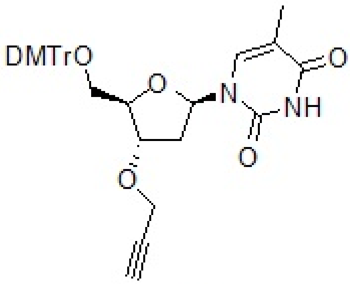

15

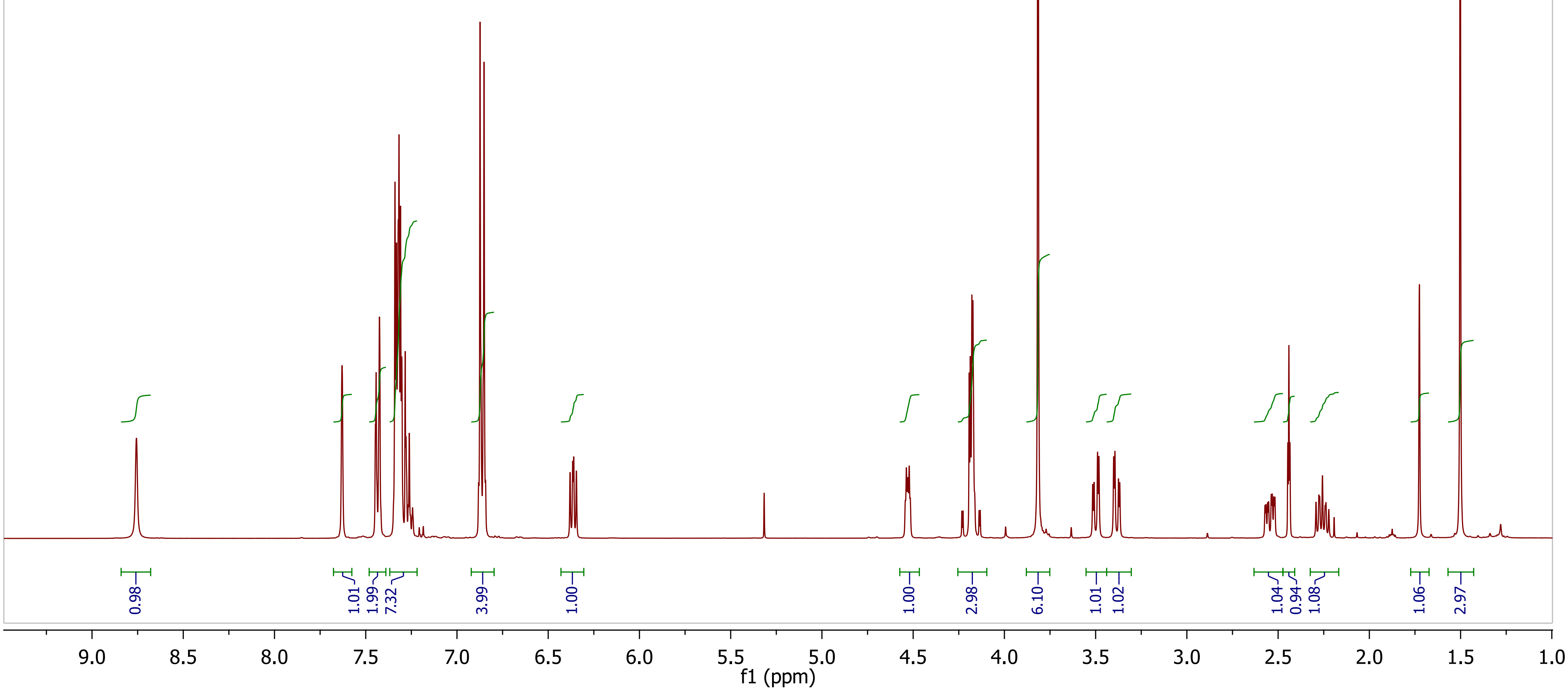

m_pal.MJP5-468
UserID m_pal SampleID MJP 5-469 col SupervisorID hayes Lab Phone No. 13536 Slot Number 38

Recorded on a Bruker AV-3-400 in $CDCl_3$
^{13}C NMR recorded at 101 MHz

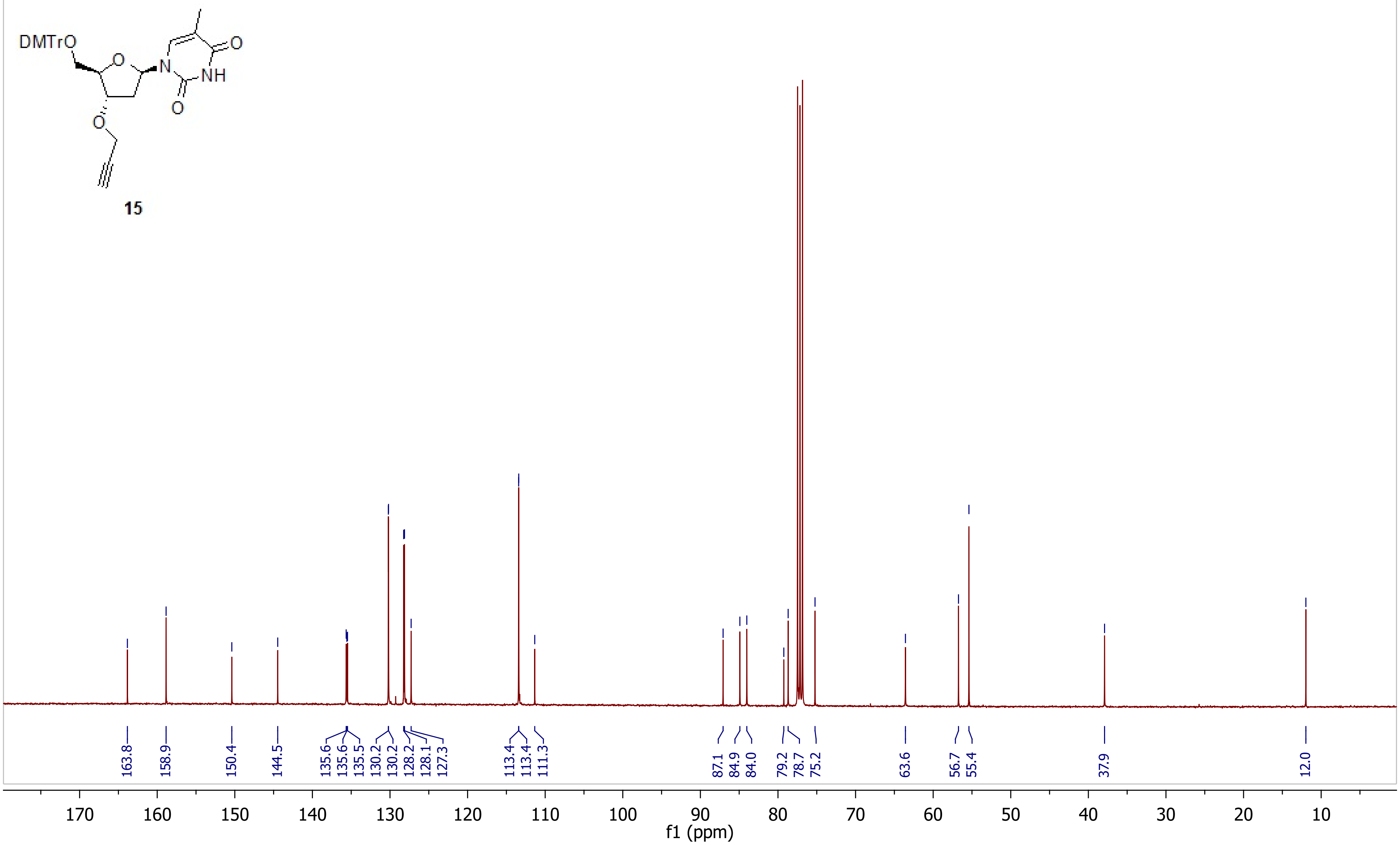

m_pal.MJP5-468
UserID m_pal SampleID MJP 5-469 col SupervisorID hayes Lab Phone No. 13536 Slot Number 38

Recorded on a Bruker AV-3-400 in $CDCl_3$
DEPT 135 NMR recorded at 101 MHz

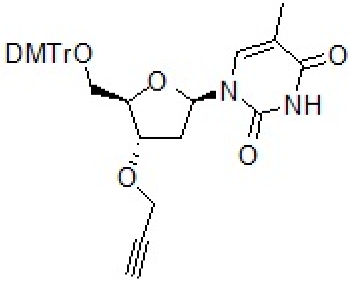

15

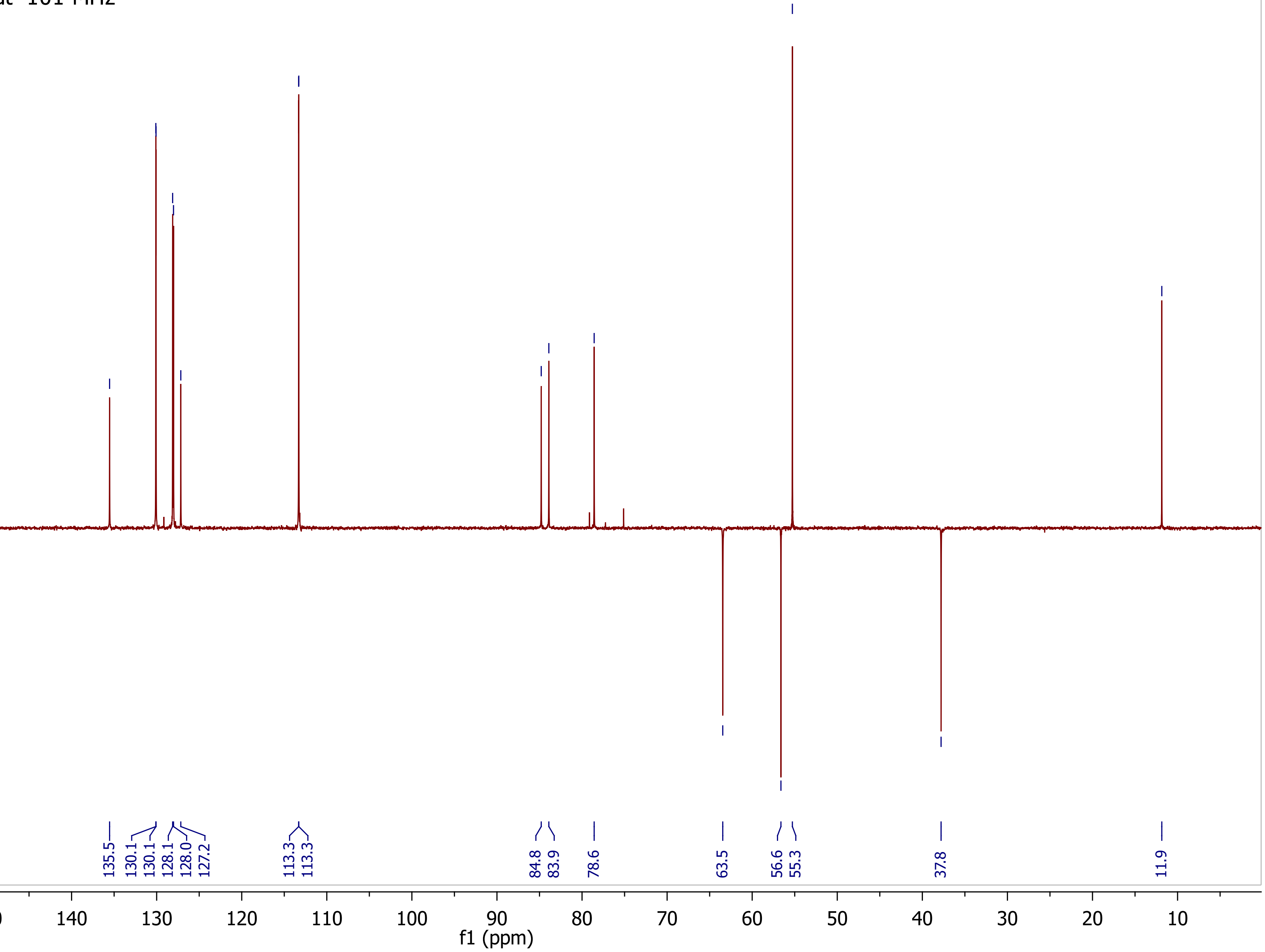

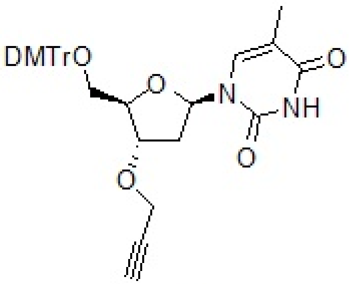

15

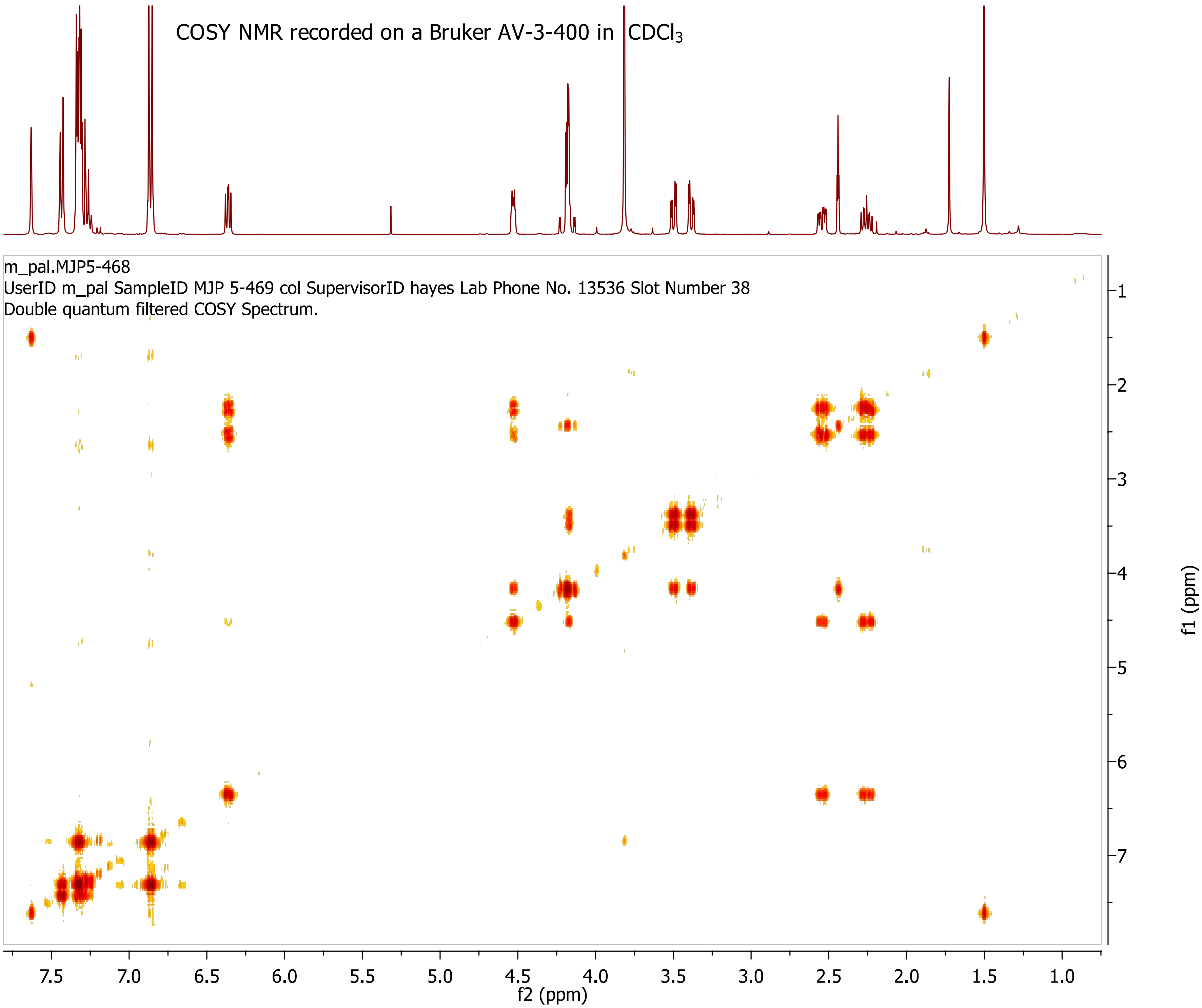

COSY NMR recorded on a Bruker AV-3-400 in CDCl3
m_pal.MJP5-468
UserID m_pal SampleID MJP 5-469 col SupervisorID hayes Lab Phone No. 13536 Slot Number 38
Double quantum filtered COSY Spectrum.
f2 (ppm)
f1 (ppm)

DEPT editted HSQC recorded on a Bruker AV-3-400 in $CDCl_3$

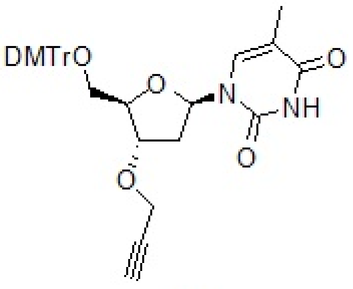

15

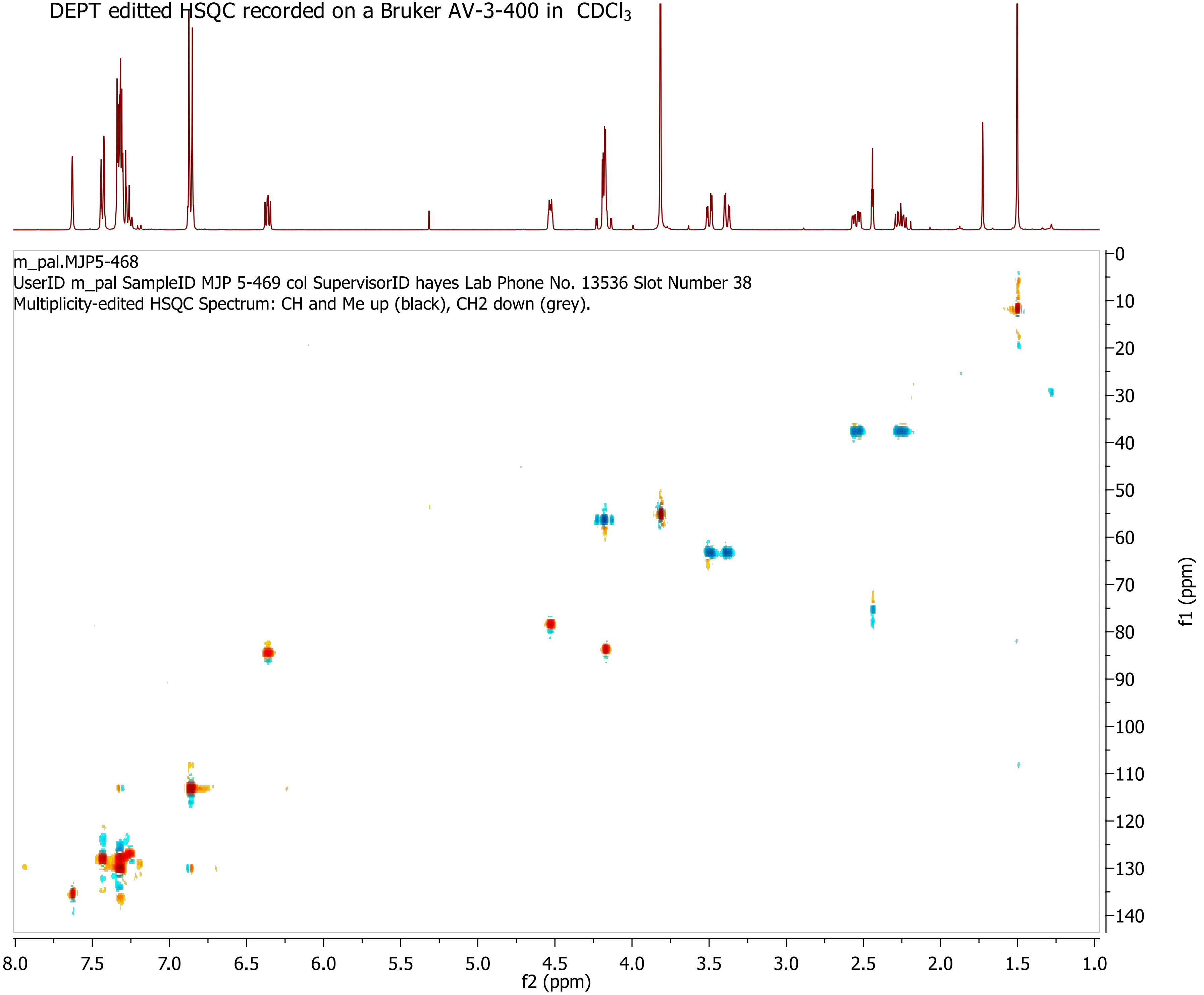

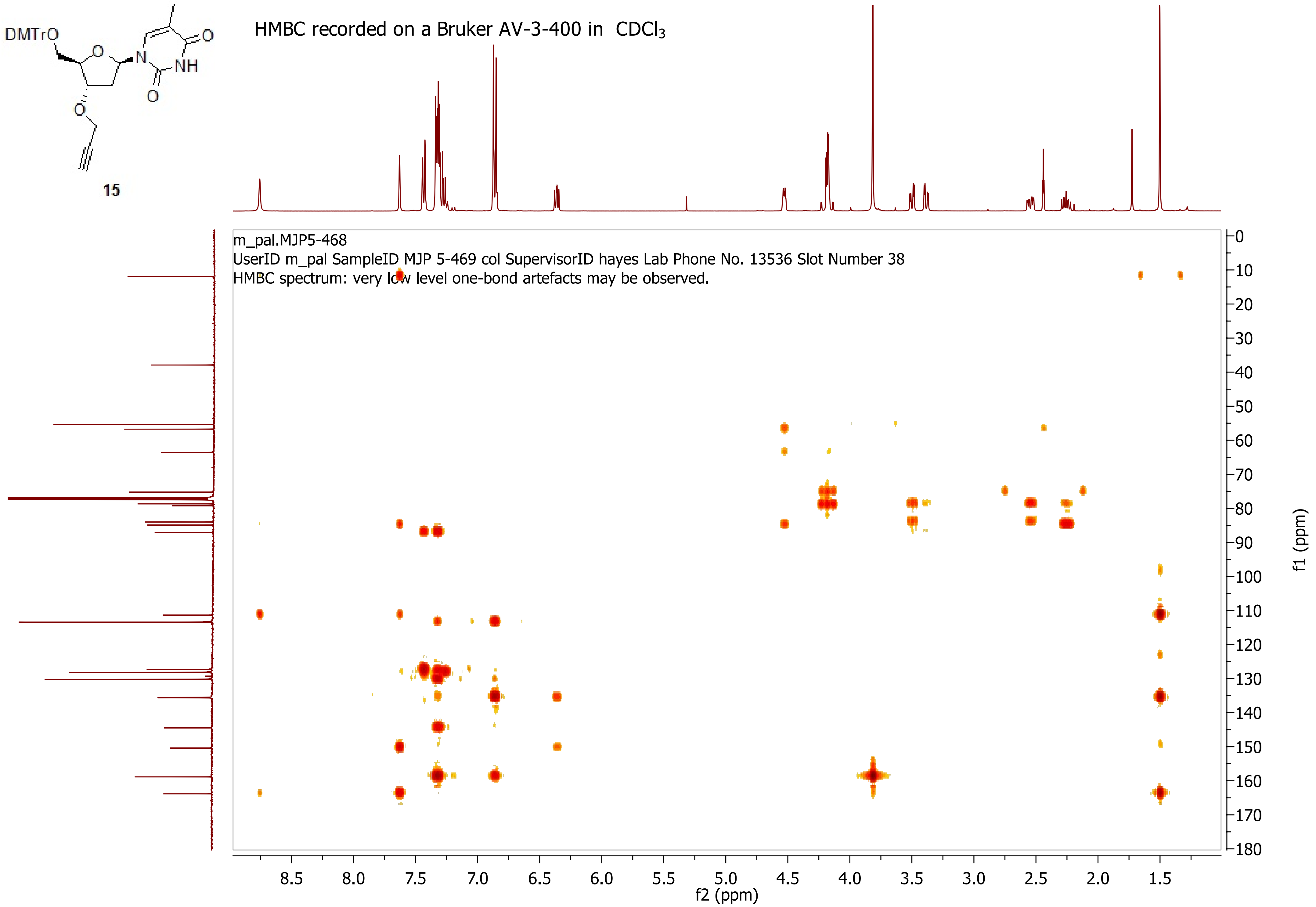
DMTrO
O
N
NH
O
O
O
15
HMBC recorded on a Bruker AV-3-400 in $CDCl_3$
m_pal.MJP5-468
UserID m_pal SampleID MJP 5-469 col SupervisorID hayes Lab Phone No. 13536 Slot Number 38
HMBC spectrum: very low level one-bond artefacts may be observed.
f2 (ppm)
f1 (ppm)

m_pal.MJP5-471
UserID m_pal SampleID MJP 5-471 - col - DMSO SupervisorID hayes Lab Phone No. 13536 Slot Number 13

Recorded on a Bruker AV-3-400 in d6-DMSO
^{1}H NMR recorded at 400 MHz

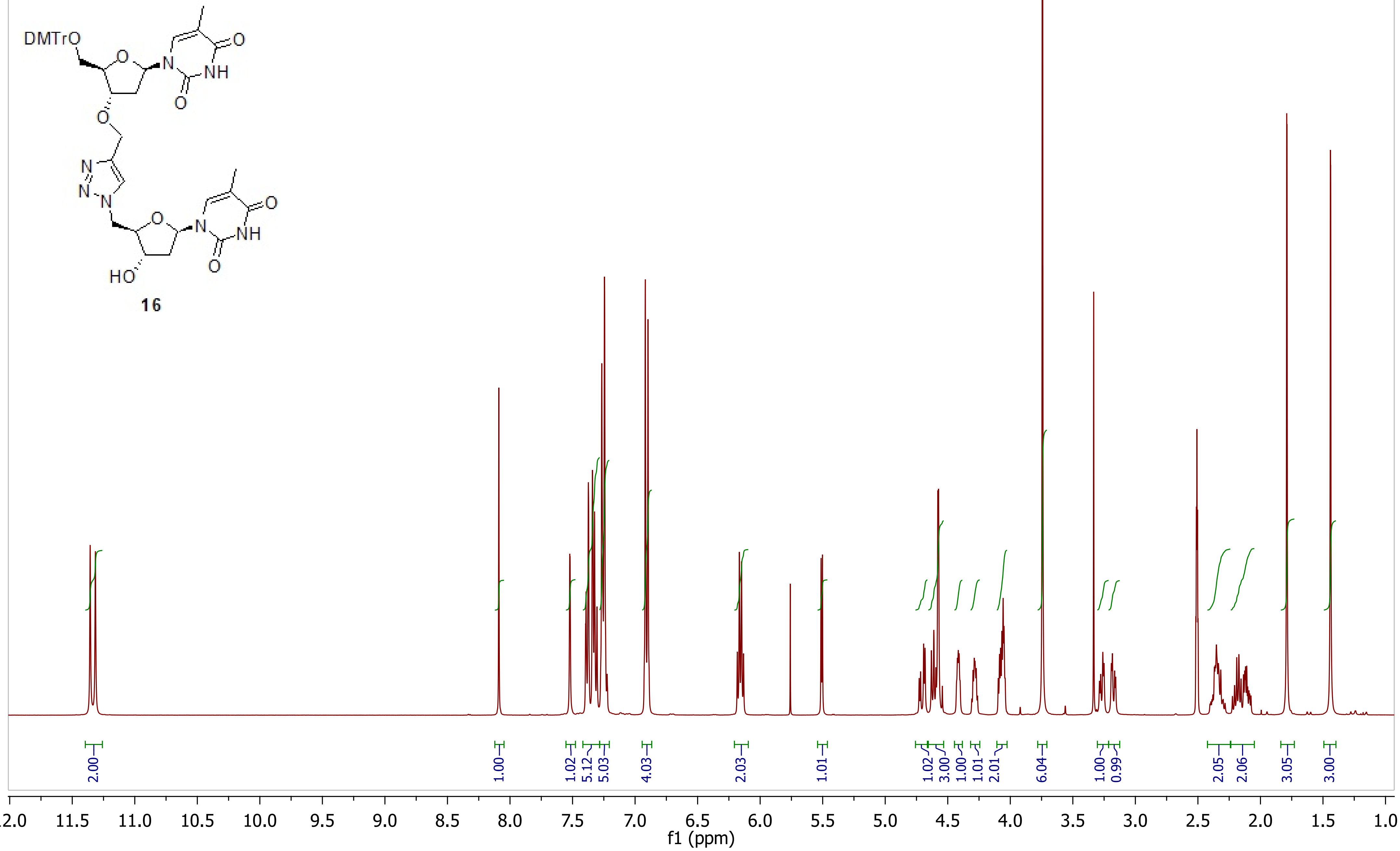

m_pal.MJP5-471
UserID m_pal SampleID MJP 5-471 - col - DMSO SupervisorID hayes Lab Phone No. 13536 Slot Number 13

Recorded on a Bruker AV-3-400 in d6-DMSO
^{13}C NMR recorded at 101 MHz

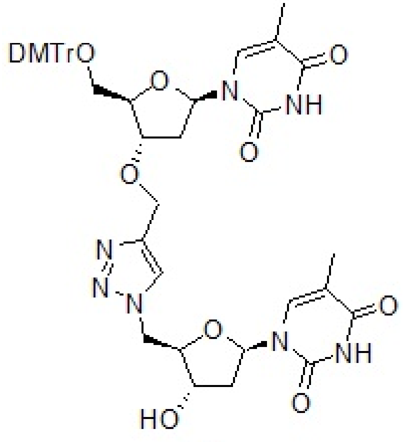

16

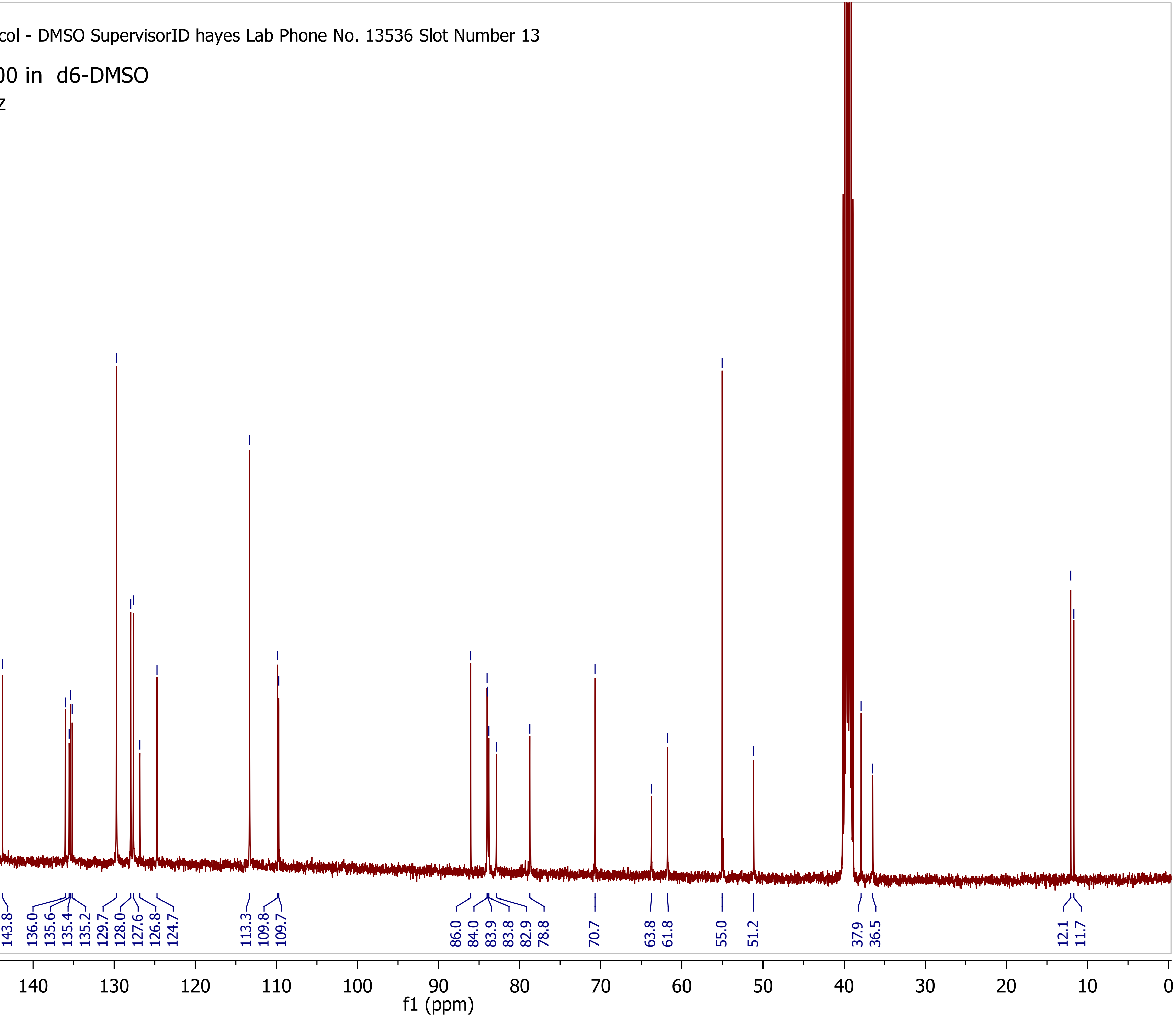

m_pal.MJP5-471
UserID m_pal SampleID MJP 5-471 - col - DMSO SupervisorID hayes Lab Phone No. 13536 Slot Number 13

Recorded on a Bruker AV-3-400 in d6-DMSO
DEPT 135 NMR recorded at 101 MHz

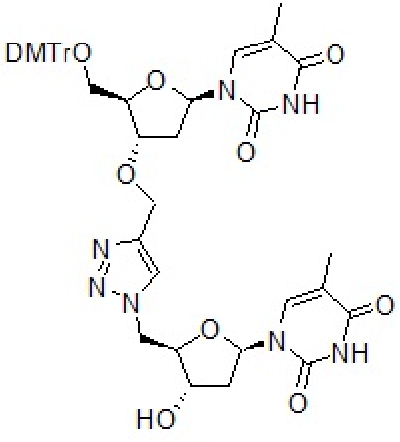

16

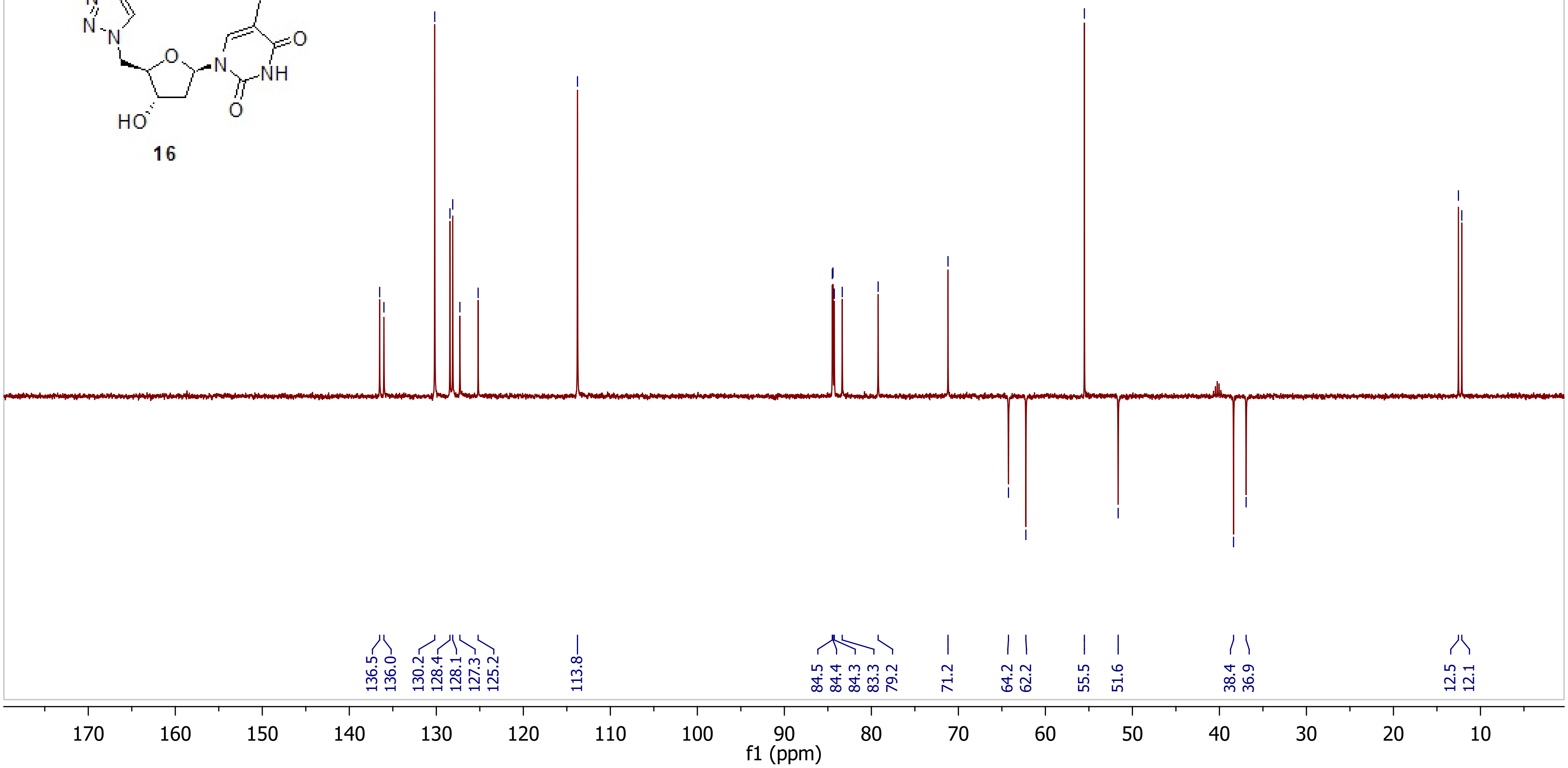

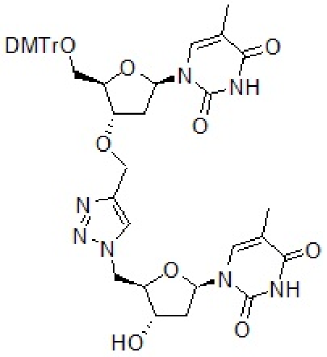

16

COSY NMR Recorded on a Bruker AV-3-400 in d6-DMSO

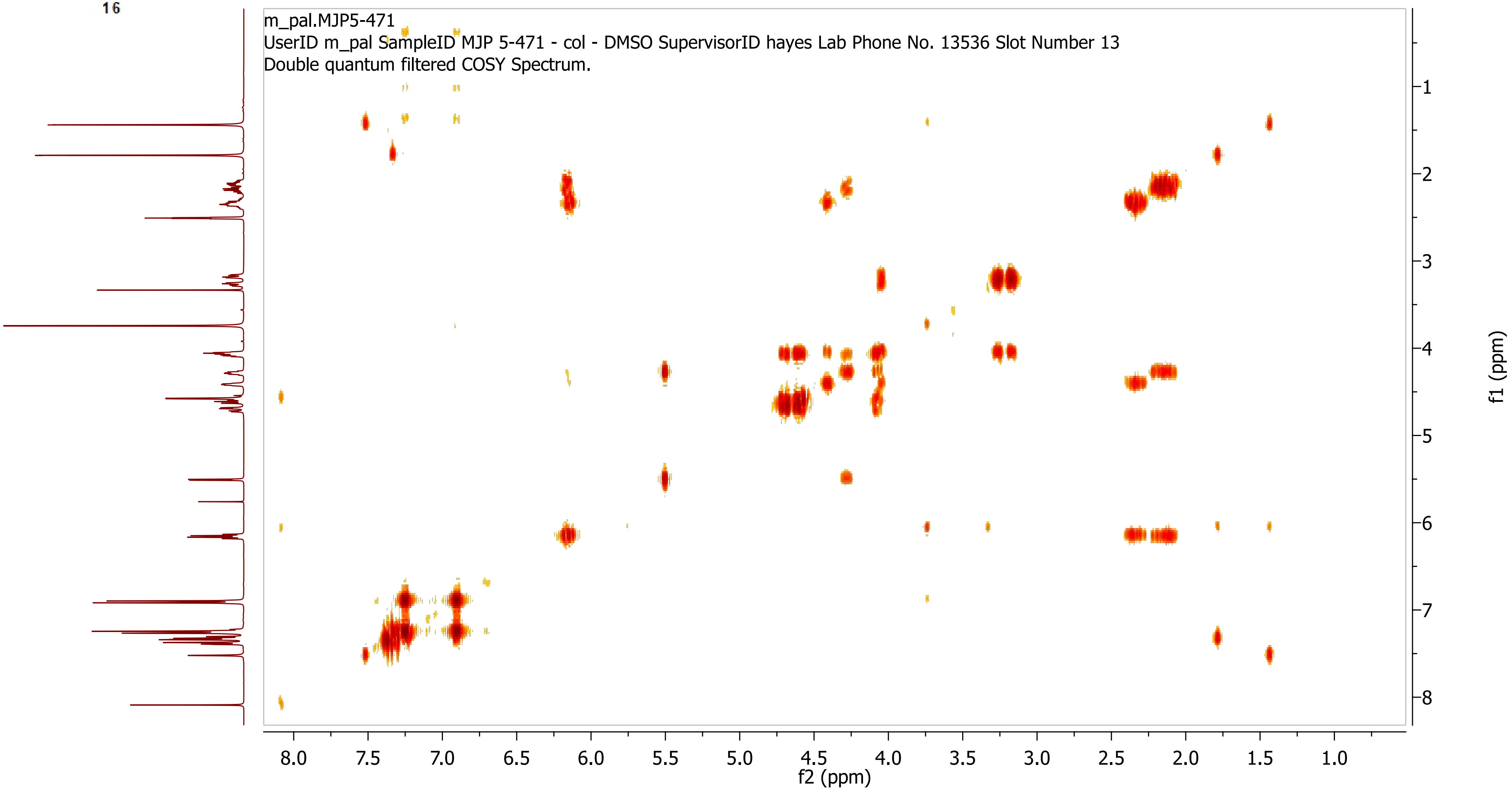

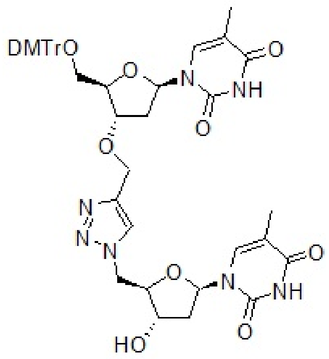

16

DEPT editted HSQC recorded on a Bruker AV-3-400 in d6-DMSO

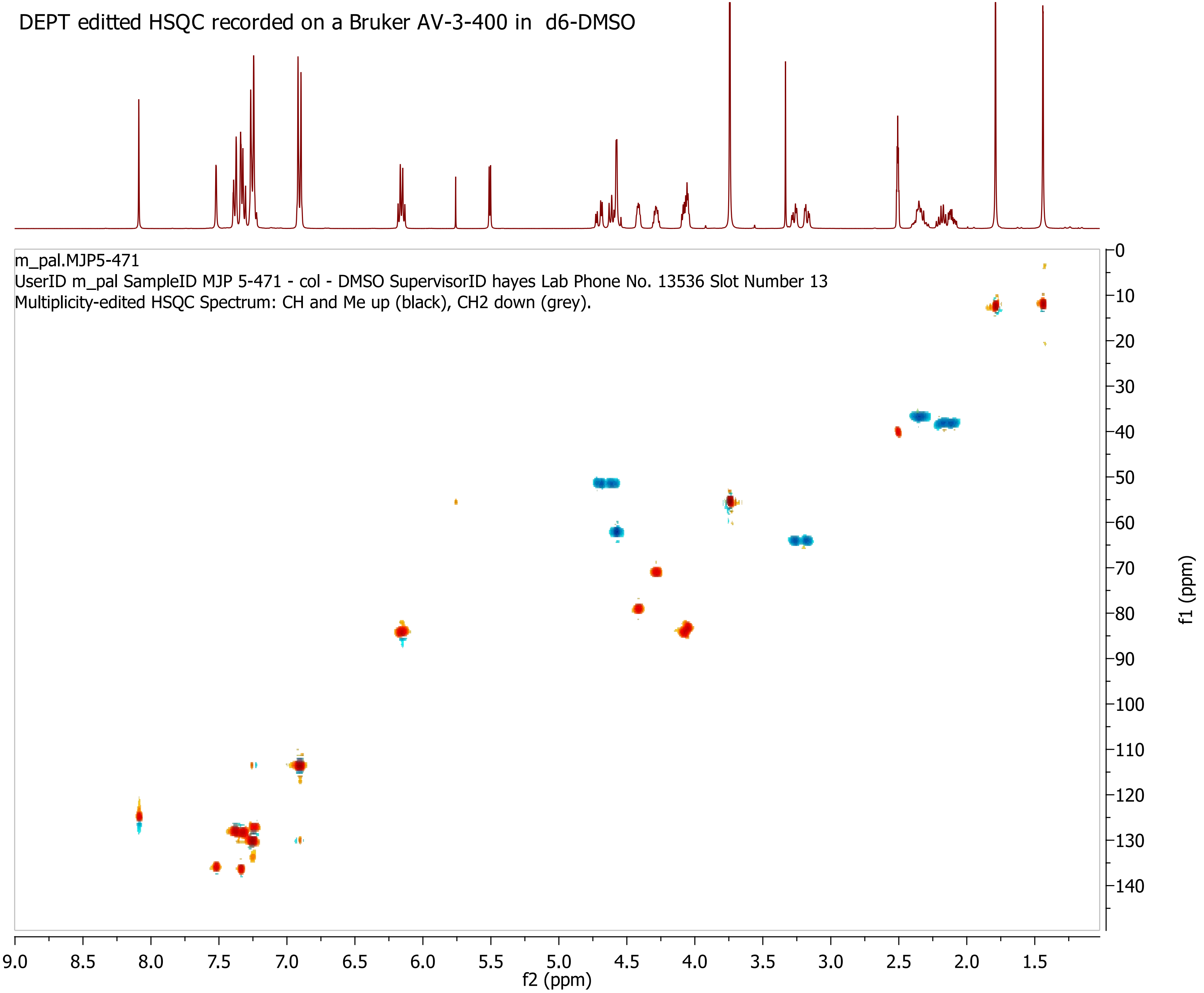

HMBC recorded on a Bruker AV-3-400 in d6-DMSO

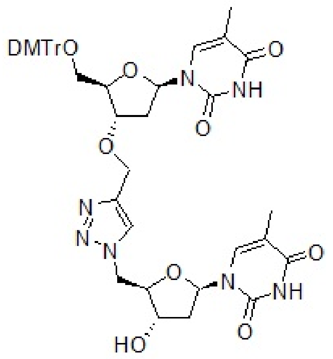

16

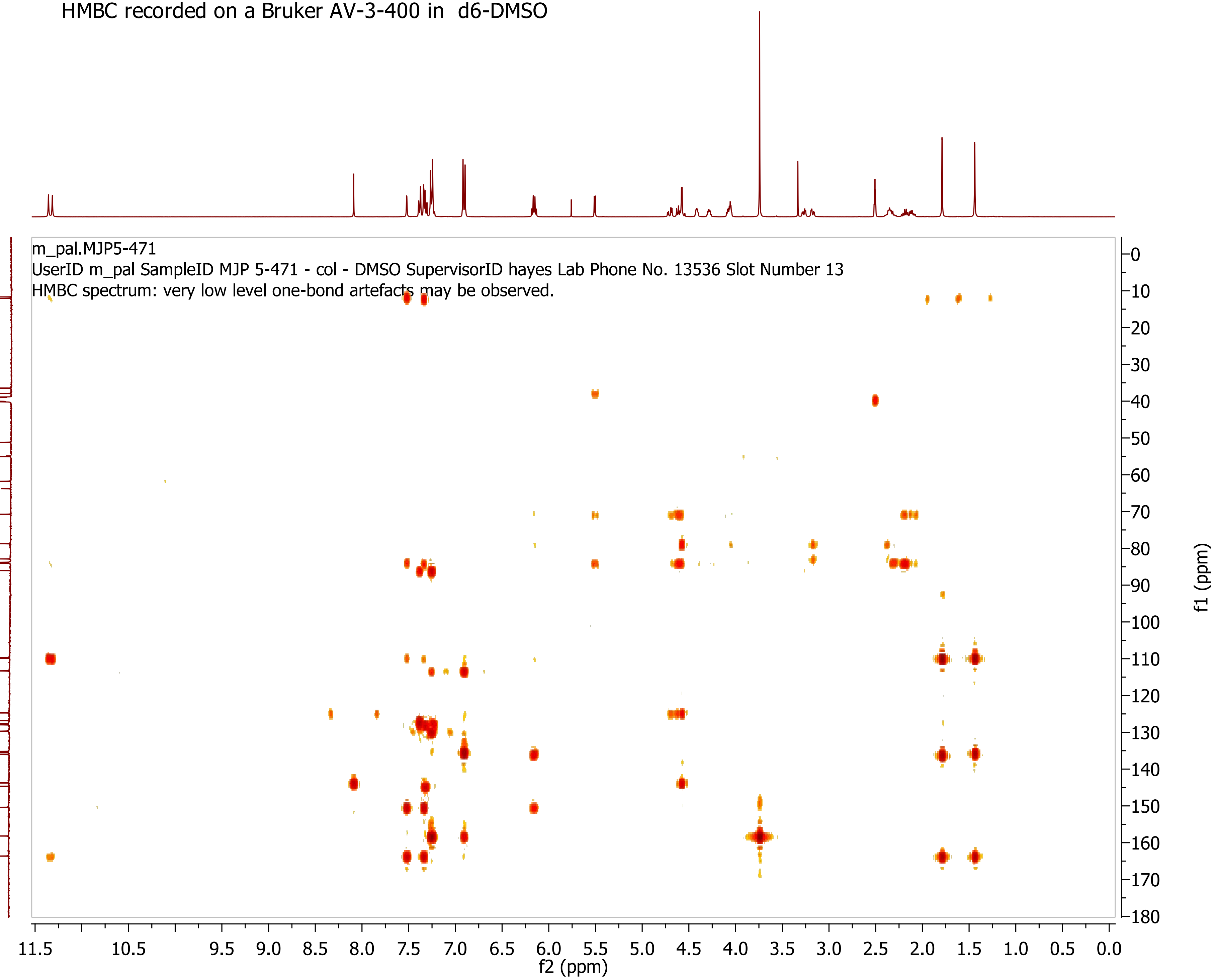

m_pal.MJP-6-563.1.fid
UserID m_pal SampleID MJP-6-563 SupervisorID gpatt Lab Phone No. 14190 Slot 4

Recorded on a Bruker AV-3-500 in $CDCl_3$
^1H NMR recorded at 500 MHz
Contains 2-cyanoethyl N,N-dipropylphosphonamidate

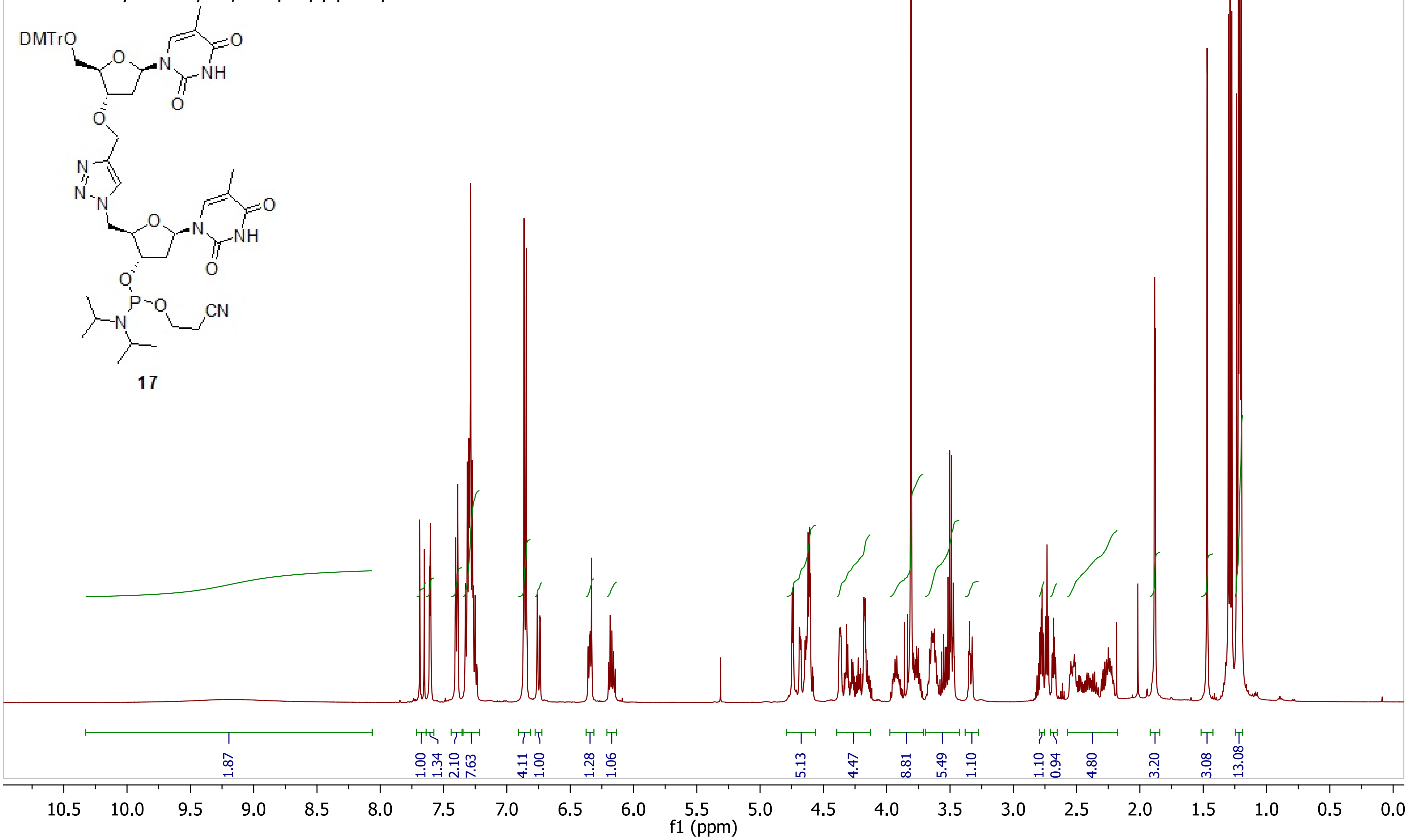

m_pal.MJP6-563.103.fid

UserID m_pal SampleID MJP 6-563 Brown SupervisorID gpatt Lab Phone No. 14190 Slot Number 31

Recorded on a Bruker AV400 in $CDCl_3$

^{13}C NMR recorded at 101 MHz

Contains 2-cyanoethyl N,N-dipropylphosphonamidate

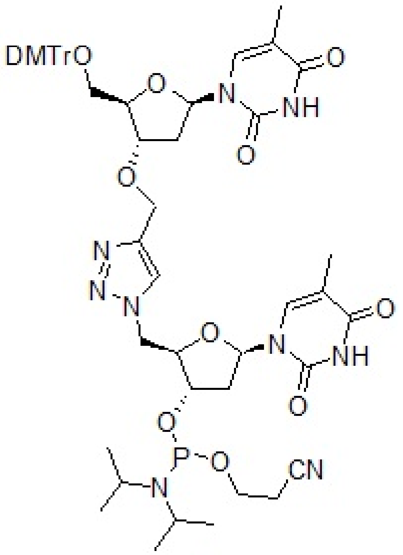

17

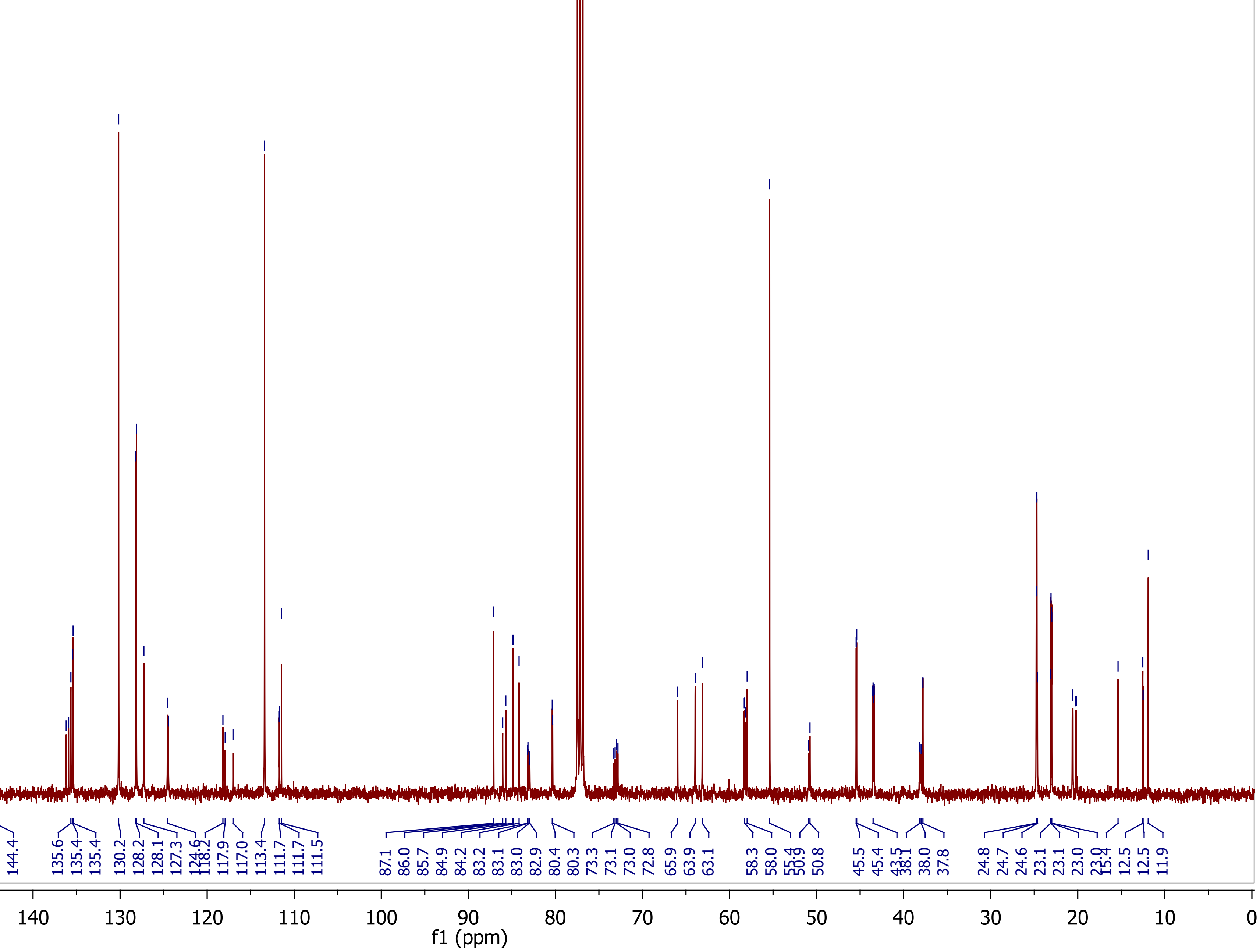

m_pal.MJP6-563.104.fid

UserID m_pal   SampleID MJP 6-563 Brown   SupervisorID gpatt   Lab Phone No. 14190   Slot Number 31

Recorded on a Bruker AV400 in $CDCl_3$

DEPT 135 recorded at 101 MHz

Contains 2-cyanoethyl N,N-dipropylphosphonamidate

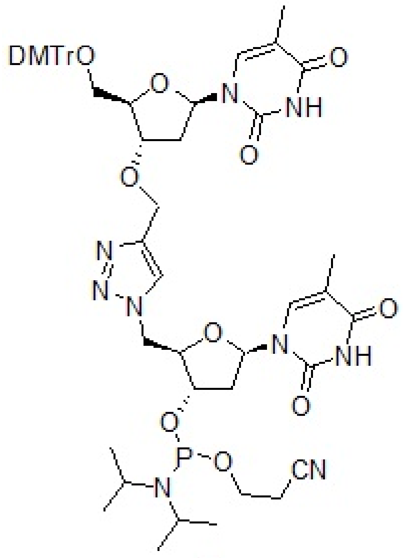

17

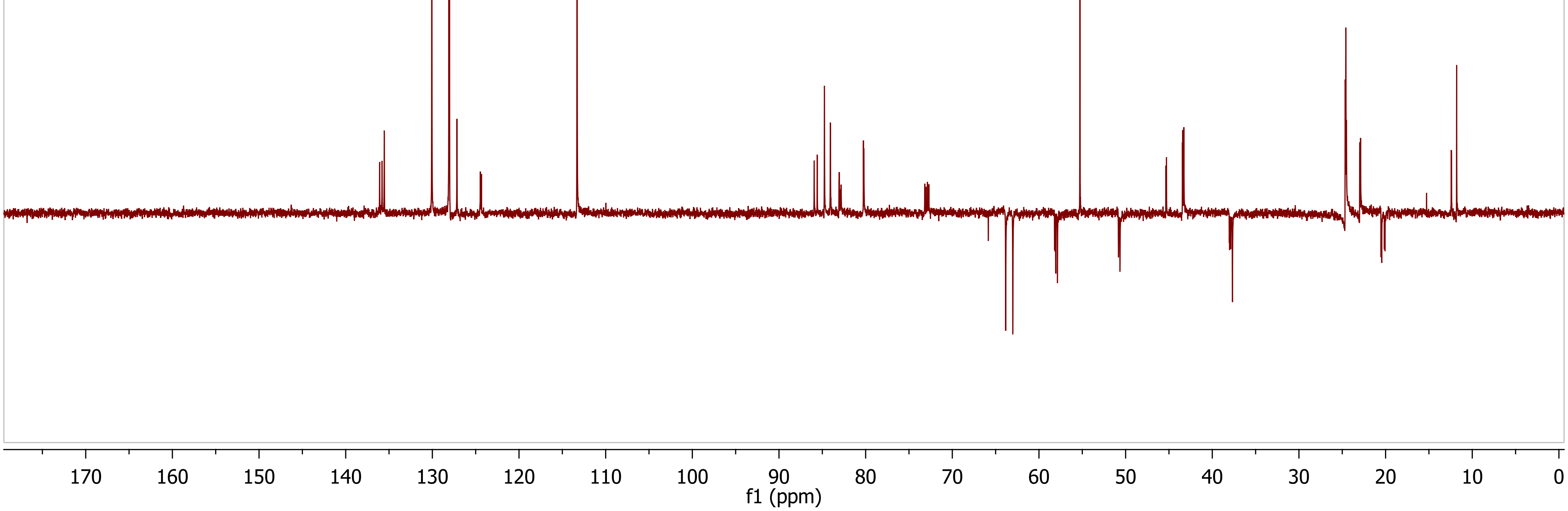

COSY Recorded on a Bruker AV400 in $CDCl_3$
Contains 2-cyanoethyl N,N-dipropylphosphonamidate

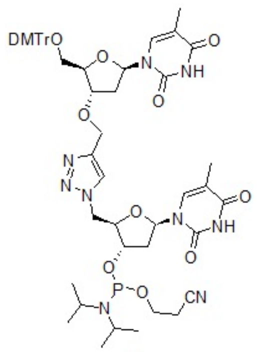

17

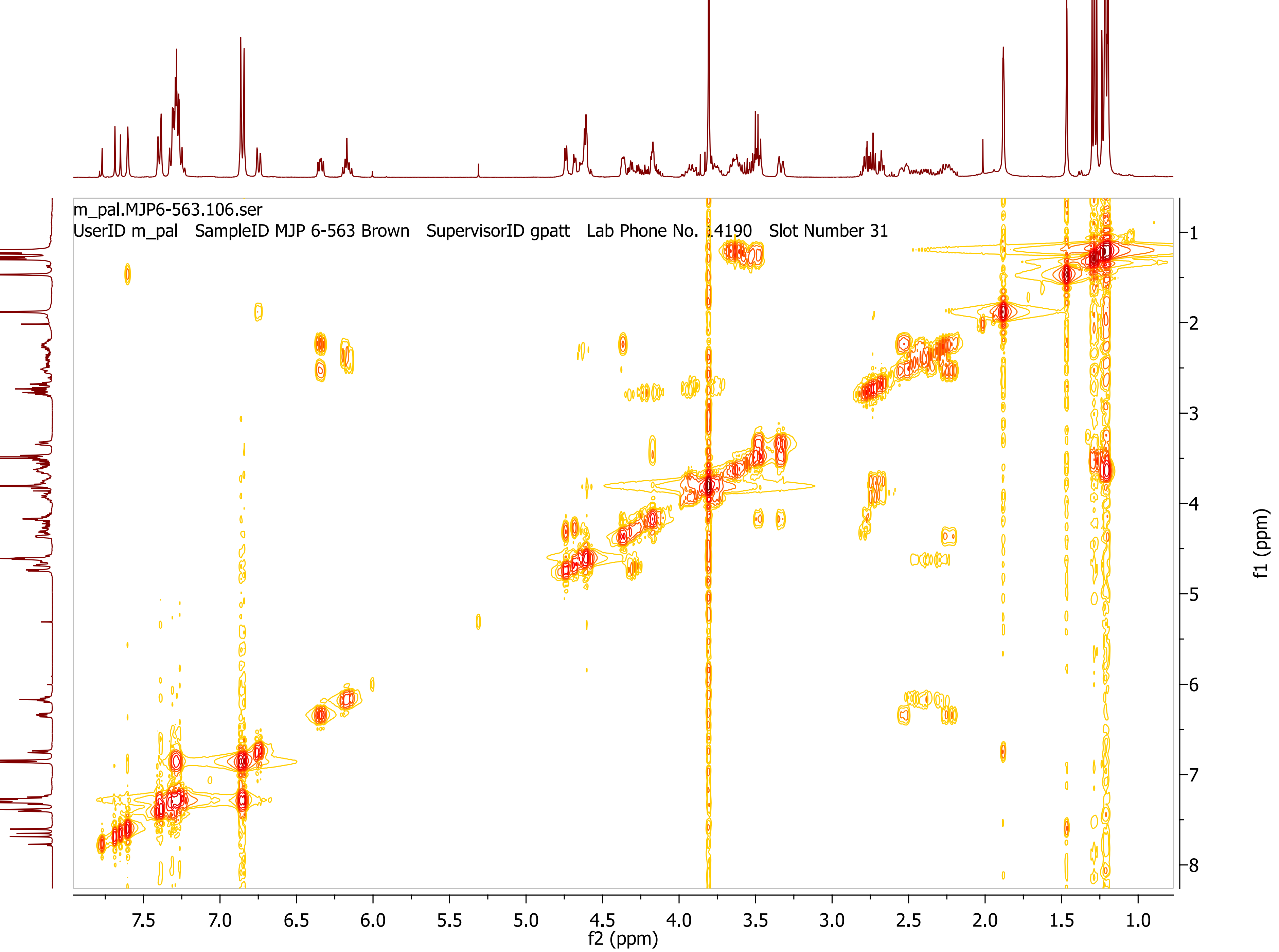

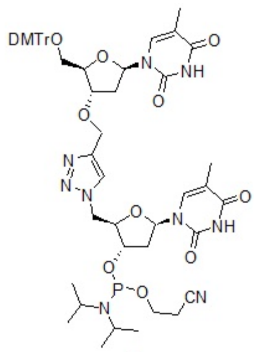

DEPT editted HSQC Recorded on a Bruker AV-3-500 in $CDCl_3$
Contains 2-cyanoethyl N,N-dipropylphosphonamidate

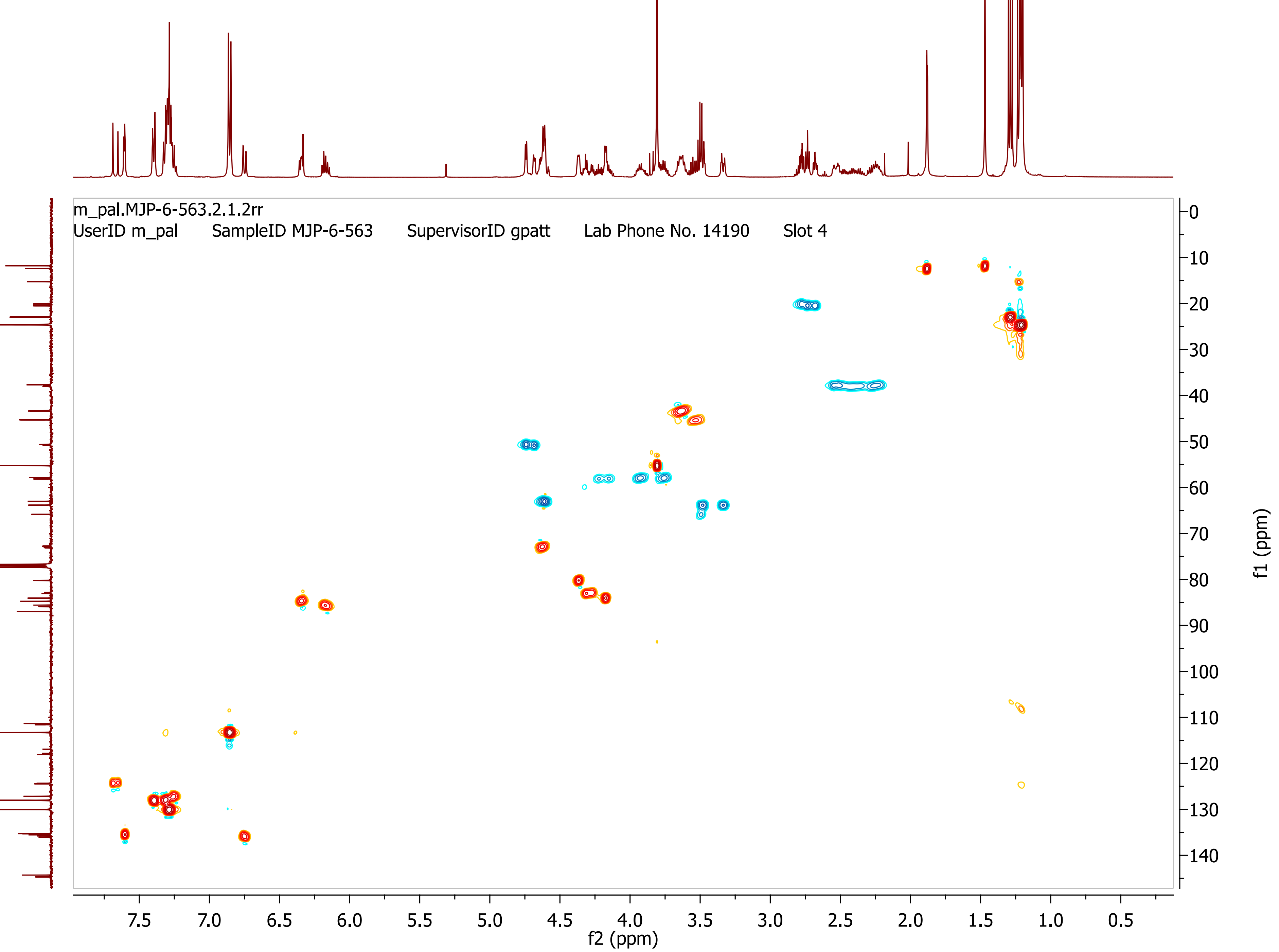

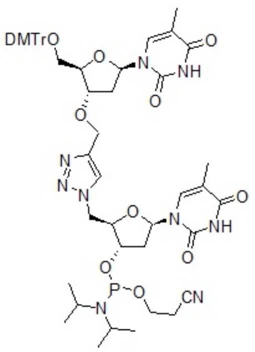

17

HMBC Recorded on a Bruker AV-3-500 in $CDCl_3$
Contains 2-cyanoethyl N,N-dipropylphosphonamidate

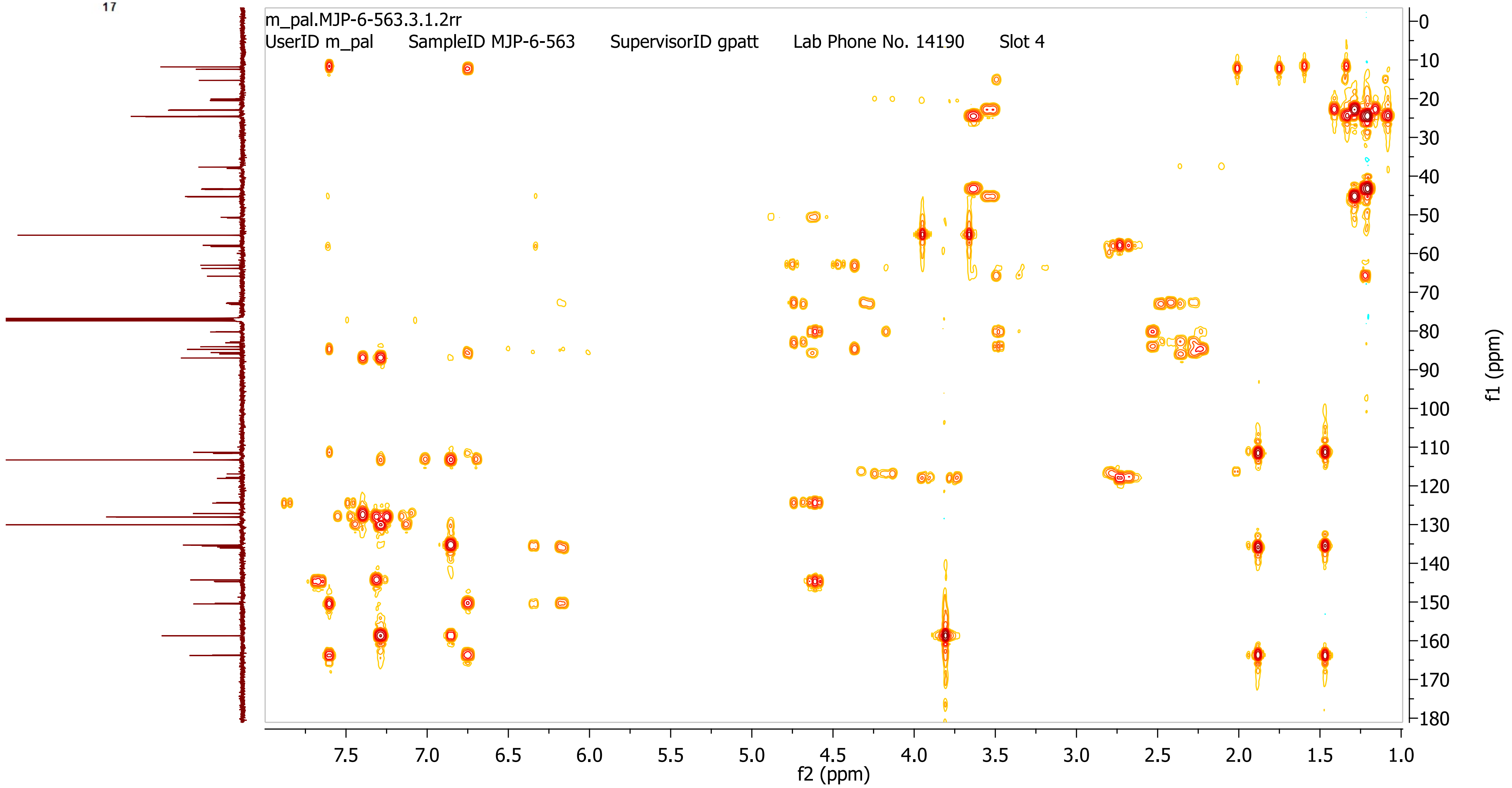

m_pal.MJP6-563.105.fid
UserID m_pal   SampleID MJP 6-563 Brown   SupervisorID gpatt   Lab Phone No. 14190   Slot Number 31
Recorded on a Bruker AV400 in $CDCl_3$
^{31}P NMR recorded at 162 MHz
Contains 2-cyanoethyl N,N-dipropylphosphonamidate

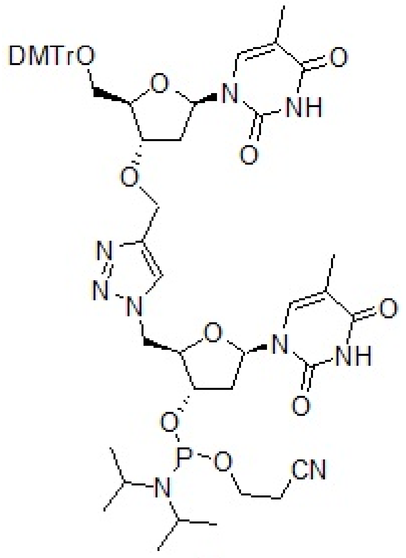

17

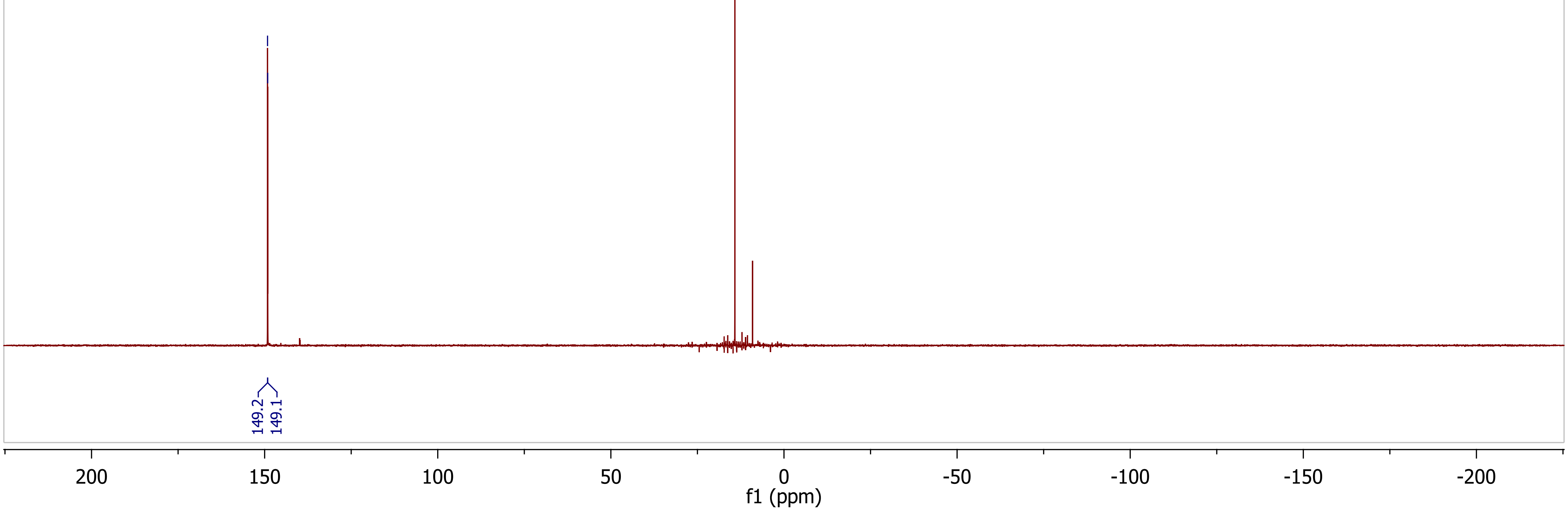

Supplement: Supplementary file 1 [file OB-014-C6OB00007J-s001.pdf]
